# The presynaptic protein bassoon is a biofluid biomarker of synaptic pathology in multiple sclerosis

**DOI:** 10.1016/j.ebiom.2026.106282

**Published:** 2026-05-07

**Authors:** Marcel S. Woo, Nicola Rothammer, Lukas C. Bal, Christoph Krisp, Mario Kreutzfeldt, Susanne Witt, Aleksandra Maleska Maceski, Bente Siebels, Lukas Raich, Christina Mayer, Ingo Winschel, Anne Willing, Benjamin Schattling, Simone Bauer, Hartmut Schlüter, Sina C. Rosenkranz, Jens Kuhle, Jan-Patrick Stellmann, Jan Broder Engler, Doron Merkler, Manuel A. Friese

**Affiliations:** a Institute of Neuroimmunology and Multiple Sclerosis, University Medical Center Hamburg-Eppendorf, Hamburg, Germany; b Section of Mass Spectrometry and Proteomics, University Medical Center Hamburg-Eppendorf, Hamburg, Germany; c Division of Clinical Pathology, Geneva University Hospital, Geneva, Switzerland; d Department of Pathology and Immunology, University of Geneva, Geneva, Switzerland; e Protein Production Core Facility, University Medical Center Hamburg-Eppendorf, Hamburg, Germany; f MS Center, Neurology and Research Center for Clinical Neuroimmunology and Neuroscience Basel, Departments of Clinical Research and Biomedicine, University Hospital and University Basel, Basel, Switzerland; g CEMEREM, APHM, Hôpital de la Timone, Marseille, France; h CRMBM, Aix Marseille University, CNRS, Marseille, France

**Keywords:** Multiple sclerosis, Bassoon, Neurodegeneration, Biomarker, Synaptic loss

## Abstract

**Background:**

Neuroaxonal and synaptic loss are hallmarks of multiple sclerosis (MS), the most common autoimmune disorder of the central nervous system. However, it remains unclear at which disease stages synaptic pathology occurs. We hypothesised that synaptic proteins in plasma and cerebrospinal fluid (CSF) reflect synaptic injury in MS.

**Methods:**

To identify synaptic proteins lost during neuroinflammation, we performed proteomic analysis of synaptoneurosomes from mice with experimental autoimmune encephalomyelitis (EAE), the model of MS. The findings were validated by histology in the cortex of EAE mice and postmortem MS tissue. Next, we developed an ELISA with knockout-validated antibodies for the presynaptic protein Bassoon (BSN) and quantified BSN in the cortex, spinal cords and plasma of EAE mice and in the CSF (total *n* = 30) and serum (total *n* = 146) of an observational cohort study with people with MS (pwMS) and controls. We further compared longitudinal trajectories of serum BSN (sBSN) and serum neurofilament light chain (sNfL) in a cohort of people with primary progressive MS (PPMS) (*n* = 26) using linear mixed-effects models.

**Findings:**

The synaptoneurosome screen revealed reduced levels of several presynaptic proteins, including BSN, in the cortex of EAE mice. The loss of synaptic BSN was validated in EAE and human postmortem tissues of pwMS. Notably, BSN simultaneously accumulated in neuronal soma during EAE and MS, suggesting that BSN may serve as a suitable biomarker for monitoring disease pathology. Our ELISA showed a gradual loss of BSN in the cortex of EAE mice and consistently, plasma BSN levels were elevated in two independent acute and chronic EAE cohorts. In pwMS, BSN was detectable in all CSF samples and in 81% of the serum samples. CSF BSN levels were higher in both relapsing and PPMS compared with controls, whereas sBSN was elevated in secondary progressive MS (SPMS) and PPMS. In the longitudinal PPMS cohort, sBSN and sNfL remained unchanged over an average follow-up of 37 months and did not correlate with each other.

**Interpretation:**

In conclusion, the presynaptic protein BSN can be quantified in plasma and CSF to assess synaptic pathologies. BSN elevation was already detectable at the earliest disease stages and persisted in progressive MS, underscoring continuous neurodegeneration in MS. Measuring synaptic proteins may complement established biomarkers of neuronal injury to enhance our understanding of neurodegeneration in MS.

**Funding:**

This work was funded by the Hamburg Innovation Call for Transfer (C4T959 to M.A.F.), 10.13039/501100007458Deutschen Multiple Sklerose Gesellschaft (V6.2 to M.A.F.). This work is supported by the 10.13039/501100001659Deutsche Forschungsgemeinschaft (FOR 5705, 523862973 to M.A.F., S.C.R., J.B.E.; 247354600, 247377969, 426788273, 518551069, 516868494 to H.S.).

**Research in context**

**Evidence before this study**

Synaptic loss is a key contributor to neurodegeneration and disability in multiple sclerosis (MS). While neurofilament light chain (NfL) and glial fibrillary acidic protein (GFAP) are established biomarkers of axonal and astrocytic injury, specific biomarkers of synaptic degeneration remain an unmet need. Positron emission tomography (PET) studies have demonstrated cortical synapse loss in early MS but they remain costly and not widely accessible. In other neurodegenerative diseases, synaptic proteins have been detected in cerebrospinal fluid (CSF) and plasma, indicating their potential as biomarkers of synaptic pathology. The presynaptic protein Bassoon (BSN) accumulates in stressed neurons and promotes neurodegeneration in mouse experimental autoimmune encephalomyelitis (EAE) and MS brains; however, whether BSN could serve as a fluid biomarker in MS was previously unknown.

**Added value of this study**

We developed a knockout-validated ELISA for BSN and quantified it in EAE and MS. Proteomic and histological analyses revealed loss of synaptic BSN and its somatic accumulation in the EAE and MS cortex. Plasma BSN levels were elevated in both acute and chronic EAE, and CSF and serum BSN levels were increased in MS. In primary progressive MS, serum BSN remained stably elevated and was not associated with NfL, suggesting that BSN reflects a complementary aspect of neurodegeneration related to synaptic injury.

**Implications of all the available evidence**

BSN represents a promising biomarker for monitoring synaptic pathology in MS. Its detection in accessible biofluids provides a translational tool for assessing cortical synaptic pathologies. Incorporating BSN into multimodal biomarker panels may improve disease staging, therapeutic monitoring, and understanding of neurodegeneration in MS.

## Introduction

Multiple sclerosis (MS) is the most common autoimmune disorder of the central nervous system (CNS).^1^ Its pathophysiology has been attributed to T and B cell infiltration, followed by myeloid cell activation and subsequent demyelination. However, accumulating evidence indicates that neuronal loss and synaptic pathology are key hallmarks of MS that correlate most strongly with the degree of disability.^2^ Notably, disability accumulation can already be detected in the earliest disease phases of relapsing MS (RMS) and appears to be driven by progression independent of relapse activity,^3^, ^4^, ^5^ underscoring that neuroaxonal pathology begins early and has clinical consequences. Despite these clinical observations, monitoring neuroaxonal pathology in people with MS (pwMS) remains challenging.

Persistent, compartmentalised, myeloid cell-driven, low-grade smouldering inflammation within the CNS is the major driver of demyelination, axonal injury, synaptic loss, and neuronal cell death. However, neuronal injury itself also perpetuates this chronic inflammatory state by releasing cell death-associated danger signals within the CNS.^2^^,^^6^ Although postmortem and mouse studies have clearly shown their pivotal relevance to clinical disability during chronic CNS inflammation,^7^, ^8^, ^9^ detecting these processes in the earliest disease phases remains difficult.

Neurofilament light chain (NfL) has emerged as a robust biomarker of neuroaxonal injury, measurable in both cerebrospinal fluid (CSF) and blood.^10^ Elevated NfL levels can be detected several years before the onset of clinical symptoms,^11^^,^^12^ reflecting subclinical axonal pathology. However, NfL predominantly indicates acute axonal injury rather than the chronic, smouldering inflammation-associated damage characteristic of progressive MS (PMS).^13^ In contrast, glial fibrillary acidic protein (GFAP), an astrocyte-specific structural protein and marker of astrogliosis, has been identified as a biomarker of disease progression and neurodegeneration in both RMS and PMS.^13^, ^14^, ^15^, ^16^

Recent studies have also used positron emission tomography (PET) to quantify synaptic density loss during the early and progressive stages of MS.^17^^,^^18^ Although PET remains the gold standard for *in vivo* assessment of pathological processes, its limited availability and high cost restrict its routine clinical application. Notably, synaptic proteins are released into the CSF and blood and have been used as body-fluid biomarkers of synaptic loss in neurodegenerative diseases such as Alzheimer’s disease.^19^ However, there remains an unmet need for complementary and accessible methods to capture ongoing synaptic pathology in MS.

Emerging evidence suggests that synaptic pathology in MS may not only be driven by synapse loss but also by functional disturbance in axonal transport, leading to the redistribution and accumulation of synaptic proteins. We previously identified that the presynaptic protein Bassoon (BSN) accumulates in neuronal somata during experimental autoimmune encephalomyelitis (EAE), the MS mouse model, and in MS, thereby promoting neuronal loss.^20^ Our earlier work showed that intracellular accumulation of BSN induces proteasomal dysfunction and activates neuronal cell death pathways.^21^^,^^22^ We therefore hypothesised that dying or dysfunctional neurons release BSN, which could then be measured in CSF or plasma as a biomarker reflecting neuronal distress and synaptic pathology in MS. To test this hypothesis, we developed an enzyme-linked immunosorbent assay (ELISA) to quantify BSN in the CSF and plasma of EAE mice and pwMS across different disease stages.

## Methods

### Mice

All mice (C57BL/6J WT (The Jackson Laboratory); *Bsn*^−/−^^20^) were kept under specific pathogen-free conditions in the central animal facility of the University Medical Centre Hamburg-Eppendorf (UKE). We used adult mice (6–20 weeks old) of both sexes; mice were sex and age-matched in all experiments. No sex-specific differences were observed in any of the experiments; therefore, data from both sexes were analysed together (result not shown). Progressive EAE mice were defined by insufficient recovery from the acute disease phase with an end score >2. No experimental animals were excluded in this study. Blood was collected from the right ventricle immediately after sacrifice and was stored in EDTA-coated tubes. After 30 min incubation on ice, samples were centrifuged 3000×*g* for 10 min. The supernatant was used for subsequent experiments and stored at −80 °C.

### Experimental autoimmune encephalomyelitis

We immunised mice subcutaneously with 200 μg MOG_35–55_ peptide (Schafer-N) in complete Freund’s adjuvant (Difco, cat. no. DF0639-60-6) containing 4 mg mL^−1^ *Mycobacterium tuberculosis* (Difco, cat. no. DF3114-33-8). In addition, we injected 200 ng pertussis toxin (Calbiochem, cat. no. CAS70323-44-3) intraperitoneally (i.p.) on the day of immunisation and again 48 h later. We scored animals daily for clinical signs by the following system: 0, no clinical deficits; 1, tail weakness; 2, hind limb paresis; 3, partial hind limb paralysis; 3.5, full hind limb paralysis; 4, full hind limb paralysis and fore limb paresis; 5, premorbid or dead. Animals reaching a clinical score of 4 were euthanised in accordance with local animal welfare regulations. Investigators were blinded to genotype and treatment in the EAE experiments.

### Flow cytometry of neuronal nuclei

Nuclei from mouse spinal cords were isolated with the Nuclei Isolation Kit (Sigma–Aldrich; catalogue no. NUC101) according to the manufacturer’s protocol. Nuclei were stained with PI (1:2000), a primary labelled antibody directed against NeuN (1:500; Abcam, ab190565), and pCREB (1:500; Cell Signalling, #9198). Immunofluorescence was acquired on an LSR II FACS analyser (BD Biosciences). Data analysis was performed with the FlowJo v.10 analysis software (FlowJo LLC).

### Enzyme-linked immunosorbent assay

To detect the BSN protein in tissues and body fluids, a customised ELISA was established using a commercial kit (SimpleStep ELISA Custom ELISA Kit, Abcam, ab270552). A pair of antibodies was used, in which one was conjugated to a capture peptide (capture antibody) and the other to horseradish peroxidase (HRP; detection antibody), following the manufacturer’s instructions. The capture antibody was a polyclonal rabbit anti-Bassoon antibody (Synaptic Systems, Cat. No. 141,003), and the detection antibody was a monoclonal mouse anti-Bassoon antibody (clone SAP7F407, Enzo, Cat. No. ADI-VAM-PS003-F). Both were used at a concentration of 500 ng mL^−1^. Following conjugation, the antibodies were mixed in equal proportions and applied together with the samples to a 96-well plate supplied with the kit. For plasma samples from EAE mice and human serum and CSF, 50 μL per technical replicate was used. Brain lysates from *Bsn*^*−/−*^ and WT mice were prepared by homogenisation followed by 2-h incubation in lysis buffer (100 mM Tris, pH 7.4, 150 mM NaCl, 1 mM EGTA, 1 mM EDTA, 1% Triton X-100, 0.5% sodium deoxycholate, phosphatase inhibitor, protease inhibitor, 1 mM PMSF). A total of 25 μg protein per sample was analysed. The antibody-sample mixture was incubated for 1 h at room temperature with shaking, followed by three washes with the kit wash buffer. Detection reagent was then added and incubated for 15 min, followed by addition of the stop solution. Absorbance was measured at 450 nm using a microplate reader (Tecan Spark M). The positive control and all samples were measured in triplicate. To allow comparability across different plates, we included on each plate a dilution series of a recombinant BSN peptide (see below) with the following concentrations: 0 ng mL^−1^, 0.1 ng mL^−1^, 0.25 ng mL^−1^, 0.5 ng mL^−1^, 1 ng mL^−1^, 2.5 ng mL^−1^, 5 ng mL^−1^, 10 ng mL^−1^.

### Bassoon peptide generation

The positive control consisted of a fusion protein containing rat Bassoon segments 3569–3769 and 738–1035, connected via a GS4 linker. The construct also included a His-tag and a thrombin protease cleavage site. The recombinant protein was produced using the bacterial expression system pET28c(+). The final product had a theoretical molecular weight of 58.1 kDa and an isoelectric point (pI) of 5.97. The amino acid sequence was as follows: MGSSHHHHHHSSGLVPRGSHMHLGQEETDWFDKPRDARSDRFRHHGGHTVSSSQKRGPARHSYHDYDEPPEEGLWPHDEGGPGRHTSAKEHRHHGDHGRHSGRHAGEEPGRRAARPHARDMGRHETRPHPQASPAPAMQKKGQPGYPSSADYSQPSRAPSAYHHASDSKKGSRQAHSGPTVLQPKPEAQAQPQMQGRQAVPGPQQSQPPSSRQTPSGTASRGGGGSGGGGSGGGGSGGGGSPSGEWSKPPSGSAVEDQKRRPHSLSIMPEAFDSDEELGDILEEDDSLAMGRQREQQDTAESSDDFGSQLRHDYVEDSSEGGLSPLPPQPPARADMTDEEFMRRQILEMSAEEDNLEEDDTAVSGRGLAKHGAQKASARPRPESSQESVALPKRRLPHNATTGYEELLSEEGPAEPTDGALQGGLRRFKTIGLNSTGRLWSTSLDLGQGSDPNLDREPELEMESLTGSPEDRSRGEHSSTLPASTPSYTSGTSPTSLSSLEEDSDSSPSRRQRLEEAKQQRKARHRSHGPLLPTIEDS.

### Synaptoneurosome purification

Crude synaptoneurosomes were purified as described previously.^22^ Immediately after sacrifice, mice were perfused with ice-cold PBS, and cortices were removed and kept on ice. We added 3 mL HEPES-buffered sucrose (HBS; 320 mM sucrose, 4 mM HEPES pH 7.4, 2 mM EDTA, 2 mM EGTA, 1 mM PMSF, protease inhibitor cocktail (Roche)) per cortex, followed by homogenisation in a motor-driven glass Teflon homogeniser at 900 rpm with 10–15 strokes. A 100 μL aliquot was frozen as homogenate input. The remaining homogenate was centrifuged for 15 min with 950×*g* at 4 °C to remove the nuclear pellet. The supernatant was centrifuged again for 15 min with 10,000×*g* at 4 °C, and the resulting pellet was resuspended in 3 mL HBS and centrifuged once more under the same conditions. We resuspended the pellet in 2 mL hypoosmotic shock solution (2 mM EDTA, protease inhibitor cocktail (Roche)) by pipetting up and down. The suspension was rapidly adjusted to 4 mM HEPES by adding 8 mL 1 M HEPES and mixed continuously for 30 min at 4 °C. The lysate was then centrifuged with 20,000×*g* for 60 min at 4 °C. The pellet, representing the crude synaptoneurosome fraction, was used for subsequent proteomic analysis.

### LC-MS/MS-based proteomics of synaptoneurosomes

Sample preparation and measurement were performed similarly to previously described.^23^ Samples were lysed in 100 mM triethylammonium bicarbonate (TEAB) and 1% (w/v) sodium deoxycholate buffer, followed by probe sonication to degrade polynucleotides and heat-induced protein denaturation at 99 °C for 5 min. Protein concentrations were estimated with a bicinchoninic acid (BCA) protein assay (Thermo Fisher Scientific, Bremen, Germany). Equal amounts of protein were reduced in the presence of 10 mM dithiothreitol (Sigma Aldrich) for 30 min at 60 °C, followed by cysteine alkylation with 20 mM iodoacetamide (Sigma Aldrich) for 30 min at 37 °C in the dark, and subsequent enzymatic digestion with sequencing-grade trypsin (Promega) overnight at 37 °C. Digestion was quenched with 1% formic acid (FA), precipitated sodium deoxycholate was removed by centrifugation for 5 min at 14,000×*g*, and the supernatant was dried in a vacuum centrifuge. Peptides were labelled with four TMT 6-plex reagent kits (Thermo Fisher Scientific) according to the manufacturer’s instructions. A peptide pool was included as a control for each batch. Equal amounts of labelled peptides from all samples were pooled and subjected to high-pH reversed-phase fractionation. The combined sample was dissolved in 10 mM ammonium bicarbonate (NH_4_HCO_3_) to a final concentration of 1 μg μL^−1^, and 50 μg of peptides were fractionated on a 25 cm ProSwift™ RP-4H capillary monolithic column (Thermo Fisher Scientific) using an Agilent 1200 HPLC system. A 45-min gradient was applied at a flow rate of 0.2 mL per min, starting at 96.7% eluent A (10 mM NH_4_HCO_3_) and 3.3% eluent B (10 mM NH_4_HCO_3_ in 90% acetonitrile) for 5 min, increasing to 38.5% B over 20 min, then to 95% B for 10 min, followed by re-equilibration to 3.3% B for 8 min. Thirty fractions were collected using an ÄKTA Prime Plus fraction collector, pooled into thirteen fractions, and dried by vacuum centrifugation. TMT-labelled peptide fractions were separated on a 25 cm C18 reversed-phase column (BEH C18 Column, 130 Å, 1.7 μm, 75 μm × 250 mm, Waters Corporation, Milford, MS, USA) within a 60 min gradient from 2% to 30% acetonitrile (equilibration buffer: 0.1% formic acid; elution buffer: 99.9% acetonitrile and 0.1% formic acid) buffer at a flow rate of 250 nL per minute using a nano-UPLC system (Acquity, Waters Corporation, Milford, MS, USA) equipped with full recovery autosampler vials. This was coupled via electrospray ionisation (ESI) to a tandem mass spectrometer equipped with a quadrupole and an Orbitrap (Q Exactive, Thermo Fisher Scientific, Bremen, Germany), operated in MS/MS mode using data-dependent acquisition (DDA). The 12 most intense ions per precursor scan (AGC target: 3 × 10^6^ ions; resolution: 70,000; fill time: 50 ms) were selected for MS/MS (HCD at 32 normalised collision energy; AGC target: 5 × 10^3^ ions: resolution: 17,500; fill time: 100 ms) within a mass range of 400–1300 m/z and a fixed MS2 start mass of m/z 100. A dynamic precursor exclusion of 20 s was applied. TMT-labelled raw data were analysed using MaxQuant (v.1.6.2.10) with the integrated Andromeda search engine. Spectra were searched against the *Mus musculus* SwissProt database (17,013 proteins, January 2019). Trypsin was specified as the digestion enzyme, allowing up to two missed cleavages. Carbamidomethylation (C) was set as a fixed modification, while oxidation (M), protein N-terminal acetylation, and pyroglutamate formation were set as variable modifications. TMT 6-plex labels were applied to lysine residues and peptide N-termini for quantification. Error-rate control was performed at a 1% false discovery rate (FDR) at the peptide-spectrum match, peptide, and protein levels.

### Immunohistochemistry of mouse tissue

Mouse spinal cord tissue and cortical tissues were obtained and processed as described previously.^9^ Images were acquired using a confocal LSM 700 or LSM 900 Airyscan 2 laser scanning confocal microscope (Zeiss). Images were analysed using Fiji (ImageJ). Synapses were defined as BSN^+^ Homer1^+^ puncta. The following primary antibodies were used: BSN (SAP7F407, Enzo, Cat. No. ADI-VAM-PS003-F), Homer1 (Synaptic Systems, Cat. No. 160,011), MAP2 (abcam, Cat. No. ab5392), NeuN (Merck, Cat. No. MAB377).

### Immunohistochemistry of human tissue

Human tissue processing was performed using standard fixation and embedding methods. Briefly, human CNS tissue was fixed in 4% formalin, embedded in paraffin, and sectioned at 2 μm before mounting on glass slides. Tissue sections were then deparaffinised, and antigen retrieval was performed using citrate buffer (pH 6.0). Slides were incubated with Dako REAL peroxidase-blocking solution (Dako, K0672) to inactivate endogenous peroxidases, and non-specific binding was blocked (PBS with 2.5% goat serum) before overnight incubation with a rabbit anti-Bassoon primary antibody (Novus NBP1-80595). To visualise the specific signal, anti-rabbit HRP (Dako, K4003) with amplification (TSA Alexa Fluor 488, Life,T20948) was used as the secondary system. After washing, slides were incubated with Fab fragment goat anti-rabbit IgG (Jackson ImmunoResearch, 111-007-003) and PBS with 2.5% goat serum to prevent non-specific binding, followed by incubation with a rabbit anti-MBP antibody. After overnight incubation at 4 °C, specific binding was visualised using a donkey anti-rabbit IgG Alexa Fluor 555 (Life, A31572). Following additional washing and blocking with PBS with 2.5% goat serum, sections were incubated with a mouse anti-MAP2 antibody (Neomarkers, MS-249-50). After overnight incubation and washing, donkey anti-mouse Alexa Fluor 647 (Jackson ImmunoResearch, 715-605-151) was applied to visualise the signal. Nuclei were stained with DAPI (Invitrogen, D1306). Slides were mounted in Fluoromount aqueous mounting medium (Sigma–Aldrich, F4680) for image acquisition. Stained sections were scanned using a Pannoramic P250 Flash II whole-slide scanner at a resolution of 0.325 μm per px. Regions of interest were manually delineated and analysed using a custom pipeline in Visiopharm (Visiopharm Denmark, Version 2024.7.2). A U-Net-based classifier was trained to identify neurons, MAP2- and Bassoon-positive structures, and a border region of 2 μm was defined around neurons and MAP2 signals. Finally, the number and total area of neurons, MAP2 and Bassoon signals were quantified across the total tissue area, and the number of Bassoon signals within neuronal and border regions.

### Patient cohorts

People with MS and controls were recruited through the MS outpatient clinic of the Department of Neurology, University Medical Centre Hamburg-Eppendorf, Hamburg, Germany, in an observational cohort study. The participants were classified as relapsing-remitting MS (RRMS), secondary progressive MS (SPMS) or primary progressive MS (PPMS) according to the 2017 McDonald criteria by board-certified neurologists. All people with relapsing MS were sampled during remission. The control group consisted of people with non-inflammatory neurological conditions. Serum and CSF samples were collected and processed according to the standard operating procedures of the UKE Institute of Neuroimmunology and Multiple Sclerosis (INIMS). Samples were cryopreserved at −80 °C in the INIMS biobank. Serum NfL levels were measured using SiMOA according to the manufacturer’s instructions. Age-adjusted NfL Z scores were calculated^24^ and used for subsequent analyses. The longitudinal PPMS cohort has been described in detail elsewhere.^25^ Whole brain and cortex volumes were calculated applying the longitudinal FreeSurfer pipeline (https://surfer.nmr.mgh.harvard.edu) for brain segmentation to isotropic 0.9 mm MPRAGE images acquired on a 3T MRI system.^26^ For all analyses, volumes were corrected for head size as indicated by the total intracranial volume. All included patients with PPMS did not show any baseline inflammatory activity and were untreated.

### Statistical analysis

All analyses were conducted in R (v4.4.1). The following packages were used for analysis and visualisation: tidyverse, lme4,^27^ tidyplots,^28^ and clusterProfiler.^29^

Proteomics data were normalised to a peptide pool comprising equal amounts of peptides from each sample. This pool was included as one condition in all runs of the TMT 6-plex kit, allowing standardisation across batches (see above “LC-MS/MS-based proteomics of synaptoneurosomes” for detailed description of the proteomics processing). Linear models were fitted for each protein; multiple testing was controlled using Storey’s q-value FDR correction. Gene ontology cellular component analysis of the 200 most abundant proteins identified in synaptoneurosomes was performed using clusterProfiler with a minimal gene set size of 20. FDR correction was performed to account for multiple comparisons.

Group comparisons of sBSN or CSF BSN were performed with unpaired parametric or non-parametric tests, as specified in the respective figure legends. In addition, linear models were fitted including age, sex, disease duration, Expanded Disability Status Scale (EDSS), any immunomodulatory treatment and high-efficacy disease-modifying treatments as covariates. The significance levels from both approaches are shown in the respective figures.

We averaged the triplicates of the ELISA measurements. Because most measurements were outside the linear range of the positive control, we reported background-subtracted values and did not calculate absolute concentrations. Values below the detection limit were set to zero, to half of the limit of detection (LOD), or were estimated using multiple imputation by chained equations (MICE). Predictive mean matching was used to impute censored concentrations, and the imputation model included disease group, age and sex to preserve group structure and account for systematic sources of variation. Fifty imputed datasets were generated, each analysed using the prespecified regression model, and effect estimates and standard errors were pooled according to Rubin’s rules to obtain final estimates. The respective methods are indicated in the figure legends.

Assumptions of linear and linear mixed-effects models were evaluated before inference. Normality of residuals was assessed using the Shapiro–Wilk test, which indicated a normal distribution of residuals. For mixed-effects models, Q–Q plots of the random effects were additionally inspected and did not indicate departures from normality. Homoscedasticity in linear models was tested using the studentised Breusch–Pagan test, with no evidence of variance heterogeneity detected. Independence of observations in longitudinal analyses was accounted for by including subject-specific random intercepts. Sensitivity analyses using log10-transformed concentrations yielded comparable results, confirming robustness of the findings.

For the longitudinal PPMS cohort, we used linear mixed-effects models with age, sex at birth, EDSS, and disease duration as covariates to compare sBSN and sNfL z-score across the different time points. Longitudinal analyses were conducted using linear mixed-effects models with age, sex at birth, and baseline sBSN levels as covariates and participants as random intercepts to test associations between sBSN, EDSS, and cortical thickness over time. Visits were treated as continuous variable. To characterise longitudinal stability, between-subject and within-subject variance components were estimated using a random-intercept linear mixed-effects model, and the intraclass correlation coefficient was calculated as the proportion of total variance attributable to inter-individual differences. Correlations were calculated using two-sided Pearson’s correlation coefficient. *P* < 0.05 was considered statistically significant.

### Ethics

All animal care and experimental procedures were performed in accordance to institutional guidelines and conformed to the requirements of the German legal authorities. Ethical approval was obtained from the State Authority of Hamburg, Germany (approval No. 122/17).

The human cohort study was approved by the local ethics committee (Hamburg Chamber of Commerce Act for the Health Professions, registration number PV4405, Biobank Hamburger Patienteninformationssystem Multiple Sklerose - HAPIMS). Informed consent was obtained from all participants. The study adheres to the 2024 Declaration of Helsinki ethical principles.

### Role of funders

This work was funded by the Hamburg Innovation Call for Transfer (C4T959 to M.A.F.), Deutsche Multiple Sklerose Gesellschaft (V6.2 to M.A.F.). This work is supported by the Deutsche Forschungsgemeinschaft (FOR 5705, 523862973 to M.A.F., S.C.R., J.B.E.; 247354600, 247377969, 426788273, 518551069, 516868494 to H.S.). The funders had no role in the study design, data collection, data analyses, interpretation, or writing of report. Authors were not precluded from accessing data in the study, and they accept responsibility to submit for publication.

## Results

### Synaptic BSN is reduced in EAE and MS

We set out to quantify synaptic loss *in vivo* across different disease stages in pwMS. Based on evidence from other neurodegenerative diseases, we hypothesised that synaptic proteins in body fluids could serve as biomarkers of synaptic pathology.^30^^,^^31^ To identify a suitable candidate, we first examined which synaptic proteins are lost in EAE.

We focused on the cortex because we detected neuronal loss (Supplementary Fig. S1A; two-sided Mann–Whitney U test; NeuN count, *P* = 0.018; cortical thickness, *P* = 0.030) and a reduction in phosphorylated cAMP-response element binding protein (pCREB), a hallmark of neuronal excitotoxicity and injury,^32^ in cortical neurons (Supplementary Fig. S1B; two-sided Mann–Whitney U test, *P* = 0.010), whereas the T cell infiltration was largely restricted to the proximity of meninges (Supplementary Fig. S1C; two-sided Mann–Whitney U test; total brain, *P* = 0.022; cortex, *P* = 0.022; hippocampus, *P* = 0.022; periventricular zone, *P* = 0.0050). Thus, the cortex more closely resembles the smouldering pathology observed in the PMS cortex than the highly inflamed spinal cord typically seen in C57BL/6 EAE mice.

**Fig. 1:**
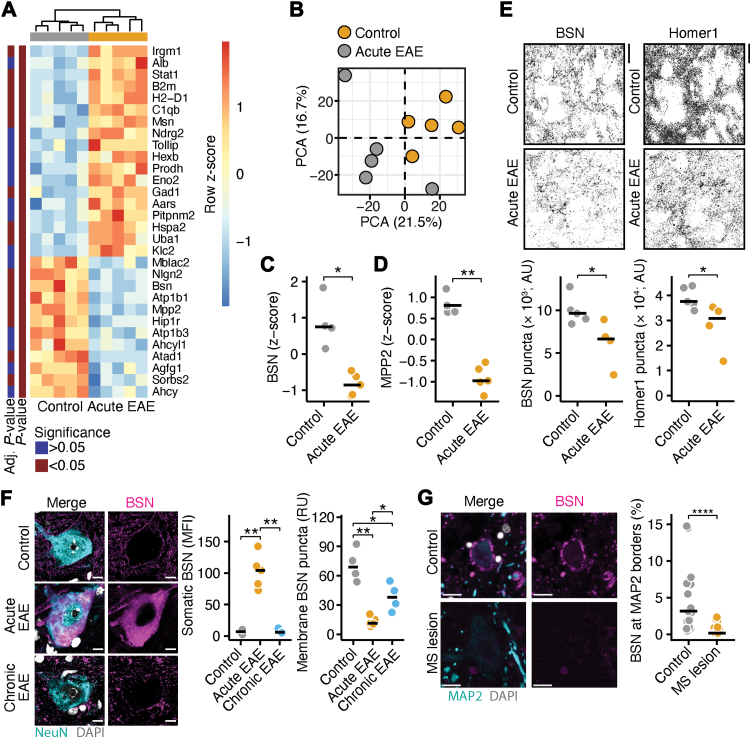
Synaptic BSN is decreased in EAE and MS. **(A and B)** Heatmap of the top 30 regulated proteins (A) and principal component analysis (B) in crude synaptoneurosomes isolated from the cortex of control and acute EAE mice (*n* = 5) measured by label-based mass spectrometry. Row z-scores are shown in the heatmap. Statistical results without multiple-comparison adjustment and with FDR adjustment are annotated in the heatmap. **(C and D)** Z-scores of the presynaptic scaffold protein bassoon (BSN; C) and the postsynaptic scaffold protein palmitoylated membrane protein 2 (MPP2; D) in cortical synaptoneurosomes from control and acute EAE mice (*n* = 5). Statistical results after FDR adjustment for multiple comparisons are shown. **(E)** Quantification of BSN and Homer1 puncta in the cortex of control (*n* = 5) and acute EAE (*n* = 4) mice, shown in absolute units (AU). Scale bar, 20 μm. **(F)** Mean fluorescence intensity (MFI) of somatic BSN and BSN puncta per neuron (RU = relative units) in proximity to the outer membrane of spinal cord neurons in control (*n* = 4), acute EAE (*n* = 5), and chronic EAE mice (*n* = 4). Scale bar, 10 μm. Two-sided *t*-tests were used for statistical comparisons. **(G)** Percentage of BSN at the microtubule associated protein 2 (MAP2) border area in 18 control samples and 19 MS lesions of 4 controls and 4 pwMS. Scale bar, 10 μm. Non-parametric two-sided Mann–Whitney U tests were used for statistical comparisons. ∗*P* < 0.05, ∗∗*P* < 0.01. Abbreviations: BSN = Bassoon, EAE = experimental autoimmune encephalomyelitis, FDR = false discovery rate, MS = multiple sclerosis.

Crude synaptosomal fractions (Supplementary Fig. S1D) were isolated from cortical tissue of control and acute EAE mice and analysed by label-based quantitative mass spectrometry (Fig. 1A and B). This analysis revealed a reduced abundance of the presynaptic protein BSN (Fig. 1C; linear model with Storey’s q-value FDR correction, *P* = 0.048, β-estimate = −0.052), and proteins enriched at excitatory postsynapses like the palmitoylated membrane protein 2 (MPP2; Fig. 1D; linear model with Storey’s q-value FDR correction, *P* = 0.0074, β-estimate = −0.062), and sorbin and SH3 domain containing 2 (SORBS2; Supplementary Fig. S1E; linear model with Storey’s q-value FDR correction, *P* = 0.042, β-estimate = −0.12), and at inhibitory postsynapses like neuroligin-2 (NLGN2; Supplementary Fig. S1F; linear model with Storey’s q-value FDR correction, *P* = 0.034, β-estimate = –0.040). Furthermore, EAE was accompanied by a synaptic upregulation of inflammatory proteins associated with the interferon response, including the signal transducer and activator of transcription 1 (STAT1; linear model with Storey’s q-value FDR correction, *P* = 0.012, β-estimate = 0.62) and the immunity-related GTPase family M member 1 (IRGM1; linear model with Storey’s q-value FDR correction, *P* = 0.0074, β-estimate = 0.94). These findings are consistent with previous studies identifying the neuronal inflammatory stress response as a key driver of EAE pathogenesis.^21^^,^^22^ Additional statistical results are provided in Supplementary Table S1.

We next validated these proteomic findings by immunohistochemistry in cortical sections from acute EAE mice. Staining for the presynaptic marker BSN and the postsynaptic marker Homer1 (Fig. 1E; two-sided Mann–Whitney U test; BSN, *P* = 0.032; Homer1, *P* = 0.032) showed a significant reduction in both proteins, as well as a marked decrease in synaptic density, defined by the spatial overlap of presynaptic and postsynaptic puncta (Supplementary Fig. S1G; two-sided Mann–Whitney U test, *P* = 0.016). These data confirm that both total synapse number and BSN-containing presynapses are reduced in the cortex during EAE.

Notably, our previous work showed that BSN accumulates in the soma of spinal motoneurons during EAE,^20^ contributing to proteostatic stress and neuronal loss. In the present study, we additionally observed that membranous BSN puncta in spinal cord motoneurons were markedly decreased during acute EAE, with partial recovery during the chronic phase (Fig. 1F; two-sided *t*-test; somatic BSN: control vs. acute EAE, *P* = 0.0012; control vs. chronic EAE, *P* = 0.74; acute EAE vs. chronic EAE, *P* = 0.0013; membrane BSN puncta: control vs. acute EAE, *P* = 0.0046; control vs. chronic EAE, *P* = 0.026; acute EAE vs. chronic EAE, *P* = 0.034). Consistent with these findings, post-mortem brain tissue from pwMS also exhibited a significant reduction in BSN-positive presynapses (Fig. 1G; two-sided Mann–Whitney U test, *P* < 0.0001). Together, these results demonstrate that synaptic BSN is reduced, while somatic BSN increased in both EAE and MS, potentially indicating neuronal distress that may lead to BSN release into the CSF.

### BSN is increased in the plasma of acute and chronic EAE mice

**Fig. 2:**
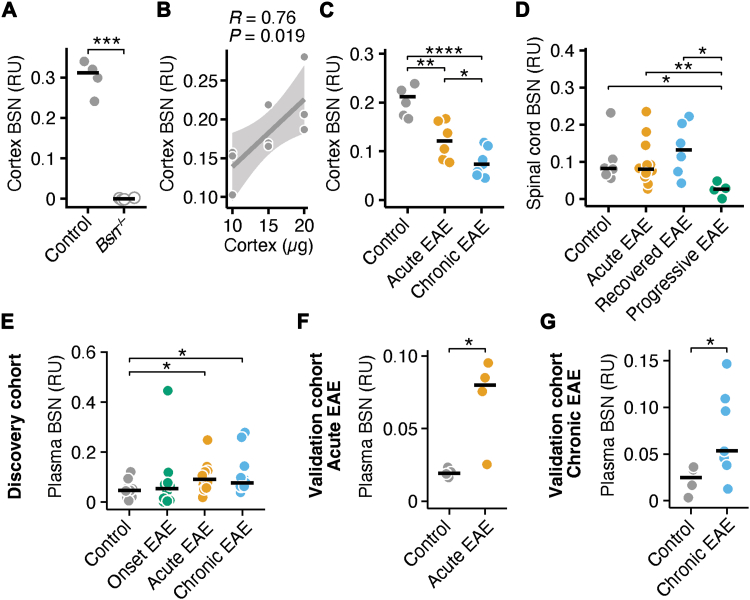
BSN is increased in the plasma of acute and chronic EAE mice. **(A)** BSN measured by ELISA (relative units, RU) in cortex lysates of WT and *Bsn*^*−/−*^ mice (*n* = 4); two-sided *t*-test. BSN measurements in the *Bsn*^*−/−*^ tissues were below background and are shown as 0 in the figure. **(B)** Pearson correlation analysis between BSN levels measured by ELISA and different amounts of cortex lysates (*n* = 3). **(C)** BSN measured by ELISA in cortex lysates from control (*n* = 6), acute EAE (*n* = 6), and chronic EAE (*n* = 7) mice. **(D)** BSN measured by ELISA in spinal cord lysates of control (*n* = 6), acute EAE (*n* = 12), and chronic EAE mice that either recovered (end score <2; recovered EAE; *n* = 6) or did not recover (end score >2; progressive EAE; *n* = 6) while having similar maximal disease scores during the acute EAE phase. **(E)** BSN measured by ELISA in the plasma from control, EAE onset, acute EAE, and chronic EAE mice (*n* = 6 per group). **(F)** BSN measured by ELISA in plasma of an independent cohort of control and acute EAE mice (*n* = 4 per group). **(G)** BSN measured by ELISA in plasma from an independent cohort of control (*n* = 8) and chronic EAE (*n* = 9) mice. Two-sided *t*-tests were used for statistical comparisons. ∗*P* < 0.05, ∗∗*P* < 0.01. Abbreviations: BSN = Bassoon, EAE = experimental autoimmune encephalomyelitis, ELISA = enzyme-linked immunosorbent assay.

To test the hypothesis that BSN levels reflect synaptic pathology, we developed a specific ELISA to quantify BSN in tissues and body fluids. As a first validation step, cortical lysates from wild-type (WT) and *Bsn*-deficient (*Bsn*^*−/−*^) mice were analysed. A strong BSN signal was detected in WT cortices, whereas no signal was observed in *Bsn*^*−/−*^ mice (Fig. 2A; two-sided *t*-test, *P* = 0.0008; median intra-assay coefficient of variation (CV) = 12.1% ± 6.4 interquartile range (IQR)), confirming the assay’s specificity. Serial dilutions of cortical lysates revealed a significant positive correlation between tissue amount and ELISA signal intensity (Fig. 2B; Pearson correlation, *P* = 0.019), indicating quantitative accuracy across a physiological concentration range. To further confirm assay validity, we generated a recombinant BSN peptide containing the epitopes recognised by both the capture and detection antibodies. The ELISA showed a clear concentration-dependent response to the recombinant protein (Supplementary Fig. S2A; Pearson correlation, *P* < 0.0001), providing additional evidence for the assay’s specificity and linearity. Using the recombinant BSN peptide, we estimated assay precision across five independent runs. Intra-assay variability, calculated from triplicate measurements per plate, demonstrated a median coefficient of variation (CV) of 13.8% (IQR 9.3–17.5%) across the full concentration range. Inter-assay precision showed a median CV of 22.5% (IQR 16.0–23.4%) across concentrations. The limit of detection (LOD) and lower limit of quantification (LLOQ), calculated from blank measurements (mean + 3 × SD and mean + 10 × SD), were estimated at 0.14 and 0.26 relative units.

Next, we quantified BSN levels in cortical and spinal tissues of healthy, acute, and chronic EAE mice. In cortical samples, BSN concentrations showed a stepwise decrease from healthy controls to acute and chronic EAE stages (Fig. 2C; two-sided *t*-test; control vs. acute EAE, *P* = 0.0024; control vs. chronic EAE, *P* < 0.0001; control EAE vs. chronic EAE, *P* = 0.045), consistent with progressive synaptic loss. In contrast, spinal cord tissues from acute and chronic EAE mice that exhibited clinical recovery showed no significant change (Fig. 2D; two-sided *t*-test; control vs. progressive EAE, *P* = 0.031; recovered EAE vs. progressive EAE, *P* = 0.012). However, in spinal cords of chronic EAE mice that did not recover after the acute phase, BSN levels were significantly reduced (EAE scores are provided in Supplementary Fig. S2B; two-sided Mann–Whitney U test, recovered EAE vs. progressive EAE; max disease score, *P* = 0.071; disease score last day, *P* = 0.0039).

We then tested whether BSN could be detected in peripheral body fluids. Plasma from a discovery cohort of healthy, acute, and chronic EAE mice revealed significantly increased BSN concentrations during both acute and chronic phases, but not at disease onset (Fig. 2E; two-sided *t*-test; control vs. onset EAE, *P* = 0.45; control vs. acute EAE, *P* = 0.025; control vs. chronic EAE, *P* = 0.035; median intra-assay CV = 23.9% ± 17.1 IQR). These findings were independently confirmed in separate acute (Fig. 2F; two-sided *t*-test, control vs. acute EAE, *P* = 0.045) and chronic EAE (Fig. 2G; two-sided *t*-test, control vs. chronic EAE, *P* = 0.034) cohorts. Together, these results demonstrate that BSN can be quantified by ELISA in tissue and plasma, and that its dynamic changes during EAE mirror cortical synaptic loss.

### BSN levels are increased in the CSF and serum of people with MS

**Fig. 3:**
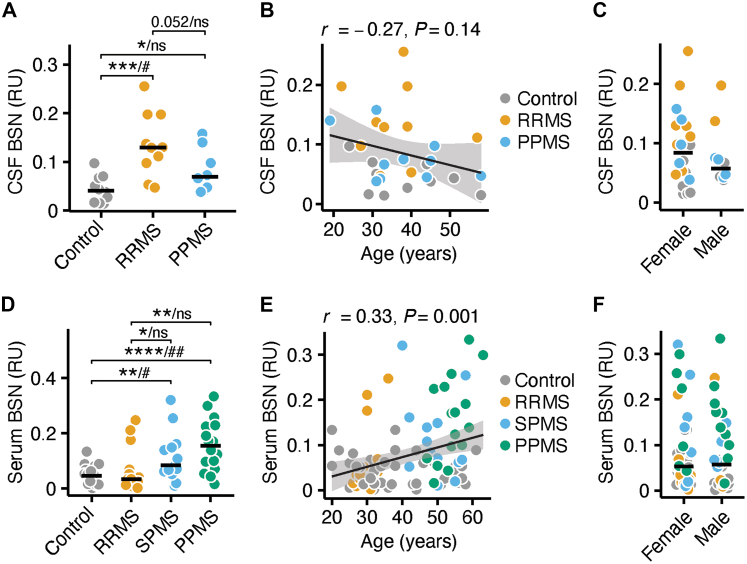
BSN is increased in the CSF and serum of people with MS. **(A)** BSN in the cerebrospinal fluid (CSF; RU = relative units) of controls, people with relapse-remitting MS (RRMS), and primary progressive MS (PPMS; *n* = 10). **(B)** Pearson correlation between age and CSF BSN (*n* = 30). **(C)** CSF BSN comparison between females (*n* = 20) and males (*n* = 10). **(D)** BSN in the serum of controls (*n* = 36), people with RRMS (*n* = 20), secondary progressive MS (SPMS; *n* = 20) and PPMS (*n* = 20). Vaues below the background were set to 0. **(E)** Pearson correlation between age and serum BSN (*n* = 96). **(F)** Serum BSN comparison between females (*n* = 65) and males (*n* = 31). BSN serum values below the detection threshold were set to 0. Non-parametric two-sided Mann–Whitney U tests were used for statistical comparisons. ∗*P* < 0.05, ∗∗*P* < 0.01, ∗∗∗*P* < 0.001, ∗∗∗∗*P* < 0.0001. In (A) and (D), linear models additionally included age, sex at birth, EDSS, disease duration, any immunomodulatory therapy, and high-efficacy disease-modifying treatments as covariates. ^#^*P* < 0.05, ^##^*P* < 0.01. Abbreviations: BSN = Bassoon, CSF = cerebrospinal fluid, EDSS = Expanded Disability Status Scale, MS = multiple sclerosis.

To translate our findings from the EAE model to humans, we next analysed BSN levels in pwMS, reasoning that the protein would first be released into the CSF following synaptic injury. CSF samples were obtained from an age-matched and sex-matched cohort of controls (*n* = 10), people with relapsing-remitting MS (RRMS; *n* = 10), and people with primary progressive MS (PPMS; *n* = 10). BSN was detectable in all samples (Supplementary Fig. S2C). BSN concentrations were significantly higher in the CSF of people with RRMS and PPMS compared with controls (Fig. 3A; two-sided Mann–Whitney U test; control vs. RRMS, *P* = 0.0005; control vs. PPMS, *P* = 0.043; RRMS vs. PPMS, *P* = 0.052) using non-parametric testing, with RRMS patients showing the highest levels. However, CSF BSN levels were only elevated in people with RRMS after adjusting for sex at birth, age, EDSS, disease duration, the use of immunomodulatory therapies, and the use of high-efficacy disease-modifying treatments (linear model; control vs. RRMS, *P* = 0.034; control vs. PPMS, *P* = 0.98; RRMS vs. PPMS, *P* = 0.090; model statistics in Supplementary Table S2). No significant associations with age (Fig. 3B; Pearson correlation, *P* = 0.14) or sex (Fig. 3C; Mann–Whitney U test, *P* = 0.62) were observed (median intra-assay CV = 21.8% ± 20.1 IQR). Clinical information for all included individuals is provided in Table 1.

**Table 1:** Epidemiological data of the cross-sectional cohorts.

Cerebrospinal fluid cohort					
	Control	RRMS	SPMS	PPMS	Student’s *t*-test/ANOVA/Chi-square
N (% female)	10 (70)	10 (80)	NA	10 (50)	*P* = 0.35
Age, years, mean (SD)	38.4 (10.3)	35.8 (9.0)	NA	37.7 (10.3)	*P* = 0.84
EDSS, mean (SD)	NA	1.7 (1.1)	NA	2.9 (0.6)	*P* = 0.0091
Disease duration, years, mean (SD)	NA	0.7 (1.2)	NA	3.3 (3.2)	*P* = 0.0340
Immunotherapy					
Interferon-beta	NA	1	NA	0	

Serum cohort					
	Control	RRMS	SPMS	PPMS	ANOVA/Chi-square
N (% female)	36 (78)	20 (70)	20 (70)	20 (45)	*P* = 0.22
Age, years, mean (SD)	39.75 (11.7)	30.1 (3.7)	49.4 (5.0)	54.2 (5.2)	*P* < 0.0001
EDSS, mean (SD)	NA	1.8 (1.1)	4.8 (1.8)	3.9 (1.5)	*P* < 0.0001
Disease duration, years, mean (SD)	NA	3.3 (4.57)	15.3 (8.9)	9.1 (6.2)	*P* < 0.0001
Immunotherapy					
Natalizumab	NA	1	0	0	
Interferon-beta	NA	0	1	0	
Ocrelizumab	NA	0	0	1	

ANOVA was used for continuous variables and Chi-square test for categorical variables with more than two groups. For continuous variables with two groups, we used Student’s *t*-test.

EDSS = expanded disability status scale, RRMS = relapse-remitting MS, PPMS = primary progressive MS, SPMS = secondary progressive MS.

Because lumbar puncture is an invasive procedure that is not suitable for repeated longitudinal sampling, we next assessed whether BSN could be measured in serum. Serum samples from controls (*n* = 36), RRMS (*n* = 20), secondary progressive MS (SPMS; *n* = 20), and PPMS (*n* = 20) were analysed. Serum BSN (sBSN) levels of 28 out of 96 individuals were below the detection limit (Control, *n* = 12 (33%); RRMS, *n* = 5 (25%); SPMS, *n* = 7 (35%); PPMS, *n* = 4 (20%); Supplementary Fig. S2D) and were set to 0 (Fig. 3D; two-sided Mann–Whitney U test: control vs. RRMS, *P* = 0.92; control vs. SPMS, *P* = 0.0038; control vs. PPMS, *P* < 0.0001; median intra-assay CV = 20.7% ± 21.6 IQR), half of the limit of detection (Supplementary Fig. S2E; two-sided Mann–Whitney U test: control vs. RRMS, *P* = 0.76; control vs. SPMS, *P* = 0.0046; control vs. PPMS, *P* < 0.0001) or were imputed (Supplementary Fig. 2F; two-sided Mann–Whitney U test: control vs. RRMS, *P* = 0.74; control vs. SPMS, *P* = 0.0034; control vs. PPMS, *P* < 0.0001) for subsequent analyses (model statistics are shown in Supplementary Table S3–S5). sBSN levels were significantly elevated in SPMS and PPMS compared with controls, whereas no difference was observed in RRMS across the different analysis methods. These results were confirmed when comparing each MS subtype with sex-matched and age-matched controls (Supplementary Fig. S2G–L; values below LOD set to 0, two-sided Mann–Whitney U test: control vs. RRMS, *P* = 0.60; control vs. SPMS, *P* = 0.010; control vs. PPMS, *P* = 0.0001; the full results with the other imputation methods for values below LOD are shown in Supplementary Fig. S2G–L). Furthermore, sBSN positively correlated with age (Fig. 3E; Pearson correlation, *P* = 0.0014) but showed no sex-related differences (Fig. 3F; two-sided Mann–Whitney U test, *P* = 0.88). Clinical characteristics for all participants are shown in Table 1. Together, these findings indicate that BSN-reported pathology is detectable in early and late MS disease stages.

### sBSN does not change in a clinically stable cohort of PPMS

**Fig. 4:**
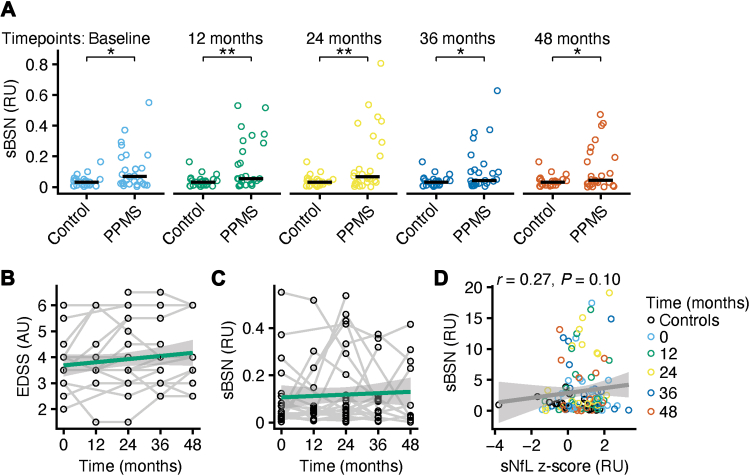
Serum BSN does not change in a primary progressive MS cohort. **(A)** Serum BSN (sBSN; relative units, RU) in controls (*n* = 24) and people with primary progressive MS (PPMS; *n* = 26) at the indicated time points. Linear mixed-effects models including sex at birth, age, Expanded Disability Status Scale (EDSS), and disease duration as covariates were used for statistical comparisons. **(B and C)** Longitudinal trajectories of the EDSS (standardised β = 0.17, *P* < 0.01; B) and sBSN (standardised β = 0.09, *P* = 0.27; C) in our PPMS cohort (*n* = 26). Linear mixed-effects models including age, sex at birth, and baseline values as covariates were used. **(D)** Pearson correlation analysis between serum neurofilament light chain (sNfL) and sBSN (*n* = 26 per PPMS timepoint and 24 controls). Abbreviations: BSN = Bassoon, EDSS = Expanded Disability Status Scale, MS = multiple sclerosis.

To investigate longitudinal changes in sBSN, we analysed a cohort of 26 people with PPMS with an average disease duration of nine years and a mean follow-up period of 37 months.^25^ Matched controls (*n* = 24) were included for comparison. Demographics are shown in Table 2. sBSN was detectable in all samples. Using age-adjusted and sex-adjusted linear models, sBSN levels were significantly higher in people with PPMS compared with controls across all visits (Fig. 4A; linear mixed-effect model; control vs. baseline, *P* = 0.020; control vs. 12 months, *P* = 0.0028; control vs. 24 months, *P* = 0.0013; control vs. 36 months, *P* = 0.015; control vs. 48 months, *P* = 0.015), further supporting elevated sBSN concentrations in people with PPMS.

**Table 2:** Epidemiological data of the longitudinal PPMS cohort.

	Control	PPMS
N (% female)	24 (54)	26 (27)
Age, years, mean (SD)	48.9 (8.9)	53.7 (8.1)
EDSS, mean (SD)	NA	3.7 (1.1)
Disease duration, years, mean (SD)	NA	9.1 (6.0)
Follow-up, months, mean (SD)	NA	35.5 (13.8)
sBSN, RU, mean (SD)	0.04 (0.04)	0.09 (0.13)
sNfL, z-score, mean (SD)	0.22 (1.29)	0.69 (0.99)
Rel. Cortical volume, RU, mean (SD)	NA	0.31 (0.02)
Immunotherapy	NA	0

EDSS = expanded disability status scale, PPMS = primary progressive MS, rel. = relative, RU = relative units, sBSN = serum bassoon, sNfL = serum neurofilament light chain.

Serum NfL (sNfL) levels were numerically higher in people with PPMS without reaching significance (Supplementary Fig. S3A; linear model, *P* = 0.12), suggesting that sBSN and sNfL reflect complementary aspects of MS pathology. We performed cross-sectional correlation analyses of sBSN with different clinical and imaging parameters of MS severity. We did not find any significant correlations of the EDSS (Pearson correlation, *P* = 0.10), cortical volume (Pearson correlation, *P* = 0.52), whole brain volume (Pearson correlation, *P* = 0.95) or cognitive measurements (Pearson correlation; multiple sclerosis inventory for cognition, *P* = 0.69; multiple sclerosis neuropsychological questionnaire, *P* = 0.53) with sBSN (Supplementary Fig. S3B). Next, linear mixed-effects models were applied to assess temporal changes in biomarker levels. Disease progression was evaluated using the EDSS (Fig. 4B) and cortical volume measurements (Supplementary Fig. S3C). EDSS significantly increased over time (linear mixed-effect model, standardised β = 0.17, *P* = 0.0041), whereas cortical volume remained stable (linear mixed-effect model, standardised β = −0.066, *P* = 0.14). Consistent with cortical stability, sBSN levels (linear mixed-effect model, standardised β = 0.091, *P* = 0.27) remained unchanged over time (Fig. 4C). Similarly, sNfL z-scores (linear mixed-effect model, standardised β = −0.081, *P* = 0.29) showed no longitudinal change (Supplementary Fig. S3D). Baseline values of EDSS, sBSN sNfL z-scores and relative cortex volume were significant covariates for the respective longitudinal models highlighting the high between-subject variability of the biomarkers. Full statistical results are provided in Supplementary Table S6. To further characterise the stability of sBSN, we performed variance decomposition using a random-intercept mixed-effects model. The intraclass correlation coefficient was 0.42, indicating that 42% of total variance was attributable to between-subject differences, while 58% reflected within-subject variability over time. The between-person standard deviation (0.11) exceeded the average within-person standard deviation (0.08), suggesting moderate inter-individual stability alongside measurable longitudinal fluctuation. Notably, BSN and NfL levels did not correlate (Fig. 4D; Pearson correlation, *P* = 0.10), supporting the notion that sBSN captures distinct, complementary aspects of neurodegeneration in people with PPMS.

## Discussion

In this study, we provide evidence that synaptic pathology is already detectable in people with RMS and persists continuously throughout progressive disease stages. To investigate this process, we developed a knockout-validated ELISA targeting the presynaptic protein BSN. This assay detected synaptic loss in both the EAE model and in the plasma and CSF of pwMS, offering a translational tool to monitor synaptic pathology across experimental and clinical settings.

Our findings align with recent [^18^F]UCB-H PET studies quantifying synaptic vesicle protein 2A (SV2A) content in RMS and PMS, which correlated with synapses in EAE and postmortem tissues and showed a strong loss.^17^^,^^18^ Consistently, we observed synaptic loss in EAE by proteomics of cortical synaptoneurosomes and immunohistochemistry, and pwMS by detecting a reduction of BSN-positive presynapses. Using our ELISA, we detected a stepwise cortical decrease of total BSN in acute and chronic EAE mice, while spinal cord BSN levels declined significantly only in chronic EAE without recovery after the acute phase. This finding supports persistent cortical pathology in C57BL/6 EAE mice, consistent with the transcriptional alterations we identified in layer 5 cortical motoneurons during acute and chronic disease.^22^

In addition to BSN, we observed the depletion of multiple synaptic scaffold proteins indicating a broad disruption of the synaptic architecture in EAE and MS.^7^^,^^33^ Reduced levels of BSN and the postsynaptic scaffolding proteins MPP2 and SORBS2 are consistent with a loss of excitatory synapses, as these proteins are enriched in glutamatergic synapses. In parallel, the reduction of NLGN2 suggests additional impairment of inhibitory synapses, given its key role as a postsynaptic adhesion molecule at GABAergic contacts. Together, these alterations point toward a disturbance of the excitatory/inhibitory balance, a crucial determinant of cortical network stability that may favour neurodegeneration in MS.^34^^,^^35^ We focused on BSN for further translational analyses because we previously identified its pathological somatic accumulation in neurons in both EAE and MS,^20^ linking it mechanistically to neuronal stress and degeneration.

Along these lines, our synaptoneurosome screen also revealed an upregulation of interferon response proteins like the transcription factor STAT1 and the GTPase IRGM1, further supporting that the neuronal inflammatory stress response is activated in the cortex of C57BL/6 EAE mice. However, the function of these proteins at the synapse is unclear. IRGM1 is an autophagy-related protein and its deficiency in neurons aggravates neuronal death in mouse models of stroke, brain injury and sepsis-induced encephalopathy.^36^, ^37^, ^38^ IRGM1 synaptic localisation in EAE might indicate an inflammation-induced autophagic machinery that regulates synaptic health and removal.^39^ STAT1 is a transcription factor that is localised in the cytoplasm and translocates to the nucleus after phosphorylation.^40^ Although, it has been shown that STAT1 signalling can regulate synaptic plasticity as well as learning and memory,^41^, ^42^, ^43^ the underlying mechanisms are unclear. STAT1 synaptic localisation might indicate a non-canonical function as we have shown for other interferon-induced signalling pathways^21^^,^^22^ and underlines the importance of investigating the subcellular localisation of proteins and pathways in neurons.

BSN also accumulated in the neuronal soma of spinal cord motoneurons in EAE mice, suggesting intraneuronal redistribution rather than overall depletion in the spinal cord. Similar BSN accumulation has been reported in Alzheimer’s disease and its mouse models, where it promotes tau aggregation and propagation.^44^ Moreover, BSN mutations are associated with tauopathies such as the familial and sporadic progressive supranuclear palsy-like syndrome, as well as gait and motor impairment in Parkinson’s disease.^45^, ^46^, ^47^, ^48^ Furthermore, BSN stabilises excitatory synapses, while its deletion or pathogenic gene variants are associated with epilepsy.^49^^,^^50^ The underlying mechanism linking BSN to tauopathy remain unclear, although impairments in axonal transport and proteasomal degradation are likely contributors in MS, Alzheimer’s disease and Parkinsonian syndromes.^21^^,^^51^, ^52^, ^53^, ^54^, ^55^ Elucidating how BSN accumulates in neuronal soma could thus provide broader insight into neuroinflammatory and neurodegenerative processes.

Next, we investigated whether BSN pathology can be assessed *in vivo* by measuring it in body fluids, as shown for other synaptic proteins. We detected elevated BSN levels in the plasma of acute and chronic EAE mice, likely reflecting cortical synaptic loss given the parallel reduction of cortical but not spinal BSN or a general sign of neuronal stress response. Importantly, we also detected BSN in the CSF and serum of pwMS, allowing assessment of BSN pathology in different disease stages. Consistent with SV2A-PET data,^17^^,^^18^ our results indicate enhanced synaptic pathology in people with RRMS, SPMS and PPMS. sBSN levels were higher in people with SPMS and PPMS than in people with RRMS, mirroring the greater cortical synaptic loss observed in SV2A-PET, supporting that sBSN reflects ongoing cortical synapse injury.

Conversely, while CSF BSN levels were elevated in people with RRMS and PPMS compared with controls, sBSN levels were higher in people with RRMS than in people with PPMS. This discrepancy may originate from differences in detected BSN peptides half-lives between matrices, as observed for other biomarkers like GFAP, an astrocytic structural protein and marker of astrogliosis, where plasma GFAP more sensitively detects pathology in Alzheimer’s disease and MS than CSF GFAP.^56^^,^^57^ Further studies analysing paired CSF and sBSN samples and comparing both with SV2A-PET as a reference for synaptic pathology, are needed to determine the most sensitive compartment for peripheral BSN quantification.

To improve assay performances, the BSN ELISA should be transferred to high-sensitivity platforms such as Nucleic Acid Linked Immuno-Sandwich Assay (NULISA)^58^ or Single Molecule Array (SiMOA). While we detected group differences between pwMS and controls, and between RRMS, SPMS and PPMS, approximately 20% of blood measurements fell below the detection limit, unlike CSF samples where all values were measurable. A more sensitive assay will enable finer discrimination of pwMS by sBSN and CSF BSN levels. Notably, we identified a recombinant BSN peptide recognised by the antibodies used in our ELISA, which could serve as positive control for such high-sensitivity platforms. This is crucial because the expression of full-length BSN is technically limited due to its size.

The limited sensitivity of our assay may partly explain the absence of longitudinal changes in our PPMS cohort. Alternatively, BSN stability may reflect relatively preserved cortical volume despite gradual EDSS progression. Longitudinal studies are warranted to assess dynamic BSN changes within body fluids across RRMS, SPMS and PPMS disease stages. Interestingly, we observed no correlation between sBSN and sNfL. In our PPMS cohort, sNfL concentrations were numerically elevated without reaching statistical significance and did not change over time, consistent with the notion that NfL mainly reflects acute inflammatory axonal injury rather than chronic neurodegeneration.^13^^,^^14^ Nonetheless, NfL levels increase during RMS remission and predict disability worsening associated with both relapsing and non-relapsing progression.^13^^,^^14^^,^^59^ In contrast, GFAP is more closely associated with disease progression independent of relapse activity in both RMS and PMS. Collectively, these findings suggest that BSN captures complementary aspects of synaptic pathology distinct from NfL and GFAP, with potential utility for predicting neurodegeneration.^14^^,^^16^

Our study has several limitations. Although we established and validated a knockout-controlled ELISA for BSN quantification, its sensitivity limits detection at low concentrations, and absolute concentrations could not be calculated. This is likely due to the low endogenous abundance of circulating BSN in serum and CSF samples. Therefore, we report relative units (background-subtracted signal), which limits direct cross-cohort comparability and the determination of cut-offs. To enhance reproducibility across runs and plates, all samples were processed under identical assay conditions, standardised blank correction was applied, a recombinant BSN peptide dilution series ranging from 0.1 ng mL^−1^ to 10 ng mL^−1^ was included on each plate, and inter-assay precision was quantified across independent runs. Future iterations of the assay incorporating a full-length recombinant BSN calibrator or orthogonal high-sensitivity platforms quantitative platforms will be required to enable absolute quantification and facilitate inter-laboratory transferability. Moreover, although both mouse and human data indicate cortical synapse and BSN loss, it remains unclear whether sBSN quantitatively reflects synaptic density. Correlation of BSN concentrations with synaptic PET imaging will be essential to resolve this. The absence of longitudinal sBSN changes in people with PPMS may relate to stable cortical volume and neuroaxonal integrity. This may also be explained by the small sample size of the study that limits the statistical power of our analyses. Additional longitudinal and multicentre studies are needed to evaluate whether BSN predicts progression associated with relapses or progression independent of relapse activity and to determine its dynamics during acute relapses. Although we detected increased BSN in the CSF and serum of pwMS, we did not measure BSN in both matrices in the same individuals. Furthermore, disease duration and EDSS varied substantially between our serum and CSF cohorts. Therefore, our study does not allow direct comparison of BSN levels between serum and CSF. Future studies should evaluate the correlation between CSF and serum BSN within the same individuals, which would have important implications for interpretation. Finally, while BSN is pathophysiologically linked to disease progression in preclinical MS models and thus represents a promising biomarker, we did not compare it with other established synaptic proteins. Several other synaptic proteins were also decreased in our synaptoneurosome screen indicating that also other proteins might be suitable to measure synaptic loss in MS. Integrating BSN into composite synaptic biomarker panels, similar to approaches in Alzheimer’s disease,^30^ could refine biomarker-based assessment of neuronal pathology in MS and deepen our understanding of BSN pathology in MS.

In summary, this study demonstrates that synaptic pathology is continuously detectable throughout early and later MS stages, underscoring the need for early neuroprotective therapeutic strategies targeting synaptic integrity.

## Contributors

MAF, MSW, NR designed and conceptualised the study. MSW and NR performed most experiments and analyses. MAF, MSW, NR accessed and verified the underlying data. MSW, LCB, CM, IW, PS analysed the clinical data. IW, AW coordinated biobanking and advised cohort selection. LCB, LR, SB, BS, JBE performed histological analyses in mice. MK, DM performed histological analyses of human tissues. AMM, JK performed sNfL measurements. CK, BS, HS performed proteomics of synaptoneurosomes. SW produced the BSN peptide. SCR and JPS recruited the PPMS cohort. MSW, LCB, MAF wrote the first version of the manuscript. MAF funded and supervised the study. All authors reviewed the manuscript and agreed to the final version of the manuscript.

## Data sharing statement

The data supporting the findings of this study and the protocols are included in the article and Supplementary data. Further enquiries regarding raw data and the code used for analyses and visualisations can be directed to the corresponding author.

## Declaration of interests

MAF received funding from the Hamburg Innovation Call for Transfer (C4T959), and Deutsche Forschungsgemeinschaft (FOR 5705, 523862973), received consulting fees from Alexion, Kyverna, Lundbeck, Merck KGaA, Novartis, Roche, Sudo Biosciences, Gemeinnützige Hertie-Stiftung, Wings for Life and has stocks in Bayer and NovoNordisk outside the scope of this manuscript. MK acted as cash auditor for SDiPATH outside the scope of this manuscript. SCR received financial support of the Bundesministerium für Forschung, Technologie und Raumfahrt, Gemeinnützige Hertie-Stiftung, and Deutsche Forschungsgemeinschaft, received honoraria from Roche, Sanofi, Merck KGaA, Florian Schmitz Kommunikation, support for travel from Sanofi and has a fiduciary role in the German Multiple Sclerosis Society outside the scope of this manuscript. All other authors declare no competing interests.
