# Supplementary material for: The presynaptic protein bassoon is a biofluid biomarker of synaptic pathology in multiple sclerosis
Source: eBioMedicine. 2026 May 7;128:106282. doi: 10.1016/j.ebiom.2026.106282 (PMC13156686; doi:10.1016/j.ebiom.2026.106282)
Supplement: Supplementary Figs. S1–S3 and Tables S1–S6 [file mmc1.pdf]

SUPPLEMENTARY MATERIAL

The presynaptic protein Bassoon is a biofluid biomarker of synaptic pathology in multiple sclerosis

AUTHORS

Marcel S. Woo[1,*], Nicola Rothammer[1,*], Lukas C. Bal[1], Christoph Krisp[2], Mario Kreutzfeldt[3,4], Susanne Witt[5], Aleksandra Maleska Maceski[6], Bente Siebels[2], Lukas Raich[1], Christina Mayer[1], Ingo Winschel[1], Anne Willing[1], Benjamin Schattling[1], Simone Bauer[1], Hartmut Schlüter[2], Sina C. Rosenkranz[1], Jens Kuhle[6], Jan-Patrick Stellmann[1,7,8], Jan Broder Engler[1], Doron Merkler[3,4], Manuel A. Friese[1,§]

AFFILIATIONS

[1]Institute of Neuroimmunology and Multiple Sclerosis (INIMS), University Medical Center Hamburg-Eppendorf, Hamburg, Germany

[2]Section of Mass Spectrometry and Proteomics, University Medical Center Hamburg-Eppendorf, Germany

[3]Division of Clinical Pathology, Geneva University Hospital, Geneva, Switzerland

[4]Department of Pathology and Immunology, University of Geneva, Geneva, Switzerland

[5]Protein Production (PP) Core Facility, University Medical Center Hamburg-Eppendorf (UKE), Hamburg, Germany

[6]MS Center, Neurology and Research Center for Clinical Neuroimmunology and Neuroscience Basel, Departments of Clinical Research and Biomedicine, University Hospital and University Basel, Basel, Switzerland

[7]CEMEREM, APHM, Hôpital de la Timone, Marseille, France

[8]CRMBM, Aix Marseille University, CNRS, Marseille, France

*These authors contributed equally

[§]**Corresponding author**: Manuel A. Friese, Institute of Neuroimmunology and Multiple Sclerosis (INIMS), University Medical Centre Hamburg-Eppendorf, Falkenried 94, D-20251 Hamburg, Germany; Email: manuel.friese@zmnh.uni-hamburg.de

TABLE OF CONTENTS

Supplementary Tables

- Table S1. Statistical results of the synaptoneurosome proteomics data.
- Table S2. Statistical results of linear model with CSF BSN.
- Table S3. Statistical results of linear model with serum BSN where values below background were replaced with zero.
- Table S4. Statistical results of linear model with serum BSN where values below background were replaced with half of the limit of detection.
- Table S5. Statistical results of linear model with serum BSN where values below background were imputed.
- Table S6. Statistical results of longitudinal models.

Supplementary Figures

- Fig. S1. Cortical pathology in C57BL/6 mice.
- Fig. S2. A recombinant BSN peptide can be detected by ELISA.
- Fig. S3. Serum neurofilament light chain does not change in a primary progressive MS cohort.

SUPPLEMENTARY FIGURES

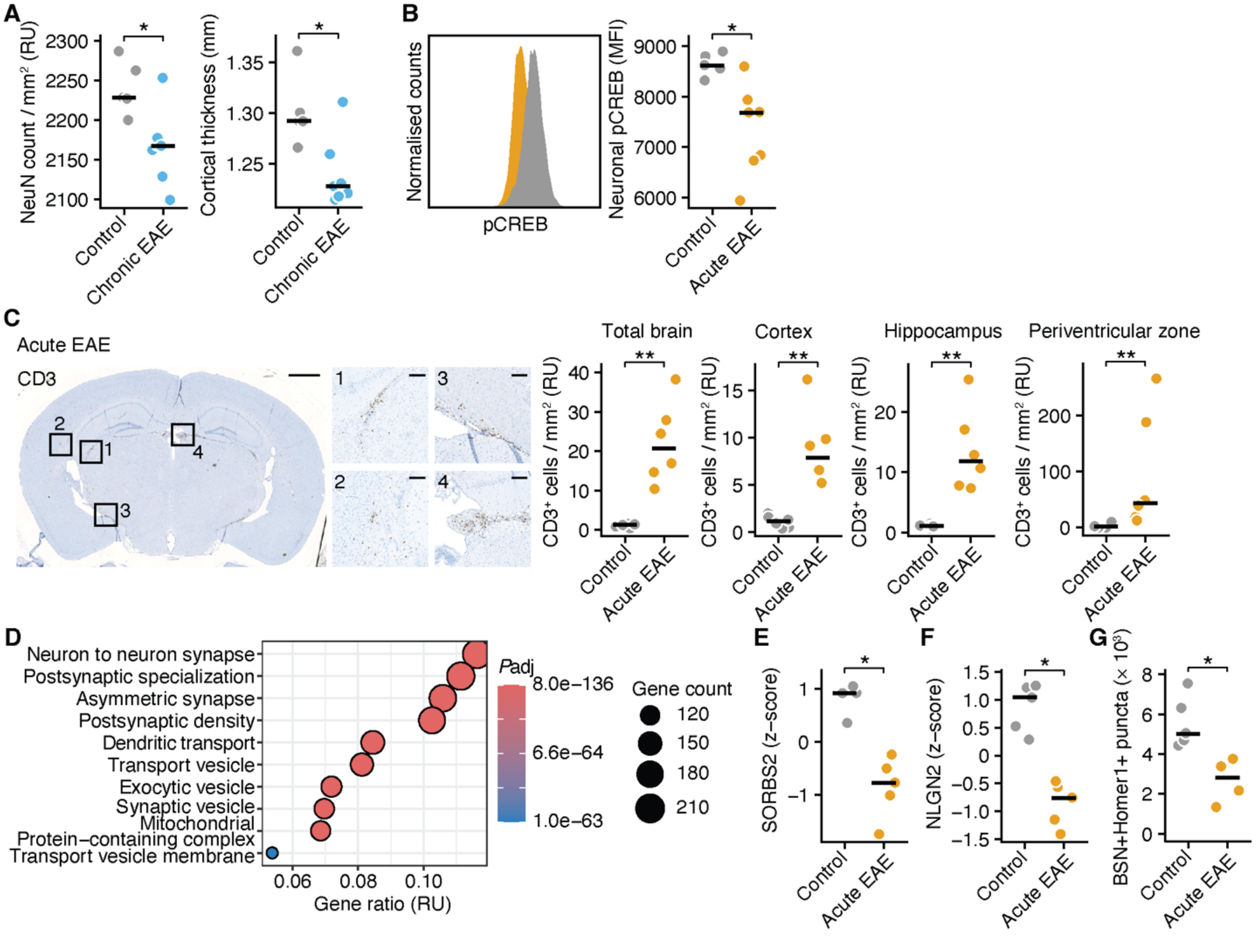

Fig. S1: Cortical pathology in C57BL/6 mice. (A) Neuronal counts per mm^2 (relative units, RU) and cortical thickness (mm) in control (n = 5) and chronic EAE (n = 7) mice. (B) Neuronal pCREB measured by flow cytometry in the cortex of control (n = 5) and acute EAE (n = 7) mice. (C) CD3+ cells per mm^2 in control mice and acute EAE mice in total brain, cortex, hippocampus and periventricular zone (n = 6 per group). Scale bar in overview image = 1 mm, scale bar in magnifications = 200 µm. (D) Top 10 Gene Ontology (GO) cellular component terms of all proteins detected in the crude cortical synaptoneurosomes. The gene ratio between the detected genes and genes in the respective GO term is shown in relative units (RU). FDR correction was applied to account for multiple comparisons. (E-F) Z-scores of postsynaptic scaffold proteins sorbin and SH3 domain-containing protein 2 (SORBS2; E) and neurologin-2 (NLGN2; F) abundance in cortical synaptoneurosomes from control and acute EAE mice (n = 5). Statistical results after FDR adjustment for multiple comparisons are shown. (G) Number of Bassoon (Bsn)$^+$ Homer1$^+$ puncta in the cortex of control (n = 5) and acute EAE (n = 4) mice. Non-parametric two-sided Mann-Whitney U tests were used for statistical comparisons. * $P < 0.05$, ** $P < 0.01$. Abbreviations: CD = cluster of differentiation, EAE = experimental autoimmune encephalomyelitis, MFI = mean fluorescence intensity, pCREB = phosphorylated cAMP responsive element binding protein 1.

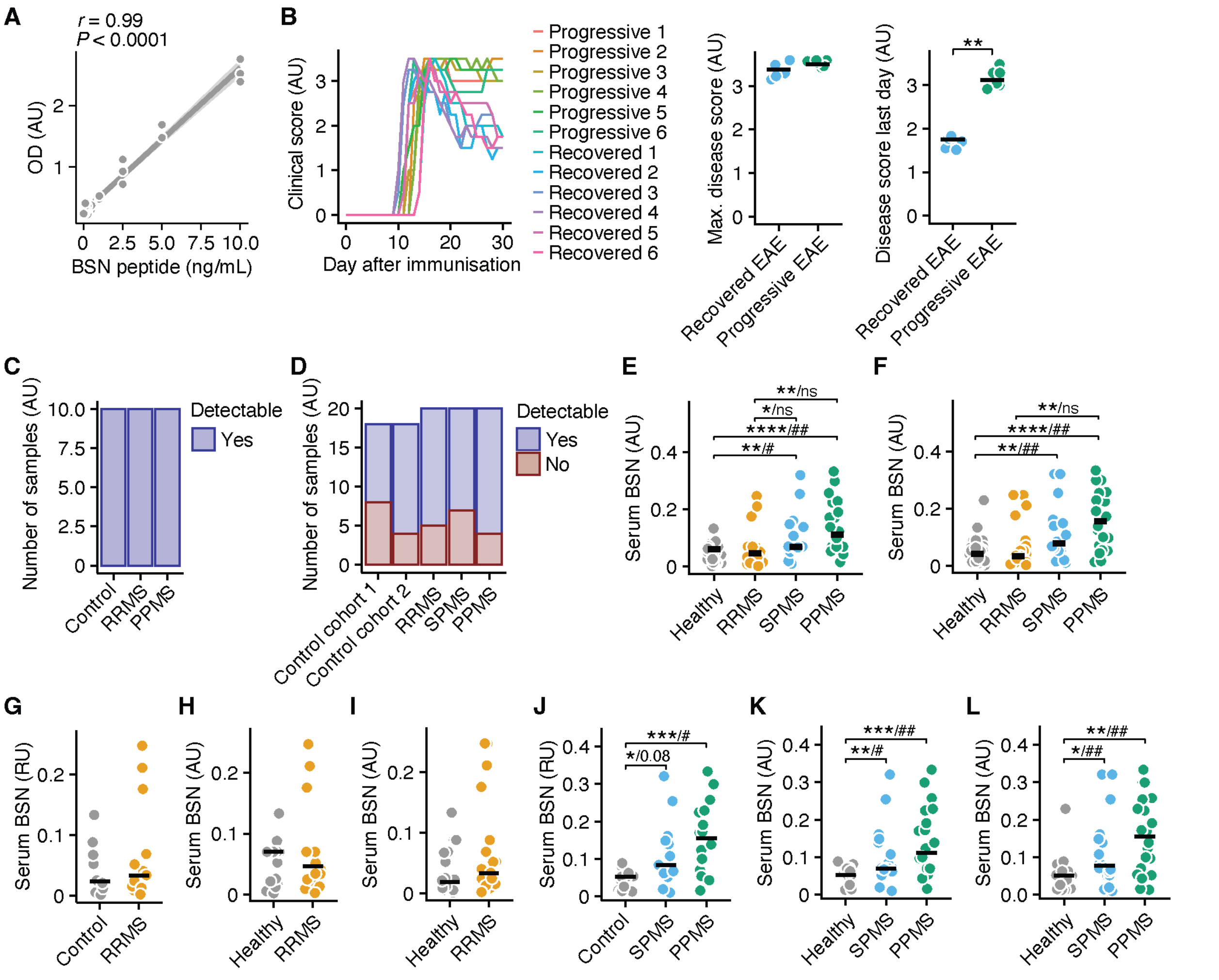

Fig. S2: A recombinant BSN peptide can be detected by ELISA. (A) Optical density (absolute units, AU) of a recombinant BSN peptide measured by ELISA (*n* = 3 per concentration). The BSN peptide included the epitopes of both antibodies. Pearson correlation was calculated. (B) Individual disease curves, maximal disease score and disease score at the last day in AU in recovered and progressive chronic EAE mice (*n* = 6). (C,D) Number of CSF (C) and serum (D) samples in which BSN was detectable or undetectable. (E,F) BSN in serum of controls (*n* = 36), people with RRMS (*n* = 20), SPMS (*n* = 20), and PPMS (*n* = 20). Vaues below the background were set to half of the limit of detection (LOD; E) or imputed (F). (G-I) Serum BSN in age-matched controls (*n* = 18) and people RRMS (n = 20). Serum BSN values below the detection threshold were set to zero (G), half of the LOD (H) or were imputed (I). (J-L) Serum BSN in age-matched controls (*n* = 18) and people with SPMS (*n* = 20) and PPMS (*n* = 20). Serum BSN values below the detection threshold were set to 0 (J), half of the LOD (K), or imputed (L). Non-parametric two-sided Mann-Whitney U tests were used for statistical comparisons. * $P < 0.05$, ** $P < 0.01$, *** $P < 0.001$, **** $P < 0.0001$. In (E-L), linear models additionally included age, sex at birth, EDSS, disease duration, any immunomodulatory therapy, and high-efficacy disease-modifying treatments as covariates. $^{\#}$ $P < 0.05$, $^{\#\#}$ $P < 0.01$. Abbreviations: BSN = Bassoon, EAE = experimental autoimmune encephalomyelitis, EDSS = Expanded Disability Status Scale, ELISA = enzyme-linked immunosorbent assay, LOD = limit of detection, MS = multiple sclerosis.

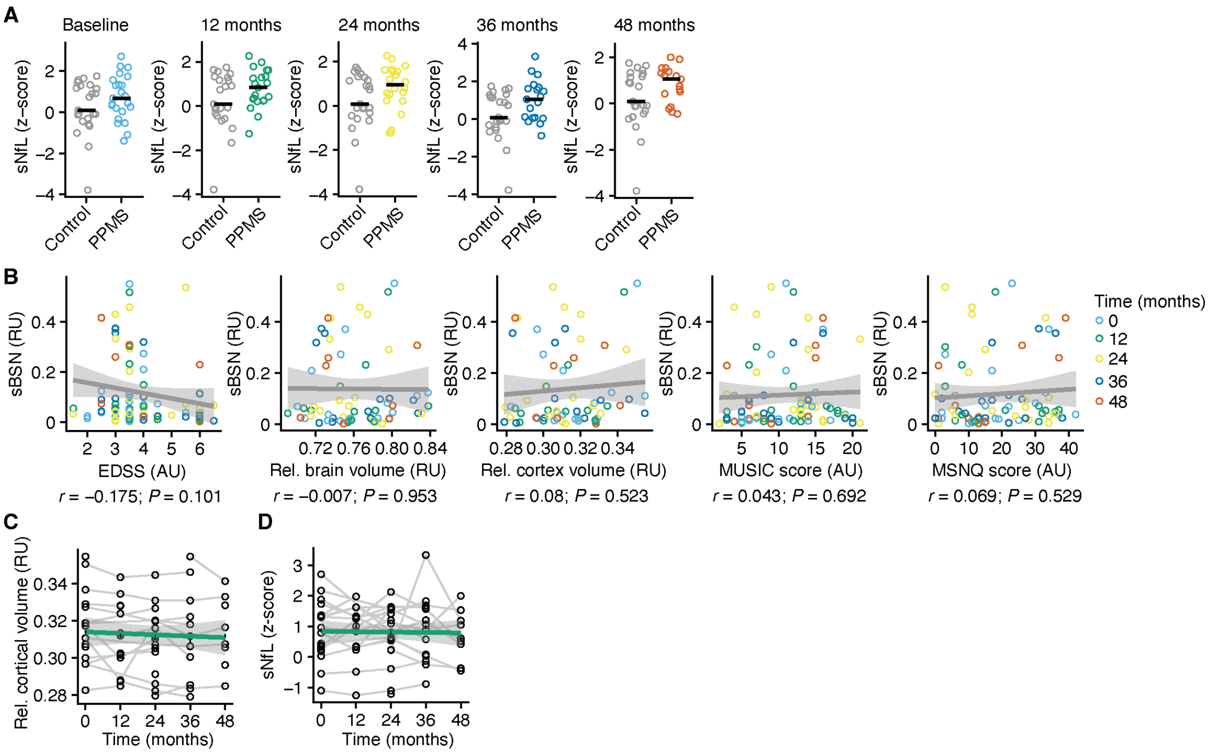

Fig. S3. Serum neurofilament light chain does not change in a primary progressive MS cohort. (A) Serum neurofilament light chain (sNfL) age-adjusted z-scores in controls (n = 24) and people with PPMS (n = 26) at the indicated time points. Linear mixed-effects models including sex at birth, age, EDSS, and disease duration as covariates were used for statistical comparisons. (B) Pearson correlation analysis of sBSN in relative units (RU) with the EDSS, relative brain volume normalised to the total intracranial volume, relative cortex volume normalised to total intracranial volume, the Multiple Sclerosis Inventory for Cognition (MUSIC) score, and the Multiple Sclerosis Neuropsychological Questionnaire Score (MSNQ) score across all time points (n = 26 individuals across five time points). (C,D) Longitudinal trajectories of relative cortical volume normalised to the total intracranial volume (standardised β = –0.07, P = 0.14; C) and sNfL (standardised β = –0.08, P = 0.29; D) in our PPMS cohort (n = 26). Linear mixed-effects models including age, sex at birth, and the respective baseline values were used as covariates. Abbreviations: BSN = Bassoon, EDSS = Expanded Disability Status Scale, MS = multiple sclerosis.

SUPPLEMENTARY TABLES

PROTEIN	BETA-ESTIMATE	STANDARD ERROR	*P* VALUE	ADJUSTED *P* VALUE
Irgm1	0.94425744	0.092717335	7.4053E-06	0.007434653
Mpp2	-0.0620436	0.006424817	1.1009E-05	0.007434653
B2m	0.61314581	0.071593589	2.6651E-05	0.011998187
Stat1	0.6241374	0.076115352	3.6545E-05	0.01233953
C1qb	0.31227246	0.042188868	7.6066E-05	0.02054719
Atp1b1	-0.0600836	0.009322179	0.00019932	0.033649847
Gad1	0.1038664	0.015924958	0.00018375	0.033649847
Nlgn2	-0.0398071	0.006077914	0.00017857	0.033649847
Hspa2	0.0830484	0.013177782	0.00023225	0.034852998
Sorbs2	-0.1248556	0.02096966	0.00034041	0.041796348
Uba1	0.0708804	0.011872003	0.00033426	0.041796348
Atad1	-0.0908628	0.015963555	0.00045865	0.048338685
H2-D1	0.52496038	0.092431079	0.00046527	0.048338685
Bsn	-0.0552392	0.010006647	0.00056022	0.048622424
Hip1r	-0.0704149	0.012810474	0.000576	0.048622424
Msn	0.1899387	0.034367389	0.00055597	0.048622424
Ahcy	-0.1280612	0.024518397	0.0007996	0.059997047
Klc2	0.05084848	0.009694349	0.00077839	0.059997047
Agfg1	-0.1032837	0.02051371	0.00100804	0.061686384
Atp1b3	-0.0809542	0.015723185	0.0008757	0.061686384
Hexb	0.1392738	0.027844795	0.00105048	0.061686384
Prodh	0.11251575	0.022405779	0.00102467	0.061686384
Tollip	0.14892714	0.029365473	0.00096321	0.061686384
Mblac2	-0.0353326	0.00713736	0.00112036	0.063048775
Ndrg2	0.1872706	0.039133465	0.00138103	0.07460953
Aars	0.0962578	0.020794792	0.00169039	0.084557837
Pitpnm2	0.0870984	0.018760875	0.00166068	0.084557837
Ahcyl1	-0.0826788	0.018623144	0.00216918	0.10356957
Eno2	0.1064698	0.024083276	0.00222382	0.10356957
Alb	0.8710794	0.19833349	0.00231137	0.104059117
Gnao1	-0.0243146	0.005645998	0.00259282	0.110093339
Syt1	-0.061117	0.014206403	0.00260843	0.110093339
Atp5i	-0.0814852	0.019049648	0.00269656	0.110364083
Pclo	-0.054816	0.012884082	0.00278189	0.110507819
Begain	-0.0542716	0.013604744	0.00400998	0.123743134
Cdc42	-0.0571138	0.01433084	0.00403128	0.123743134
Cryab	-0.172489	0.042867209	0.00382089	0.123743134
Dlgap1	-0.062773	0.015293855	0.00341666	0.123743134
Grin2a	-0.1045152	0.025813585	0.00369013	0.123743134
Ndufv1	-0.0526878	0.013150942	0.00391472	0.123743134
Pkp4	-0.053114	0.013285379	0.00396121	0.123743134
Rap1b	-0.0186668	0.004576741	0.00354089	0.123743134
Setd7	0.11543064	0.028875079	0.0039631	0.123743134
Syn3	0.0696776	0.017148627	0.00361756	0.123743134
Atp5o	-0.061925	0.015744978	0.00433835	0.127986978
Dvl1	-0.1563356	0.039927966	0.00444684	0.127986978
Gsk3b	-0.0583199	0.014899078	0.00445382	0.127986978
Cacnb4	-0.0550666	0.0142388	0.00475907	0.131176926
Phb2	-0.0514332	0.013298094	0.00475672	0.131176926
Actr3	-0.0294336	0.00770442	0.00508717	0.132591146
Gm996	-0.0502082	0.013124008	0.00504883	0.132591146
Nars	0.10438928	0.027438227	0.00520306	0.132591146
Qdpr	-0.1032641	0.027055929	0.00511372	0.132591146
Itsn1	-0.0658941	0.017433057	0.0053894	0.132754732
Ppap2b	-0.144401	0.038479372	0.00560265	0.132754732
Rab6a	-0.0498292	0.013257896	0.0055566	0.132754732
Tspan7	-0.1245006	0.033159284	0.00558714	0.132754732
Dpysl3	0.0658504	0.017717576	0.00589953	0.13505077
Ppm1e	0.08794131	0.023659325	0.00589689	0.13505077
Slc9a7	-0.0646488	0.01748417	0.00606384	0.136498494
Gabbr1	-0.0762862	0.020743107	0.00624011	0.138163687

Cacng2	-0.0978202	0.026711439	0.00638176	0.139020829
Grm3	-0.0681828	0.018933443	0.00697031	0.142639385
Psmd11	0.03762201	0.010386186	0.0067599	0.142639385
Ubl3	-0.1098862	0.030255238	0.00666596	0.142639385
Uqcrc2	-0.0639978	0.017748988	0.0069246	0.142639385
Scg5	0.12719478	0.035515945	0.00717424	0.144621363
Nrxn1	-0.0610896	0.017117031	0.00730498	0.145091261
Pld3	-0.134846	0.037982139	0.00750673	0.146937579
Zadh2	-0.0713492	0.020311206	0.00792902	0.152986343
Itm2c	0.1025872	0.029286508	0.00804503	0.153038397
Cftr	-0.0661542	0.019065294	0.00844426	0.153842116
Kctd16	-0.0550622	0.015812092	0.00829179	0.153842116
Slc25a25	-0.0547444	0.015758424	0.00839355	0.153842116
Ugp2	-0.1069626	0.030896382	0.0085429	0.153842116
Immt	-0.0495222	0.014388613	0.00880091	0.15437195
Rab10	-0.040415	0.011729956	0.00875322	0.15437195
Hint1	0.0558394	0.016273731	0.00893822	0.154770424
Atp2a2	-0.0543872	0.01616957	0.00987958	0.15754673
Atp5a1	-0.061629	0.018542898	0.0104839	0.15754673
Atp5c1	-0.0637692	0.019041227	0.01009518	0.15754673
Chmp4b	0.109803	0.032698957	0.00996135	0.15754673
Gfap	0.1729248	0.051667672	0.01012732	0.15754673
Grin1	-0.0655134	0.019600651	0.01019459	0.15754673
Hapln1	0.064322	0.019368024	0.01052376	0.15754673
Idh3g	-0.055355	0.016707042	0.01064617	0.15754673
Limd2	0.1212097	0.035841785	0.009616	0.15754673
Nol3	0.0926552	0.027509153	0.00981233	0.15754673
Ppp1r7	0.1032567	0.030888687	0.0101877	0.15754673
Scai	-0.056226	0.016926086	0.01051102	0.15754673
Snx2	0.07224508	0.021259782	0.00938536	0.15754673
Uqcrc1	-0.067062	0.020273246	0.01073163	0.15754673
Atp6v0a1	-0.046325	0.014096029	0.01108164	0.1575476
Ctnna2	-0.0390508	0.011873869	0.01104177	0.1575476
Slc25a3	-0.0868246	0.026316402	0.01087087	0.1575476
Letm1	-0.0677986	0.020699069	0.01126424	0.158475456
Cadm4	-0.0828776	0.025424497	0.01153154	0.160563474
Bcan	-0.1248946	0.038503112	0.01181102	0.162776856
Pcsk1n	0.2292782	0.070854394	0.01195071	0.163038356
Rab6b	-0.0688682	0.021340572	0.01210952	0.163552853
Opa1	-0.0462294	0.014434941	0.0125631	0.167998975
Aldoc	0.1090428	0.034588385	0.01354557	0.170963385
Arl6ip1	0.09338752	0.029679983	0.01367085	0.170963385
Ctnnd2	-0.0356174	0.011217897	0.01309466	0.170963385
Dlg4	-0.0588196	0.018585273	0.01329742	0.170963385
Eea1	-0.0809416	0.025529534	0.01318455	0.170963385
Srcin1	-0.0564214	0.017896722	0.01354503	0.170963385
Tomm5	-0.1210985	0.038225402	0.01323424	0.170963385
Slc3a2	-0.0715142	0.022925459	0.01424111	0.176460957
Plcl2	-0.1468126	0.047186307	0.01441626	0.177007371
Arhgef2	-0.0592284	0.019132803	0.01476275	0.179628629
Atp1a1	-0.0814242	0.026473385	0.01521579	0.18186424
Picalm	-0.078546	0.025507437	0.01513208	0.18186424
Fam177a1	-0.110925	0.036615743	0.01632345	0.185411932
Gk	-0.0861824	0.028452523	0.0163345	0.185411932
Mrps25	-0.2007272	0.066009279	0.01604148	0.185411932
Nrcam	-0.076468	0.025044359	0.01574188	0.185411932
Shank2	-0.0515058	0.017004687	0.01633628	0.185411932
Syngr3	-0.0694748	0.022774983	0.01580932	0.185411932
Adam23	-0.0932808	0.030874341	0.01652662	0.186009109
Clcn4	-0.0713558	0.023663574	0.01667514	0.186129656
Cul2	0.1733888	0.058272797	0.0177226	0.194604809
Sept5	-0.0340028	0.011419578	0.01766532	0.194604809
Atp5h	-0.06746	0.022904757	0.0185603	0.198981162
Exog	-0.0591179	0.020073042	0.01856313	0.198981162
Samm50	-0.048954	0.016600498	0.01845536	0.198981162
Akr1a1	0.07468162	0.02559769	0.01936487	0.201188254

Ndufa10	-0.0681352	0.023276179	0.01907829	0.201188254
Plec	-0.052974	0.01814699	0.01931606	0.201188254
Slc4a10	-0.1129511	0.038607138	0.01912497	0.201188254
P4hb	0.0569434	0.019575428	0.01962148	0.202298146
Atp6v0d1	-0.0403666	0.01401669	0.02051483	0.203059918
Gabra1	-0.0734286	0.025413315	0.02021853	0.203059918
Mgrn1;Rnf157	-0.1347024	0.046477508	0.01994584	0.203059918
Ntrk2	-0.0621255	0.021589102	0.02058617	0.203059918
Rac1;Rac2;Rac3	-0.0440374	0.015323901	0.02070861	0.203059918
Rims2	-0.1140506	0.039606817	0.02052508	0.203059918
Rnpep	0.09764696	0.033993232	0.02074779	0.203059918
Pgam1	0.086573	0.030378979	0.02148677	0.208525691
Pisd	-0.1354062	0.047579465	0.02161505	0.208525691
Iqsec2	-0.0510806	0.018007843	0.0219272	0.208557712
Tpi1	0.0897488	0.031611821	0.02184253	0.208557712
Actr2	-0.0441456	0.015694157	0.02274233	0.208632245
Cox4i1	-0.05498	0.019604479	0.02303887	0.208632245
Lrrc7	-0.0454002	0.016225551	0.02326703	0.208632245
Mat2a	0.094076	0.033567593	0.02310562	0.208632245
Nck2	-0.095723	0.033954647	0.02252359	0.208632245
Ndufa4	-0.0830828	0.029537504	0.02274508	0.208632245
Pdia3	0.0509812	0.018147843	0.02287069	0.208632245
Psma6	0.0550132	0.01967254	0.02332529	0.208632245
Slc8a1	-0.0434868	0.015387842	0.02228533	0.208632245
Vdac2	-0.0669242	0.024066362	0.02389434	0.212316031
Adcy9	0.03387	0.012261919	0.02459069	0.213438101
Ptk2b	0.14922994	0.054126751	0.02478762	0.213438101
Rdx	-0.0622663	0.022609746	0.0249061	0.213438101
Slc7a8	-0.2067057	0.075167389	0.0250613	0.213438101
Ttll13	-0.0761396	0.02762365	0.02481546	0.213438101
Uqcrfs1	-0.071371	0.025969629	0.02512683	0.213438101
Vwa5a	0.119105	0.043013428	0.02433351	0.213438101
Pdxp	0.0512502	0.01869446	0.02539168	0.214339814
Apoo	-0.062339	0.022916882	0.02623972	0.214786294
Camk2a	0.0476126	0.01745242	0.02592036	0.214786294
Dlg2	-0.046021	0.016894238	0.02608416	0.214786294
Gria2	-0.0396026	0.014536023	0.02606882	0.214786294
Phb	-0.0349894	0.012817004	0.02584898	0.214786294
Grm2	-0.0488375	0.01800442	0.02655407	0.214960661
Slc25a5	-0.0672968	0.024815263	0.02657933	0.214960661
Epha4	-0.0776206	0.028836974	0.02742415	0.218663753
Exoc2	-0.0865311	0.032195103	0.02759436	0.218663753
Ndufb10	-0.0592318	0.02205535	0.02768481	0.218663753
Ndufs3	-0.0573486	0.021265536	0.02720913	0.218663753
Ak1	0.099923	0.037423954	0.02836102	0.221415055
Hprt1	0.08930302	0.033412297	0.02824119	0.221415055
Comtd1	-0.0537786	0.02024094	0.02894333	0.221642774
Ermn	-0.1128452	0.042548853	0.02915912	0.221642774
Gabrb2	-0.0838965	0.031621117	0.02911184	0.221642774
Mpp3	-0.0552341	0.020785839	0.02892664	0.221642774
Vps51	0.06404192	0.024157701	0.02921072	0.221642774
Amph	0.0673404	0.026114333	0.03268355	0.225095886
Atp5l	-0.0800724	0.030464632	0.03025448	0.225095886
Atpif1	-0.0450178	0.01751843	0.03314099	0.225095886
Cacna1b	-0.0355981	0.013540107	0.0302212	0.225095886
Calb2	0.1481129	0.05708264	0.03187884	0.225095886
Cuta	-0.070093	0.027280177	0.0331598	0.225095886
Gabrg2	-0.0966554	0.037316426	0.0321053	0.225095886
Gnb2	-0.0333706	0.012677407	0.03007113	0.225095886
Kcnma1	-0.045375	0.017624784	0.03289646	0.225095886
Lingo1	-0.0637394	0.024755135	0.03288141	0.225095886
Nptn	-0.0598286	0.02324074	0.03290682	0.225095886
Plxna1	-0.0377602	0.014696915	0.03316571	0.225095886
Prep	0.0797468	0.0306736	0.03162512	0.225095886
Rap1a	-0.0578696	0.022407067	0.03248231	0.225095886
Scg2	0.1208732	0.046292657	0.03107877	0.225095886

Sec13	0.1635408	0.063174738	0.03217779	0.225095886
Slc4a3	-0.0869334	0.033685802	0.03258016	0.225095886
Slc4a4	-0.048963	0.01899552	0.03273789	0.225095886
Syngap1	-0.0384992	0.014971454	0.03305	0.225095886
Tfrc	0.08546622	0.032717923	0.03102341	0.225095886
Tpd52	0.05601058	0.02176312	0.03294019	0.225095886
Rabgap1	0.113738	0.044451463	0.03371505	0.227680151
Snap25	-0.0379688	0.014884707	0.03412897	0.229328747
Erc2	-0.05728	0.022681794	0.03551036	0.233184625
Gabbr2	-0.064395	0.025428701	0.03512531	0.233184625
Ivd	0.0886268	0.035097673	0.03552285	0.233184625
Rps23	0.1098316	0.043508572	0.03556606	0.233184625
Syn1	-0.0319532	0.012611761	0.03505818	0.233184625
Cacna1a	-0.0633949	0.025174388	0.03590768	0.234287063
Cdh11	-0.0801146	0.032084975	0.03711799	0.235361909
Ctnnb1	-0.043354	0.017348181	0.03699667	0.235361909
Dlgap4	-0.0642573	0.02573115	0.03710022	0.235361909
Dpp10	0.0287268	0.01149539	0.03700055	0.235361909
Hccs	-0.1071832	0.042916712	0.03708796	0.235361909
Trio	-0.0834854	0.033400882	0.03697108	0.235361909
Ndel1	-0.0473374	0.019001595	0.0374502	0.236358784
Atp2b2	-0.0586214	0.023566841	0.0376715	0.236649604
Aifm1	-0.0466084	0.01878758	0.03806362	0.236870288
Atp5d	-0.0718216	0.028956335	0.03809137	0.236870288
Kalrn	-0.0354628	0.014328237	0.03840815	0.236870288
Ppp1cc	-0.075145	0.030350716	0.03835673	0.236870288
Ipo7	-0.0921053	0.037327177	0.03886083	0.238572718
Grn	0.0972266	0.039571822	0.03950526	0.240343999
Rab14	-0.0652238	0.026530079	0.03941176	0.240343999
Acaa1a;Acaa1b	0.06191626	0.025269291	0.03992072	0.241782506
Pebp1	0.0782666	0.032040491	0.04039149	0.24354165
Gria1	0.056572	0.023211918	0.0407423	0.244565048
Lap3	0.051597	0.021256632	0.0413738	0.246092267
Ndufs4	-0.0934358	0.038471346	0.04128511	0.246092267
Pck2	0.0428754	0.017689392	0.04160325	0.246092267
Stxbp5l	-0.0409416	0.016904682	0.04172555	0.246092267
Aldh1l1	0.0545274	0.022686274	0.04293877	0.247032381
Ctsb	0.1210888	0.050234562	0.04247718	0.247032381
Got2	-0.0444588	0.018491181	0.04288634	0.247032381
Hist1h2ah;H2afv;H2afz;H2afj;Hist2h2ac;Hist1h2ak;Hist1h2af;Hist3h2a;Hist2h2aa1;Hist2h2ab;H2afx	-0.2216626	0.09242259	0.04328742	0.247032381
Plxnb1	-0.049984	0.020818566	0.04311391	0.247032381
Trappc9	-0.0860888	0.035791353	0.04282133	0.247032381
Uqcrb	-0.0628218	0.026203477	0.04334818	0.247032381
Ywhaq	0.0445394	0.018516317	0.04281351	0.247032381
Adprh	0.0892874	0.037389807	0.04399195	0.248242228
Lphn1	-0.0570514	0.023908191	0.04411188	0.248242228
Rhob	0.0243068	0.010177924	0.04398001	0.248242228
Mapre1	0.1236808	0.052006514	0.04467246	0.250353822
Acadsb	0.093487	0.039746993	0.04653196	0.250882695
Daam1	-0.0652925	0.027807574	0.0468264	0.250882695
Gda	0.0638516	0.027204473	0.04689311	0.250882695
Lgi1	-0.0538394	0.022811673	0.04594611	0.250882695
Ndrg4	0.07236172	0.030665476	0.04597882	0.250882695
Ndufc2	-0.0812619	0.034523377	0.04640363	0.250882695
Ndufs8	-0.041149	0.017365268	0.04527362	0.250882695
Opa3	-0.0724738	0.030722129	0.04602932	0.250882695
Slc25a18	-0.0580452	0.024626908	0.04617518	0.250882695
Snca	0.0962716	0.040827617	0.04610179	0.250882695
Syt12	-0.0673394	0.028707631	0.04699589	0.250882695
Ywhah	0.0719456	0.030580254	0.04648626	0.250882695
Ampd2	0.08637446	0.036990315	0.04778305	0.251055522
Atp5j	-0.0452426	0.019431461	0.0482884	0.251055522

Ide	0.2927212	0.125526002	0.0480148	0.251055522
Map4	-0.073338	0.031461404	0.04808306	0.251055522
Ncl	0.1489958	0.06370954	0.04751399	0.251055522
Tubb2a	0.1067736	0.045712332	0.04772965	0.251055522
Uqcr10	-0.051993	0.022335944	0.04832944	0.251055522
Asap1	-0.0583791	0.025122988	0.04863529	0.251676313
Cpe	0.09833	0.042431613	0.04912023	0.253215586
Rph3a	-0.0737644	0.031865592	0.04931349	0.253245298
Ndufa8	-0.0538148	0.023314881	0.04983093	0.25493324
Cox7c	-0.064618	0.028028517	0.05004423	0.255058319
Ptprd	-0.0339344	0.014783014	0.05082666	0.257183788
Slc6a17	-0.0536922	0.023392187	0.0508421	0.257183788
Slc12a5	-0.0529926	0.023212073	0.05183002	0.26120288
Mapt	-0.0454196	0.019997181	0.05278322	0.263061901
Pfn2	0.0406912	0.01791421	0.05277102	0.263061901
Slc25a12	-0.0475244	0.020885767	0.05244291	0.263061901
Slc14a1	0.13236538	0.058403966	0.05319039	0.264029038
Slirp	-0.0879439	0.038840397	0.05336825	0.264029038
Aldh7a1	0.0639336	0.028339444	0.05405932	0.265255502
Atp5b	-0.0383382	0.017069555	0.05490907	0.265255502
Bcap31	-0.025128	0.011150513	0.0542675	0.265255502
Eef1a2	-0.07626	0.0339365	0.05481155	0.265255502
Rhot1	-0.0467967	0.020824454	0.0548063	0.265255502
Sars	-0.042363	0.018875979	0.05505646	0.265255502
Skp1	0.0738232	0.032758962	0.05426781	0.265255502
Tubb3	0.0463788	0.020679348	0.05518733	0.265255502
Vti1b	-0.0451224	0.020147883	0.05546348	0.265637477
Tmem163	-0.0429455	0.019273775	0.0564567	0.269438951
Slc27a1	0.0717686	0.032310038	0.05707091	0.271411247
Ntm	-0.0588594	0.026588826	0.05774776	0.272709627
Sh3gl3	0.11712858	0.052898092	0.05769902	0.272709627
Anp32a;Anp32c	0.0546752	0.024844798	0.05893231	0.272787252
Mtch2	-0.045945	0.020803417	0.0582142	0.272787252
Nlgn4l	-0.0776118	0.035232451	0.05873231	0.272787252
Prrt2	0.0550984	0.025009007	0.05870553	0.272787252
Sptan1	-0.0414544	0.018841245	0.05897603	0.272787252
Tjp2	-0.1374196	0.062243124	0.05828177	0.272787252
Clptm1	0.043939	0.02010659	0.06036135	0.277099051
Cmpk2	0.05917364	0.027126335	0.0607289	0.277099051
Mtnd2	-0.1254802	0.057501078	0.0606519	0.277099051
Ndufa2	-0.0740622	0.033888426	0.06034551	0.277099051
Aacs	-0.1039954	0.048066447	0.06244052	0.278858072
Adcy1	0.130292	0.060892634	0.06480662	0.278858072
Atp5f1	-0.0818246	0.038305708	0.0651716	0.278858072
Cttnbp2	-0.0544309	0.025457331	0.06496604	0.278858072
Dlg1	-0.047665	0.022259965	0.06464658	0.278858072
Exoc6	0.2714134	0.126741187	0.06462752	0.278858072
Fam162a	-0.0686607	0.032139949	0.06515032	0.278858072
Fbxo2	0.06648676	0.031125635	0.06517332	0.278858072
Gdi1	0.0727168	0.033517844	0.06186718	0.278858072
Gja1	0.0510988	0.023789687	0.06398034	0.278858072
Hspa1a;Hspa1b	-0.0577099	0.026730334	0.06289067	0.278858072
Macrod1	0.05884474	0.027380577	0.06386061	0.278858072
Ppia	0.0364352	0.016847635	0.06253261	0.278858072
Prdx2	0.0499446	0.023099384	0.06257827	0.278858072
Slc25a51	-0.12194	0.05619863	0.06183743	0.278858072
Sncb	0.109434	0.050724741	0.06304341	0.278858072
Uqcrh	-0.0314942	0.014705715	0.06461218	0.278858072
Usmg5	-0.0390328	0.018257549	0.06498867	0.278858072
Usp7	0.09696	0.045406361	0.06524375	0.278858072
Vdac1	-0.0377292	0.017643182	0.06493216	0.278858072
Cox6b1	-0.0459178	0.021601156	0.06623383	0.282196719
Nfasc	-0.0377578	0.01780868	0.06680535	0.283490887
Timm9	-0.0241984	0.011421179	0.06695738	0.283490887
Cnnm1	-0.1326812	0.06272938	0.06733295	0.283980026
Pdxk	0.06387948	0.03028907	0.06797959	0.283980026

Rab1A	-0.0395262	0.018730447	0.06784536	0.283980026
Uba52;Rps27a;Ubb;Ubc	-0.0473576	0.022420346	0.06763504	0.283980026
Ufm1	-0.0805518	0.038219162	0.06812421	0.283980026
Actn4	-0.0551839	0.026230014	0.06852635	0.284078041
Stip1	0.0489576	0.02327489	0.06856838	0.284078041
Nme2	0.05266	0.025188172	0.06994584	0.28889863
Cbr1	0.0428588	0.020570846	0.07073228	0.29046944
Entpd2	-0.0673827	0.032427855	0.07134476	0.29046944
Fh	-0.0378778	0.018206754	0.07106813	0.29046944
Hist1h2bc;Hist2h2bb;Hist1h2bh;Hist1h2bm	-0.1746087	0.084272308	0.07200957	0.29046944
Iars2	-0.043543	0.021026275	0.07212981	0.29046944
Ndufs5	-0.0534616	0.025870872	0.07262538	0.29046944
Pura	-0.0587002	0.028349043	0.07215911	0.29046944
Rims1	-0.0327172	0.015803848	0.07220611	0.29046944
Sidt1	0.067883	0.032858947	0.07269171	0.29046944
Skt	-0.042493	0.020430359	0.0711268	0.29046944
Slc25a4	-0.0645766	0.031258495	0.07269186	0.29046944
Vamp3	-0.0535044	0.025934978	0.07301647	0.290905876
Caskin1	-0.0513586	0.025053678	0.07451482	0.292771598
Ckmt1	-0.0280594	0.013664207	0.07410475	0.292771598
Endod1	0.04851108	0.023652399	0.07439252	0.292771598
Hspa4	0.0454826	0.022192287	0.07456861	0.292771598
Ndufa5	-0.1192526	0.058144665	0.07439676	0.292771598
Agk	-0.0333504	0.016628055	0.07981192	0.29639945
Aldh1b1	0.0738125	0.036892618	0.08042411	0.29639945
Ccsap	0.0781488	0.038942981	0.07967861	0.29639945
Cotl1	0.0868258	0.042871043	0.07742221	0.29639945
Eci1	-0.1174856	0.058876496	0.08108475	0.29639945
Epb41l2	-0.052955	0.026173758	0.07767104	0.29639945
Exoc3	-0.0448275	0.022406852	0.08043925	0.29639945
F11r	0.1078294	0.053737075	0.0796957	0.29639945
Git1	-0.0435536	0.021735148	0.08003954	0.29639945
Gstm1	0.0683026	0.034041994	0.07971905	0.29639945
Ldhb	0.0598238	0.029496319	0.07707472	0.29639945
Nlgn3	-0.0507009	0.025209861	0.07913667	0.29639945
Pacsin1	0.0572246	0.028121426	0.07627384	0.29639945
Psap	0.0576596	0.028849227	0.0806844	0.29639945
Psat1	-0.0390298	0.019261019	0.07729219	0.29639945
Psma4	0.1436194	0.071533681	0.07955918	0.29639945
Psmc3	0.0374426	0.018772426	0.08119845	0.29639945
Rapgef4	-0.0395465	0.019489938	0.07696789	0.29639945
Rpl34	-0.0309136	0.015228383	0.07685738	0.29639945
Sept6	-0.037512	0.018654767	0.07917341	0.29639945
Sh3gl1	0.0575972	0.028510978	0.07803727	0.29639945
Stx1a	-0.0455188	0.022434661	0.07698256	0.29639945
Tpd52l2	-0.0606218	0.029757748	0.0760061	0.29639945
Ttc7b	-0.0364769	0.018287949	0.08119388	0.29639945
Tuba1b	0.0682862	0.03409538	0.08016781	0.29639945
Vcl	0.050436	0.025225628	0.08059171	0.29639945
Abcb8	-0.0658537	0.033240606	0.08290446	0.29661135
Afg3l2	-0.0311112	0.015828168	0.08492495	0.29661135
Arfgap3	-0.0758002	0.038419569	0.08395858	0.29661135
Atp6ap2	0.03601624	0.018315409	0.08480871	0.29661135
Ca2	-0.0971606	0.048924681	0.08229093	0.29661135
Cdk5	0.04680488	0.023780012	0.08457297	0.29661135
Crip2	-0.0585011	0.029800873	0.0852528	0.29661135
Ddt	0.05964988	0.030161198	0.0833445	0.29661135
Dgkq	0.0581526	0.029483988	0.08403824	0.29661135
Dlgap2	-0.0336685	0.017048727	0.08371485	0.29661135
Epdr1	0.06900505	0.035067494	0.084634	0.29661135
Glg1	-0.0377049	0.018987818	0.08231418	0.29661135
Mief1	0.1210982	0.060817171	0.08162317	0.29661135
Nap1l1	-0.106661	0.054140591	0.08433393	0.29661135
Osbp	-0.0688094	0.034957818	0.08455851	0.29661135
Pdhb	-0.0502212	0.025585801	0.08528088	0.29661135

Rps26	-0.1172152	0.059750906	0.08542914	0.29661135
Stmn3	0.03716028	0.018900641	0.08485581	0.29661135
Tpt1	0.05426754	0.027360697	0.08261127	0.29661135
Ado	-0.055973	0.028784954	0.08773132	0.299422569
Got1	0.0384948	0.019800983	0.08779067	0.299422569
Mapk10;Mapk9	0.1102049	0.056506306	0.08695032	0.299422569
Nme1	0.10168518	0.052175078	0.08713713	0.299422569
Ptprs	-0.0411876	0.021168065	0.08756626	0.299422569
Sccpdh	-0.0361058	0.018517394	0.08701482	0.299422569
Tufm	-0.035536	0.018220851	0.0869528	0.299422569
Ckap4	0.07749368	0.039935698	0.08828338	0.299804729
Isca1	-0.0457928	0.02360458	0.08834667	0.299804729
Arsb	-0.0753298	0.038959108	0.08922895	0.300807975
Kiaa0513	0.061854	0.031999476	0.08931047	0.300807975
Tm9sf3	-0.1074046	0.055479981	0.08890509	0.300807975
Sv2a	-0.0272724	0.014128535	0.08967826	0.301295385
Cox6c	-0.0610246	0.031683823	0.0902693	0.301779723
Dync1i1	0.0411254	0.021345407	0.09018388	0.301779723
Carkd	-0.0392668	0.020533324	0.09219717	0.301899156
Ckb	0.0364724	0.019012956	0.09135586	0.301899156
Cops6	0.1162656	0.060928319	0.09278312	0.301899156
Eef1a1	-0.0418014	0.021890511	0.09259294	0.301899156
Emb	-0.161114	0.084275671	0.09228197	0.301899156
Maoa	-0.0534838	0.028027692	0.09278131	0.301899156
Ndufb3	-0.0591736	0.03088693	0.09170565	0.301899156
Npepps	0.0536728	0.028057932	0.09211454	0.301899156
Ntrk3	-0.0659545	0.034452683	0.09191279	0.301899156
Pllp	-0.1570437	0.082359409	0.09298735	0.301899156
Specc1	-0.0955924	0.049774037	0.09104147	0.301899156
Tubb4b	0.0609672	0.031727898	0.09089596	0.301899156
Degs1	-0.1454398	0.076489561	0.09376067	0.30367986
Gpd1l	0.052795	0.027859992	0.09469258	0.305234253
Tkt	-0.0543674	0.028688703	0.09468249	0.305234253
Scp2	0.03820527	0.020200566	0.09523562	0.306253785
Bcas1	-0.1084794	0.057582952	0.09632878	0.306778045
Dlst	-0.0487022	0.025819968	0.09598231	0.306778045
H3f3a;Hist1h3b;Hist1h3a;H3f3c	-0.2064312	0.109577476	0.09632833	0.306778045
Tmx2	-0.0383885	0.020392364	0.09653434	0.306778045
Ywhaz	0.0666352	0.035330946	0.09601082	0.306778045
Prickle2	-0.0363802	0.019359046	0.09701958	0.307596344
Cep112	-0.0636922	0.034043019	0.09826601	0.310092241
Timm44	-0.0338244	0.018068134	0.09809782	0.310092241
Agpat4	-0.1015086	0.054440284	0.09922909	0.312049622
Aldh2	0.0516398	0.027729958	0.09958759	0.312049622
Alg2	0.03988192	0.021519537	0.10096567	0.312049622
Atp5e	-0.0712038	0.038406484	0.10086286	0.312049622
Bin1	0.046012	0.024756702	0.10015071	0.312049622
Calcoco1	-0.2082414	0.111834816	0.0996173	0.312049622
Pde1a	0.0772878	0.041687566	0.10085904	0.312049622
Sst	0.12024716	0.064833799	0.10074741	0.312049622
Stam	-0.0723172	0.038899248	0.10007038	0.312049622
Arpc1a	-0.0425566	0.022989588	0.10130159	0.312373017
Dagla	-0.0873389	0.047301723	0.10203536	0.31391894
Arpc5l	-0.0524304	0.028656932	0.10470305	0.3142521
Atp6v1d	-0.0361152	0.019599015	0.1026188	0.3142521
Auh	-0.0488138	0.026533447	0.1030915	0.3142521
Clasp1	-0.0538567	0.029370096	0.10404153	0.3142521
Cyfip1	-0.0300264	0.016320902	0.10308469	0.3142521
Eno1	0.0599636	0.032759383	0.10456895	0.3142521
Faf2	-0.0697486	0.038008502	0.10382579	0.3142521
Ndufa6	-0.0672398	0.036737378	0.10459172	0.3142521
Pgd	0.05016028	0.027243797	0.10286171	0.3142521
Rab39b	-0.0324432	0.017726033	0.10459587	0.3142521
Sult4a1	0.0601716	0.032764175	0.10359892	0.3142521

Actg2;Acta1;Actc1;Acta2	0.1411744	0.077431798	0.10573118	0.314541951
Armc1	-0.0755432	0.041377803	0.10532952	0.314541951
Nrxn2	-0.0425404	0.023322264	0.10559918	0.314541951
Sptbn1	-0.0320626	0.01758282	0.10568116	0.314541951
Sbf1	-0.019056	0.010462754	0.10603793	0.314761201
Crtac1	0.07137483	0.039245684	0.10646913	0.315348089
Cacng8	-0.0384296	0.021279222	0.10855797	0.315385812
Kif5b	0.06016008	0.033153726	0.10713727	0.315385812
Lphn3	-0.0377566	0.020839878	0.10760256	0.315385812
Macf1	-0.03087	0.017070817	0.10816339	0.315385812
Ncan	0.047215	0.026137282	0.10848242	0.315385812
Ndufs1	-0.0354686	0.019643139	0.10861109	0.315385812
Pdhx	-0.0365846	0.02016983	0.10726061	0.315385812
Plp1	-0.1901138	0.105442594	0.1090505	0.315385812
Rps9	0.02808652	0.015560754	0.10872567	0.315385812
Tppp3	0.0716836	0.039738524	0.10890486	0.315385812
Vars2	-0.1101852	0.060771023	0.10737674	0.315385812
Rilp	-0.0600613	0.033391959	0.10977519	0.3168033
Rbp3	-0.0705942	0.039320069	0.1103303	0.317726401
Usp5	0.0381352	0.021275304	0.11082184	0.318462899
Hras	-0.0282858	0.015801806	0.11123342	0.318966993
Dpysl2	0.0411832	0.023042612	0.11170543	0.319641855
Scarb2	0.0741756	0.041578238	0.11226305	0.319882023
Stx12	-0.061676	0.034568577	0.1122351	0.319882023
Cnksr2	0.0246422	0.013837307	0.11280369	0.320745848
Nckap1	-0.0315058	0.017730501	0.11348125	0.321994532
Cmpk1	0.08748628	0.049370734	0.11433227	0.322226683
Rbm33	-0.0461569	0.026013349	0.11392756	0.322226683
Rnh1	0.04345244	0.024520608	0.11432333	0.322226683
Thop1	0.1062948	0.060020802	0.11451737	0.322226683
Acadvl	0.07249986	0.041021279	0.11514642	0.323323087
Fkbp1a	0.0544616	0.030885703	0.1158582	0.323974625
Pmpcb	0.03251928	0.018430691	0.11566739	0.323974625
Bdh1	-0.0359374	0.020432817	0.11665837	0.324115544
Hsd17b11	0.0468792	0.026621374	0.11627651	0.324115544
Pgk1	0.0526936	0.029979985	0.1168685	0.324115544
Suclg1	-0.0358306	0.020379434	0.11677095	0.324115544
Ahcyl2	-0.051869	0.029616605	0.11799032	0.325669678
Basp1	0.07959	0.04549324	0.11832443	0.325669678
Mdh2	-0.0319498	0.018248829	0.11809142	0.325669678
Vps52	0.1147746	0.065618944	0.11839339	0.325669678
Slitrk1	-0.0745772	0.04270531	0.11889619	0.326388017
Mtx1	-0.0392912	0.022551571	0.11962867	0.327069218
Ndufs6	-0.0782216	0.044872215	0.11946043	0.327069218
Gpc1	-0.0374524	0.02152394	0.12003807	0.327525525
Agap3	-0.0445371	0.025622926	0.12037814	0.327791208
Ykt6	-0.0625218	0.036000747	0.12065143	0.327874347
Lnp	-0.0565416	0.032623497	0.1212992	0.328972766
Arpc4	-0.040029	0.023151784	0.12207055	0.329786655
Ndufa13	-0.0585059	0.033840149	0.12208765	0.329786655
Cck	-0.1281374	0.074445324	0.12351525	0.331467157
Dst	-0.0532213	0.030882486	0.12311826	0.331467157
Nucb1	0.07981186	0.046394461	0.12369145	0.331467157
Ppp2cb	0.04262282	0.024744202	0.12327014	0.331467157
Dbi	0.0648648	0.037887708	0.12525041	0.331556505
Gstm5	0.0671858	0.039149489	0.12447305	0.331556505
Kcna1	0.0646218	0.037727655	0.12509446	0.331556505
Kif21a	-0.0426941	0.024941721	0.12530257	0.331556505
Ppp5c	0.04666022	0.027254984	0.12525782	0.331556505
Rab4a	-0.074376	0.043468994	0.12544319	0.331556505
Shank1	-0.0325388	0.019009257	0.12530606	0.331556505
Htt	0.2143448	0.125380187	0.12571984	0.331638706
Uggt1	0.05469358	0.032049858	0.12630075	0.332521658
Acot11	0.0624856	0.036760793	0.12759357	0.334524342
Nt5m	-0.064465	0.037893008	0.1273142	0.334524342

Purb	-0.0614394	0.036183987	0.12794493	0.334524342
Rab2a;Rab2b	-0.0436548	0.025718349	0.12805216	0.334524342
Efr3b	-0.0197906	0.011739948	0.13033189	0.336364181
Ktn1	0.07858952	0.046644924	0.13050973	0.336364181
Mal2	-0.0384362	0.022818569	0.13059253	0.336364181
Ociad2	0.05837874	0.034543595	0.12949668	0.336364181
Sdhb	-0.0322406	0.019117083	0.13018767	0.336364181
Slc2a1	-0.0656822	0.03901214	0.13074879	0.336364181
Tenm4	-0.0428856	0.02538085	0.12955889	0.336364181
Tmem55b	-0.0563536	0.033348234	0.1295259	0.336364181
Arhgdia	0.0542702	0.032283032	0.13125535	0.336776749
Rpl3	-0.0501995	0.029875186	0.13140786	0.336776749
Tfam	-0.0368974	0.021977805	0.13169749	0.336879772
Chchd3	-0.0474228	0.028365484	0.13309624	0.338600749
Vamp7	-0.051425	0.030755926	0.13305901	0.338600749
Vps4a	0.056494	0.033793965	0.13312238	0.338600749
Uqcrq	-0.0522772	0.031421432	0.13473313	0.342053568
Dnm1l	0.0374942	0.02260572	0.13577826	0.343328531
Hnrnph2;Hnrnph1;Hnrnpf	-0.0670778	0.040468225	0.13599794	0.343328531
Nebl	-0.0371742	0.022424	0.13594799	0.343328531
Gdi2	0.048424	0.029242361	0.13632372	0.34343992
Ndufs2	-0.0352086	0.021291912	0.13680491	0.34343992
Rab11fip5	-0.061373	0.037097243	0.13664683	0.34343992
Wipf3	0.1153404	0.069836247	0.13722366	0.343852021
Ctnna1	0.0948152	0.057709782	0.13901431	0.344499443
Cyc1	-0.0550844	0.033601917	0.13977765	0.344499443
Osbpl2	-0.128155	0.077725345	0.13779584	0.344499443
Phyhip	0.03048042	0.018560893	0.13917761	0.344499443
Ppp1r1b	0.17005128	0.103712136	0.13970965	0.344499443
Sucla2	-0.0373756	0.022764895	0.1392564	0.344499443
Tmem33	-0.1053842	0.064195659	0.1392981	0.344499443
Ttyh1	-0.0318493	0.019412665	0.13950012	0.344499443
Ttyh3	0.04641694	0.028233993	0.13879641	0.344499443
Arl8b	-0.0618752	0.037813827	0.14041165	0.345431674
Emd	0.05647836	0.034657746	0.14183346	0.346405621
Mtch1	-0.0368198	0.022544717	0.14107164	0.346405621
Pip5k1c	0.0350684	0.021505826	0.14161139	0.346405621
Slc6a11	-0.089499	0.054902343	0.14171721	0.346405621
Ap2a1	-0.0329798	0.020290504	0.14273496	0.346594014
Arf4	-0.0497939	0.030695735	0.14342287	0.346594014
Epn1	0.05141178	0.031649913	0.14294748	0.346594014
Prnp	0.0311738	0.019157773	0.14234287	0.346594014
Tst	-0.0516764	0.031858731	0.14345032	0.346594014
Txndc5	0.0810274	0.04995005	0.14342444	0.346594014
Brk1	-0.0338614	0.020893791	0.14375269	0.346625521
Ndufb11	-0.0537064	0.033184506	0.14423328	0.346625521
Sdha	-0.0382236	0.023616815	0.14421752	0.346625521
Nav1	-0.0735312	0.045469806	0.14450909	0.346671486
Snx12	-0.043748	0.027086823	0.1449514	0.347116037
Atp1a3	-0.0238084	0.014767005	0.14556816	0.347845745
Lrrc57	0.0410394	0.025469123	0.14577121	0.347845745
Fth1	0.08997037	0.055883868	0.14607431	0.347954242
Cdc42bpb	-0.0368607	0.022947457	0.14687329	0.34916075
Eef1b	0.0528388	0.032915467	0.14709785	0.34916075
Cap1	0.0576264	0.035928687	0.14740134	0.349267303
40787	0.0174446	0.01092537	0.1489982	0.349649567
Aak1	-0.0166432	0.010399689	0.14818727	0.349649567
Atad3	-0.030165	0.018893891	0.14903336	0.349649567
Iqsec1	-0.030625	0.019168413	0.14878122	0.349649567
Marc2	0.0475632	0.029798197	0.14911596	0.349649567
Tagln3	-0.0359082	0.022472777	0.14874279	0.349649567
Dync1i2	0.0499454	0.031323489	0.1494895	0.349917955
Cadm2	-0.0324252	0.020369207	0.150078	0.350262859
Calr	0.0486702	0.030617757	0.15058691	0.350262859
Capza2	0.0286628	0.018048857	0.15093353	0.350262859

Pfdn2	0.1215422	0.076519021	0.15086037	0.350262859
Rtn1	0.0429508	0.027018319	0.15056784	0.350262859
Rab12	-0.1895252	0.119859414	0.15248174	0.35324875
Homer1	-0.0338234	0.021529398	0.15481697	0.354403423
Mpp6	-0.0272618	0.017330226	0.15434603	0.354403423
Mrps23	0.1053458	0.066848754	0.15370223	0.354403423
Ppp2r5e	-0.0205518	0.013052327	0.15400332	0.354403423
Sec14l2	0.1008984	0.064130145	0.15428625	0.354403423
Sod1	0.073733	0.04676093	0.15349043	0.354403423
Stxbp1	-0.025268	0.01607249	0.15456485	0.354403423
Gap43	0.0873184	0.055666311	0.15537811	0.354664066
Rin1	0.095251	0.060778397	0.15570672	0.354664066
Tcp1	-0.0311508	0.019877562	0.15571861	0.354664066
Dstn	0.0609115	0.038920309	0.15620676	0.354763801
Llgl1	-0.059301	0.03795439	0.15681308	0.354763801
Rimbp2	0.04163974	0.026643004	0.15670833	0.354763801
Vps16	-0.0805122	0.051518133	0.15672783	0.354763801
Cops4	0.0387706	0.024888508	0.15790296	0.356036738
Ncam1	-0.0243614	0.015627587	0.15764482	0.356036738
Adss	0.0892562	0.057588658	0.15976264	0.356938157
Brinp1	-0.0441839	0.028441635	0.15891045	0.356938157
Cplx2	0.0570938	0.036876346	0.16015269	0.356938157
Dgke	0.076559	0.049428218	0.15999994	0.356938157
Emc2	-0.0603096	0.038926781	0.1599011	0.356938157
Ldha	0.0630234	0.040629099	0.15945523	0.356938157
Rasal1	0.0584186	0.03767758	0.15962151	0.356938157
Sec61b	-0.0701224	0.045372967	0.16081582	0.357825621
Unc13a	-0.040341	0.02613118	0.1612171	0.358128501
Gnai1	-0.0307874	0.020004398	0.16235837	0.359797
Myl12b	0.04894318	0.03183704	0.16277499	0.359797
Pcbp1	-0.051706	0.03365769	0.16303378	0.359797
Snx6	0.084793	0.055132699	0.16261167	0.359797
Abi2	-0.0278758	0.018199938	0.16414608	0.360861675
Arhgap32	-0.0370564	0.024309218	0.16592087	0.360861675
Clip2	-0.0272072	0.017768904	0.16426118	0.360861675
Dnm3	-0.0356876	0.023336258	0.16472215	0.360861675
Fam92b	0.0315962	0.020672946	0.16494007	0.360861675
Glul	-0.0258074	0.016899637	0.16525439	0.360861675
Rab3d	-0.0570197	0.037358815	0.16545628	0.360861675
Rgs14	0.0419441	0.027464022	0.1652201	0.360861675
Scn3a;Scn9a	0.020239	0.013268258	0.16567758	0.360861675
Rp2	-0.0912731	0.059919081	0.16619245	0.360871225
Grb2	0.0777423	0.051078995	0.16650528	0.360970168
Psd	-0.0564024	0.037158394	0.16752013	0.36258829
Dhrs7	0.0861266	0.0568217	0.16805511	0.363164218
Fbxl16	0.0221806	0.014666064	0.16889098	0.364387516
Ranbp1	0.04627054	0.030671548	0.16983966	0.364719144
Wfs1	0.1005568	0.066659174	0.16985481	0.364719144
Ywhab	0.0506018	0.033521697	0.1696037	0.364719144
Rpl27	-0.0644136	0.042784864	0.17060804	0.365755021
Arpc3	-0.0788177	0.052514215	0.17177941	0.36652091
Ndufaf2	-0.055919	0.037246464	0.1716681	0.36652091
Uso1	-0.0720716	0.04800991	0.17170391	0.36652091
Atcay	-0.0738992	0.049322907	0.17244156	0.366658484
Coa7	-0.055944	0.037320886	0.17225719	0.366658484
Ppp3cb	0.0431284	0.028801788	0.17265831	0.366658484
Dhrs1	-0.0260116	0.017384994	0.17296665	0.366736633
Msrb2	-0.0593012	0.039742335	0.17400587	0.36836179
Stx16	-0.0743102	0.049839187	0.17429888	0.368404646
Hacd2	0.05815768	0.039095482	0.175178	0.369016751
Mtdh	0.06396988	0.043002775	0.17517927	0.369016751
Ogt	-0.0325216	0.021875201	0.17540814	0.369016751
Fyn	-0.0264912	0.017855424	0.17619356	0.370092619
Gpm6b	-0.028914	0.019555662	0.17751944	0.370153214
Hsp90b1	0.0368466	0.024925525	0.17759273	0.370153214
Lin7b	-0.0424156	0.02863465	0.17681186	0.370153214

Marcks	0.0453443	0.030632126	0.17706698	0.370153214
Robo2	-0.0589733	0.039885737	0.17751779	0.370153214
Ercc6l	-0.1116818	0.075754254	0.17863984	0.371761986
Idh3a	-0.0261704	0.017776203	0.17917787	0.37230801
Slc25a11	-0.04219	0.028678534	0.17946212	0.372325824
Apoe	0.0346128	0.023552044	0.17985873	0.372576346
Stt3a	0.08565216	0.058333912	0.18020788	0.372727933
Lin7a	-0.0369072	0.025179764	0.18088551	0.37355743
Myo18a	-0.0249936	0.01708923	0.18173933	0.374747711
Rpl32;Rpl32-ps	0.0526146	0.036010112	0.18212017	0.37496053
Ap2b1	-0.0362188	0.024823527	0.18266909	0.375518244
Gars	0.05437092	0.037297412	0.18301302	0.375653515
Gabrb3	-0.0546435	0.037536846	0.18355933	0.375766809
Wasf1	-0.0438574	0.030132456	0.18362466	0.375766809
Lancl1	-0.0134704	0.009269683	0.18424896	0.376473953
Asrgl1	-0.0157684	0.010878478	0.1852386	0.377354315
Ndufs7	-0.023027	0.015875994	0.18498834	0.377354315
Acyp1	0.04912609	0.033929484	0.18567678	0.377657183
Ap2s1	-0.0612281	0.042394277	0.18666528	0.377657183
Nt5c	0.086667	0.059977396	0.18646344	0.377657183
Phpt1	0.0421345	0.029134736	0.18613675	0.377657183
Tars2	-0.1042576	0.072209886	0.18678537	0.377657183
Ap1b1	-0.0221834	0.015392353	0.18750055	0.377971549
Camk2d	0.0913356	0.063339928	0.18728312	0.377971549
Rpl14	-0.0234521	0.016317759	0.18859387	0.379608929
Cox5a	-0.0514866	0.035888932	0.18931114	0.380485633
Glod4	-0.0424058	0.029642823	0.19043223	0.381603114
Ncdn	0.0698374	0.048812694	0.19038691	0.381603114
Fam81a	-0.0928436	0.064989026	0.19097525	0.382124319
Rtcb	0.0845248	0.059234581	0.19143586	0.382254702
Tppp	-0.025729	0.018038495	0.19160646	0.382254702
Slc9a3r1	-0.0284504	0.019990525	0.19248545	0.383441908
Kcnd3	0.1503308	0.105764669	0.19299685	0.383894413
Erp29	0.04822874	0.033971269	0.19346866	0.384266987
Arhgap23	0.056196	0.039771641	0.19536913	0.385209268
Atp6v1e1	-0.0206664	0.014622305	0.19526101	0.385209268
Gpd2	-0.0254386	0.017953133	0.19424298	0.385209268
Lysmd2	0.047559	0.033648131	0.19523999	0.385209268
Timm10	-0.0557144	0.039380771	0.19486061	0.385209268
Calu	0.04917452	0.034876912	0.19622831	0.386339323
Cox7a2	-0.0616232	0.043800055	0.19709152	0.386349257
Gpr37l1	-0.0713028	0.050640349	0.19677635	0.386349257
Igsf8	-0.0266584	0.018945099	0.19702889	0.386349257
Mpst	0.0314222	0.02238783	0.19806057	0.387686162
Mdh1	0.0401116	0.028638582	0.1989023	0.388770325
Ezr	0.0414632	0.029637848	0.19936951	0.389120404
Abat	0.0502758	0.035995832	0.20003001	0.389284436
Gstp2;Gstp1	0.052278	0.037428657	0.20002261	0.389284436
Phgdh	-0.0315038	0.022584805	0.20055218	0.389739063
F3	0.0842738	0.060577627	0.20164096	0.391291905
Atp6v1b2	-0.017067	0.012310597	0.20304662	0.393454343
Ncln	0.0459164	0.033167531	0.203631	0.394021413
Brsk1	0.02950546	0.021364243	0.20460711	0.395257577
Ccdc58	-0.0516373	0.037453038	0.20530133	0.395257577
Pgbd5	-0.0328632	0.023835279	0.20528897	0.395257577
Ran;Rasl2-9	0.05306382	0.038500813	0.20544045	0.395257577
Frrs1l	0.0737528	0.053638539	0.2064077	0.395428643
H2-Eb1	-0.1005796	0.073074067	0.20598835	0.395428643
Ndufb9	-0.0425054	0.03090196	0.20626019	0.395428643
Camk1d	-0.0283182	0.020628136	0.20706374	0.39570111
Fbxo41	-0.026305	0.019164998	0.20713588	0.39570111
Acsl3	0.0352704	0.025760629	0.20815191	0.395724836
Arfgap1	0.0497956	0.036359851	0.20804325	0.395724836
Slc25a22	-0.029412	0.021490607	0.20832028	0.395724836
Slc30a1	-0.072244	0.052705768	0.2076891	0.395724836
Chchd6	-0.0273464	0.020046966	0.20966891	0.396234534

Pitpnm1	0.0520742	0.038182944	0.20976209	0.396234534
Sec23a	-0.0182376	0.013369837	0.20967923	0.396234534
Tmem65	-0.0514718	0.037670066	0.20898542	0.396234534
Ank3	-0.0226088	0.016696487	0.21270911	0.399011076
Ctnnd1	-0.0318785	0.023533804	0.21256251	0.399011076
Gng5	-0.0454742	0.033562449	0.21246237	0.399011076
Mapk8ip3	0.0610856	0.045044139	0.21209191	0.399011076
Pmpca	-0.1171	0.086253533	0.21163576	0.399011076
Pa2g4	0.0369171	0.027331255	0.21374316	0.4003947
Bphl	0.0370911	0.027505757	0.21443243	0.401129529
Kars	0.0908421	0.067524896	0.21541044	0.402401709
Tecpr1	0.03921646	0.029206478	0.21620903	0.402779336
Timm13	-0.0343604	0.025573132	0.21593594	0.402779336
Abcb7	-0.0534784	0.040235909	0.22046199	0.404095951
Camkk2	-0.0604586	0.045457955	0.2201888	0.404095951
Crym	0.1342948	0.100668165	0.21891866	0.404095951
Dctn2	0.0250344	0.018832574	0.22040158	0.404095951
Dnaja3	-0.026421	0.019841436	0.21968004	0.404095951
Ndufb4	-0.0497386	0.037246054	0.21848945	0.404095951
Opalin	-0.1082543	0.080975886	0.21803163	0.404095951
Pgrmc1	0.0337542	0.025391868	0.22039611	0.404095951
Prkcsh	0.0217094	0.016323154	0.22019308	0.404095951
Usp14	0.0610778	0.04594341	0.22037009	0.404095951
Vps33a	-0.0845474	0.063618167	0.22050611	0.404095951
Zdhhc5	-0.0384182	0.028788011	0.21876614	0.404095951
Tom1l2	0.0486876	0.036677555	0.22098959	0.404433206
Dclk2	-0.0728403	0.055204139	0.22352108	0.407231767
Gria3	-0.0343648	0.026090453	0.22426525	0.407231767
Grin2b	-0.0495452	0.037608471	0.22418407	0.407231767
Oxr1	0.0282082	0.021405897	0.22406187	0.407231767
Rrbp1	0.0399384	0.030242946	0.22316603	0.407231767
Tuba1a;Tuba1c;Tuba3a;Tuba8	0.0471598	0.03581	0.22432787	0.407231767
Coro2b	0.1259235	0.095853311	0.22536441	0.408016634
Hk1	-0.0221046	0.016815052	0.22508786	0.408016634
Itpr1	-0.0239918	0.018292739	0.22605979	0.408310172
Psma5	0.08677326	0.066172149	0.22613118	0.408310172
Fam210a	-0.0784266	0.060072752	0.22800521	0.410583466
Map1s	0.06241756	0.047878124	0.22860617	0.410583466
Nbea	-0.0328389	0.025172377	0.22831842	0.410583466
Ndufb6	-0.0400918	0.030707192	0.22797636	0.410583466
Vcp	0.0276232	0.021242139	0.22967426	0.411953981
Acot9;Acot10	-0.0309992	0.024029398	0.23307058	0.413627513
Atp2b1	-0.0180958	0.014116093	0.23576869	0.413627513
Clpb	-0.0398068	0.030857652	0.23308397	0.413627513
Glud1	-0.0398284	0.030843419	0.23265578	0.413627513
Grm7	-0.108647	0.083851198	0.23120719	0.413627513
Kiaa1549	-0.0375798	0.029339867	0.23613016	0.413627513
Mag	-0.1135224	0.088538744	0.23568505	0.413627513
Manf	-0.054702	0.04260393	0.23508918	0.413627513
Mark1	-0.0306315	0.023852082	0.23500217	0.413627513
Ndrg1	-0.0716728	0.055985283	0.23634258	0.413627513
Ola1	0.04441211	0.034353297	0.23216284	0.413627513
Palm	-0.042555	0.033219175	0.23606569	0.413627513
Park7	0.033961	0.026221727	0.23139254	0.413627513
Pitrm1	0.03671374	0.028683529	0.23642607	0.413627513
Ppp2r2a	0.03251494	0.025243906	0.23373945	0.413627513
Reps2	0.0741208	0.057817143	0.23574881	0.413627513
Sfxn3	-0.0277764	0.021615874	0.23474523	0.413627513
Sirt2	-0.096092	0.074519798	0.23325908	0.413627513
Slc30a9	-0.0709491	0.054913751	0.2324247	0.413627513
Gpm6a	-0.0445696	0.034880183	0.23715189	0.414360606
Bai1	-0.0506262	0.040079272	0.24210362	0.415367583
Cdh13	0.0494336	0.038907614	0.23959453	0.415367583
Cnp	-0.1018558	0.080095956	0.23921118	0.415367583
Cyp46a1	0.03057704	0.024203485	0.24204206	0.415367583

Fscn1	-0.01981	0.015560629	0.23873447	0.415367583
Hspa8	-0.0234664	0.018524307	0.24086508	0.415367583
Itgav	-0.031061	0.024576079	0.2418585	0.415367583
Pcdhga4	0.040754	0.032105215	0.23998346	0.415367583
Prkar1b	0.0531352	0.042027016	0.2417085	0.415367583
Psmc5	0.0521536	0.041162919	0.24079212	0.415367583
Scrn1	0.03902166	0.030731675	0.23985983	0.415367583
Serinc5	-0.0477032	0.037684617	0.24118332	0.415367583
Stoml2	-0.0348702	0.027541364	0.24109798	0.415367583
Tmem30a	-0.0171383	0.013575403	0.24234131	0.415367583
Trim3	-0.0210989	0.016650094	0.24072812	0.415367583
Akr7a2	0.0405019	0.032153711	0.24330639	0.415926458
Cyth1	0.1195612	0.094860535	0.24304767	0.415926458
Gltp	-0.0604162	0.048063452	0.24420715	0.415926458
Gsta4	0.05562249	0.044240766	0.24411813	0.415926458
Vps26b	0.0728068	0.057906692	0.24410282	0.415926458
Rpl10a	0.02000686	0.015927871	0.24452272	0.415939405
Cisd1	0.05332252	0.042635288	0.24639506	0.418071227
Lasp1	-0.0196506	0.015704153	0.24617588	0.418071227
Abhd12	0.04947974	0.039666788	0.24753357	0.418938324
Acsl6	-0.0197776	0.015845802	0.24727504	0.418938324
Lgalsl	0.0484782	0.038891046	0.24783664	0.418938324
Eef2	-0.0286812	0.023033845	0.24830212	0.419200509
Sorbs1	0.03709008	0.029856533	0.24931432	0.420383896
Sco1	-0.0416636	0.033578864	0.24984221	0.420748719
Ndufa1	0.0675056	0.054595683	0.25135389	0.422767335
39326	-0.0333282	0.027269069	0.25641851	0.423070701
Aqp4	0.0669504	0.055030334	0.25842561	0.423070701
Cycs	-0.0399708	0.032386128	0.25215378	0.423070701
Dlg3	-0.023705	0.019465051	0.25798828	0.423070701
Ethe1	0.04183102	0.033956651	0.25296689	0.423070701
Fxyd7	0.0484342	0.039380205	0.25366938	0.423070701
Gad2	0.0446062	0.0362372	0.2533013	0.423070701
Mtpn	0.0585402	0.04778521	0.25539234	0.423070701
Napb	0.035811	0.029389624	0.2577482	0.423070701
Nptxr	0.0497518	0.040333072	0.25239088	0.423070701
Nrxn3	-0.0306488	0.025162189	0.25790665	0.423070701
Pim3	0.6876888	0.561241552	0.25531039	0.423070701
Psmc6	0.1399672	0.113942115	0.25420418	0.423070701
Rmdn3	-0.0315259	0.025893994	0.25810529	0.423070701
Rpl17	0.05347888	0.043839219	0.25724695	0.423070701
Rtn4	0.0181826	0.014889641	0.25679108	0.423070701
Sh3bgrl3	0.0441162	0.036037714	0.25571427	0.423070701
Slc7a14	0.08166144	0.066977488	0.25748018	0.423070701
Tmed2	0.074785	0.061454075	0.25831178	0.423070701
Tmem11	-0.0446585	0.03668096	0.25811011	0.423070701
Ubqln2	0.060887	0.049648712	0.25493364	0.423070701
Vcam1	-0.0382952	0.031198589	0.25453829	0.423070701
Camkv	-0.0381626	0.031496399	0.26021749	0.423344001
Cit	-0.0234366	0.019296169	0.25915936	0.423344001
Gabarapl2	-0.0538604	0.044439684	0.2600943	0.423344001
Hmgcl	0.0270518	0.022397242	0.26160881	0.423344001
Ly6h	0.0482606	0.040001849	0.26210386	0.423344001
Mcu	-0.050978	0.042122548	0.26073207	0.423344001
Nomo1	-0.0232836	0.019322108	0.26262844	0.423344001
Otud7a;Otud7b	0.0976458	0.08103959	0.26266734	0.423344001
Pfkm	-0.0395578	0.032788565	0.26210682	0.423344001
Slc44a1	-0.047459	0.03920284	0.26059768	0.423344001
Synpr	-0.037609	0.031084722	0.26085732	0.423344001
Tnpo2;Tnpo1	0.0450374	0.037177914	0.26030723	0.423344001
Wars	0.032231	0.026717039	0.2621314	0.423344001
Acot13	-0.0268594	0.022333228	0.26348981	0.423851969
Hibch	0.02780333	0.02312497	0.26362072	0.423851969
Rabl6	0.068646	0.057134522	0.26392398	0.423851969
Rps3a	0.0441436	0.036787066	0.26447588	0.424233871
Atp6v1f	0.083194	0.069512449	0.26563642	0.424840992

Hsp90ab1	-0.0513536	0.042971619	0.26628687	0.424840992
Rpl23	0.02929652	0.024473323	0.26554129	0.424840992
Serbp1	0.0337298	0.028215444	0.26614694	0.424840992
Slc25a1	-0.0286382	0.023971464	0.26642714	0.424840992
Acad8	0.0426258	0.036537174	0.27695226	0.428790006
Anks1b	-0.027843	0.023602663	0.2720256	0.428790006
Astn1	-0.0294886	0.024929278	0.27081195	0.428790006
Blmh	0.0470082	0.039820917	0.27171189	0.428790006
Chmp2b	0.03202596	0.027499301	0.27772809	0.428790006
Cntnap1	0.040955	0.034846365	0.27366562	0.428790006
Dctn3	0.0990118	0.083927366	0.27199667	0.428790006
Faah	-0.0495012	0.042146181	0.27396404	0.428790006
Gabra4	0.0556664	0.047759509	0.27736652	0.428790006
Glrx5	0.02199092	0.018720448	0.27389284	0.428790006
Kiaa1107	-0.0277365	0.023769036	0.27684757	0.428790006
Kiaa1468	0.0517412	0.044255297	0.27599608	0.428790006
Krt79	-0.4708872	0.40413407	0.2775119	0.428790006
Mapre3	0.0202216	0.017349976	0.27738358	0.428790006
Mbp	-0.1085176	0.091545296	0.2698743	0.428790006
Mob4	0.02616564	0.022470577	0.27779303	0.428790006
Mras	-0.0564251	0.048347842	0.27679107	0.428790006
Olfm1	-0.0406772	0.034305316	0.26974643	0.428790006
Pcmt1	0.03298179	0.02830364	0.27747045	0.428790006
Ptpn9	-0.0519088	0.044561527	0.27762515	0.428790006
Sfxn5	-0.035936	0.030559876	0.27343189	0.428790006
Smap2	-0.0350547	0.029661809	0.27121546	0.428790006
Sugt1	-0.095535	0.081458399	0.27460912	0.428790006
Triap1	-0.0538247	0.045560506	0.27137425	0.428790006
Ubxn4	-0.0546936	0.046493581	0.27326292	0.428790006
Uchl1	0.0605164	0.051077717	0.27010112	0.428790006
Ugt1a7c	-0.0297389	0.025472051	0.27662158	0.428790006
Wbp2	0.0667754	0.057237179	0.27695156	0.428790006
Arrb1	0.034009	0.029392242	0.28062079	0.429717079
Bsg	-0.036015	0.031125336	0.28061234	0.429717079
Dhrs4	0.0372554	0.032162929	0.28013562	0.429717079
Gstk1	0.05291472	0.045666009	0.27998183	0.429717079
Mrpl12	0.03793464	0.0326815	0.27921154	0.429717079
Taldo1	0.05297758	0.045733533	0.28011125	0.429717079
Vta1	0.0783412	0.067533677	0.2794826	0.429717079
Pex5l	-0.0379727	0.032969717	0.28268094	0.432381575
Ank2	-0.0256652	0.022351469	0.28403678	0.432471883
Atp2b4	-0.029247	0.025447711	0.2836313	0.432471883
Cul3	0.189232	0.164502508	0.28323062	0.432471883
Gng4	-0.034218	0.029820271	0.284341	0.432471883
Ptprn2	0.07678924	0.066900683	0.28421049	0.432471883
39692	-0.0279116	0.024379934	0.28536128	0.43311036
Cyfip2	0.017211	0.015077813	0.28668484	0.43311036
Ndufa7	-0.0469004	0.041047876	0.28625389	0.43311036
Ndufv2	-0.0307676	0.026924307	0.28618873	0.43311036
Psmc2	0.065047	0.05684752	0.28560485	0.43311036
Rpl4	0.0413347	0.036209132	0.28665502	0.43311036
37500	-0.0226274	0.01991992	0.28887232	0.433226939
Ass1	0.03301576	0.029056264	0.28873353	0.433226939
Cept1	-0.0490266	0.042991127	0.28711249	0.433226939
Dtna	-0.0319051	0.028111447	0.28925412	0.433226939
Fam49b	0.0491558	0.043400631	0.29018187	0.433226939
Hint2	0.033353	0.029272918	0.28750854	0.433226939
Mrps5	0.01979608	0.017482579	0.2902904	0.433226939
Pacsin2	0.0295341	0.026053486	0.28979028	0.433226939
Ppp1r9b	-0.0288674	0.025429924	0.28916593	0.433226939
Syngr1	-0.0376268	0.033209318	0.29001807	0.433226939
Syt2	-0.0844238	0.074132912	0.28773034	0.433226939
Kcnq2	0.0694622	0.061453325	0.29108646	0.433935483
Aldh6a1	0.0443582	0.039357892	0.29239006	0.434594943
Cyth2	0.0466722	0.041539479	0.29378126	0.434594943
Dync1li1	0.03184746	0.028268045	0.29255868	0.434594943

Prkcg	-0.0523396	0.04652281	0.29319436	0.434594943
Rab22a	0.0582056	0.051804005	0.29377691	0.434594943
Thy1	-0.018783	0.016688526	0.29300555	0.434594943
Vps29	0.03257648	0.028990757	0.29373224	0.434594943
Camk2g	-0.0397386	0.035430339	0.29456786	0.434806088
Rps15a	-0.031895	0.02842093	0.2943121	0.434806088
Napa	0.0203578	0.018184118	0.29539481	0.435550728
Golga7	-0.0680502	0.060909064	0.29631791	0.436392641
Nsfl1c	0.04320976	0.038700645	0.29661202	0.436392641
Ak3	0.0310256	0.027899756	0.29841987	0.438097999
Rap2b;Rap2c	0.033424	0.030053271	0.29837125	0.438097999
Psmb1	-0.0361717	0.032553744	0.29878527	0.438158166
Otub1	0.06191666	0.055884864	0.30008661	0.439589236
Dcun1d1	0.0878682	0.079429404	0.30077473	0.440119894
Cep170	0.02167431	0.019630574	0.30164509	0.440241291
Slc1a3	0.0539864	0.04890422	0.30172217	0.440241291
Timm50	-0.0233766	0.021181293	0.30183556	0.440241291
Bpnt1	-0.0308329	0.02797745	0.30248235	0.440283019
Rab3gap2	0.0802382	0.072865373	0.30284213	0.440283019
Tomm22	0.3535236	0.320994506	0.30277892	0.440283019
Rpl30	-0.0298402	0.027141259	0.3035574	0.440848367
Isca2	0.02842646	0.025898123	0.30430303	0.441456537
Coasy	0.039578	0.036200895	0.30609192	0.443445909
P33monox	-0.0785736	0.071926074	0.30645011	0.443445909
Pepd	-0.0992112	0.09092576	0.30698765	0.443445909
Ppp3ca	0.0238086	0.021818963	0.30696061	0.443445909
Tpm1	0.04996818	0.045944695	0.30846139	0.445098688
Add1	0.0172844	0.015908155	0.30890259	0.445160398
Krt75;Krt6a;Krt6b;Krt5	-0.5113059	0.471208484	0.30949295	0.445160398
Rpl24	0.03287614	0.030277874	0.30919324	0.445160398
Fsd1	-0.0518297	0.047990547	0.31162407	0.446886952
Letmd1	-0.04117	0.03812865	0.31172125	0.446886952
Ube2v2	-0.0240042	0.022244692	0.31200127	0.446886952
Vcpip1	-0.0572979	0.053099825	0.31201682	0.446886952
Acadl	0.0598974	0.055898166	0.31518311	0.447115889
C2cd2l	0.0474282	0.044079153	0.3133125	0.447115889
Cnpy2	0.0439382	0.041102194	0.31626143	0.447115889
Dlat	-0.0272644	0.025408268	0.31454608	0.447115889
Ech1	0.04061682	0.037822802	0.3142004	0.447115889
Fam163b	-0.0724444	0.067646541	0.31544524	0.447115889
Hsdl1	-0.0236131	0.021957538	0.31355622	0.447115889
Hsdl2	-0.0243401	0.022663296	0.31415055	0.447115889
Numbl	-0.038016	0.035536702	0.31593524	0.447115889
Ppm1h	0.03662636	0.034219675	0.31569714	0.447115889
Ppme1	0.0516328	0.048077278	0.31416536	0.447115889
Rpl5	0.06556104	0.06083385	0.31258548	0.447115889
Tubb5	0.0291112	0.027245355	0.31648027	0.447115889
Fam213b	0.0345264	0.032398697	0.31767462	0.447399261
Sec22b	0.0116624	0.010940397	0.3175377	0.447399261
Tmx4	0.0284362	0.026645608	0.31702495	0.447399261
Rpl19	0.03024586	0.028417114	0.31823625	0.447723377
Lin7c	-0.0242352	0.022789727	0.31863157	0.447813083
Farsb	0.10211854	0.096307358	0.31995037	0.448080603
Gdap1	-0.0222416	0.020985096	0.32014896	0.448080603
Mtfr1l	0.03523222	0.033207593	0.31968135	0.448080603
Rpl15	0.04225486	0.039825262	0.31966528	0.448080603
Capns1	0.02570032	0.024406705	0.32310261	0.448939222
Clpp	0.057581	0.054543724	0.3219473	0.448939222
Gapdh	0.0320176	0.030277074	0.32117333	0.448939222
Gcat	0.0526246	0.049834158	0.32181427	0.448939222
Hcn2	-0.036696	0.034795115	0.32240123	0.448939222
Pdk1	-0.0344571	0.032700754	0.32279748	0.448939222
Rpl7	-0.0242647	0.02305952	0.32342161	0.448939222
Sirpa	0.02962509	0.028122429	0.32291699	0.448939222
Bag6	0.0568484	0.054226533	0.32511372	0.449405754
Cfl2	0.01910648	0.018205792	0.32462765	0.449405754

Mog	-0.0931276	0.088818232	0.32504063	0.449405754
Pde2a	0.041935	0.04002714	0.32541115	0.449405754
Slc30a3	0.03017029	0.028798326	0.32542141	0.449405754
Bcat2	0.04269962	0.040850854	0.32645653	0.449915175
Mrps15	0.05832108	0.055795755	0.32645493	0.449915175
Dpysl4	0.0304496	0.029199403	0.32751889	0.450919177
Stxbp6	-0.0474348	0.045594728	0.32859183	0.451935682
Atp5j2	-0.0266948	0.02571187	0.32952287	0.452149073
Scfd1	0.0379038	0.03652617	0.3297473	0.452149073
Slk	0.01894636	0.018257905	0.3297513	0.452149073
Myo6	-0.0456899	0.044121175	0.33069529	0.452694826
Ube2d2b;Ube2d2;Ube2d3	-0.0271346	0.026210161	0.33081967	0.452694826
Ap1g1	0.01972106	0.019088049	0.331747	0.452704091
Mycbp2	0.0553962	0.053628101	0.33183199	0.452704091
Timm10b	0.0730552	0.070647614	0.33134394	0.452704091
Psd3	-0.0469936	0.045635692	0.33324944	0.45417909
Kdm4b	-0.062667	0.060908428	0.33363986	0.454252807
Vwa8	0.02934582	0.028562095	0.3342737	0.454657456
Cs	-0.0207552	0.020250023	0.33537865	0.454950229
Hspa9	-0.026745	0.026275441	0.33853112	0.454950229
Jup	-0.030215	0.029564867	0.33669348	0.454950229
Mark4	0.036871	0.036171728	0.33787823	0.454950229
Mical3	0.012757	0.012502929	0.33743697	0.454950229
Mobp	-0.0607467	0.059660161	0.33837845	0.454950229
Nt5dc3	0.0260606	0.025531741	0.33726177	0.454950229
Ogdh	-0.0202722	0.019867876	0.3374237	0.454950229
Pip4k2b	-0.032991	0.032201579	0.33557044	0.454950229
Sh3gl2	0.0502312	0.049003374	0.33533027	0.454950229
Slc12a9	0.0552814	0.054299355	0.33843494	0.454950229
Slc1a2	-0.0175696	0.017216932	0.33736432	0.454950229
Gnaz	-0.0124904	0.012285708	0.33907228	0.455195579
Mllt4	-0.0197572	0.019474405	0.34003111	0.455195579
Rab7a	-0.024895	0.024541584	0.34008524	0.455195579
Smap1	-0.0214758	0.021185516	0.34039882	0.455195579
Tomm6	-0.047036	0.04637687	0.3401697	0.455195579
Hnrnpk	-0.0262157	0.025886333	0.34083904	0.455333421
Dctn4	0.0627944	0.062118124	0.34166457	0.455535107
Rab4b	0.02887646	0.028552796	0.34146257	0.455535107
Rab3b	0.04341262	0.042993472	0.34217719	0.455768655
Slc2a3	-0.019591	0.019417137	0.34253569	0.455796666
Aip	-0.045525	0.045165414	0.34298371	0.455943622
Pea15	0.0610844	0.060820498	0.3446226	0.457671799
Lpcat1	-0.0368216	0.036705889	0.34516076	0.457936221
Abhd6	-0.0449572	0.044997207	0.34699842	0.458572442
Cndp2	0.02620732	0.02622448	0.34689147	0.458572442
Rogdi	0.03262968	0.032589311	0.34602998	0.458572442
Tmx1	0.03773538	0.03774744	0.34673897	0.458572442
Baiap2	0.0094778	0.009540085	0.3495752	0.459506029
Ksr1	0.03786672	0.038140614	0.34987422	0.459506029
Negr1	-0.0194424	0.019517501	0.34834849	0.459506029
Nrn1	-0.043322	0.043655656	0.35008639	0.459506029
Ppa2	-0.0514452	0.051815016	0.34985408	0.459506029
Suco	-0.0618724	0.062221891	0.34915757	0.459506029
Ubr4	-0.0515136	0.05190489	0.35003813	0.459506029
Kpnb1	0.0199248	0.020154709	0.35181589	0.460433708
Pgam5	-0.0232852	0.023536217	0.35147435	0.460433708
Slc27a4	-0.0208368	0.021075475	0.35177706	0.460433708
Rras	0.047347	0.048236218	0.35506269	0.464233066
Fn3k	-0.0402096	0.041061708	0.35613847	0.464571744
Rps13	0.02141568	0.021871375	0.35617698	0.464571744
Timm23	-0.0638228	0.065206169	0.35635363	0.464571744
Dpysl5	0.0384448	0.03935399	0.35723197	0.464619956
Grm5	-0.0238842	0.024437443	0.35701604	0.464619956
Syne1	-0.023083	0.023638775	0.35742263	0.464619956
Timm8a1	-0.0275382	0.028236289	0.35798721	0.464906401

38231	-0.0252682	0.025954328	0.35878764	0.465498299
Scamp5	-0.0242672	0.024957235	0.35935393	0.465785582
Gprin1	-0.0213884	0.022067538	0.36081899	0.467130628
Hint3	-0.0245096	0.025327487	0.36153067	0.467130628
Nipsnap1	-0.0244946	0.025312101	0.36153347	0.467130628
Tmem9b	-0.0371267	0.038386184	0.3617751	0.467130628
Cntnap2	0.0320862	0.033329131	0.36388702	0.467918828
Efr3a	-0.0620408	0.06447512	0.36410588	0.467918828
Sptb	-0.0202719	0.021029127	0.36328106	0.467918828
Tpm3;Tpm2	0.0359542	0.037317263	0.36352528	0.467918828
Tspan2	-0.077585	0.080631319	0.36411777	0.467918828
Ephx2	0.0485264	0.050482632	0.36457604	0.468062385
Acat1	-0.0139104	0.014596078	0.36848194	0.468176877
Cdo1	-0.0301944	0.031489403	0.36570027	0.468176877
Coa3	-0.0348876	0.036363589	0.36544608	0.468176877
Mapk1	0.0366834	0.038531473	0.36895211	0.468176877
Mrpl15	0.054111	0.056554868	0.36669067	0.468176877
Myo16	-0.0298299	0.031118242	0.36583071	0.468176877
Ndufb2	-0.03366	0.035372882	0.36917154	0.468176877
Pitpna	0.02747878	0.028803529	0.36801297	0.468176877
Pvrl1	-0.0504912	0.052785606	0.3668114	0.468176877
Rpl22	0.01647556	0.017295292	0.36868191	0.468176877
Srgap3	-0.0348559	0.036465259	0.36712864	0.468176877
Stmn1	0.028293	0.029646252	0.36784824	0.468176877
Uqcc2	-0.0546928	0.057328213	0.3680025	0.468176877
Acsl4	0.1006892	0.106273881	0.37114572	0.468907736
Arf5	-0.0455243	0.048053966	0.37119015	0.468907736
Crmp1	0.0323988	0.034148853	0.37052207	0.468907736
Prdx3	0.0540658	0.057057245	0.37108771	0.468907736
Slc25a46	-0.0293655	0.031017327	0.37148375	0.468907736
Slc39a10	0.03731422	0.039478145	0.37223162	0.469413034
Cbr3	0.0395914	0.042163273	0.37521088	0.469509469
Chid1	0.0392488	0.041887381	0.37617532	0.469509469
Cnnm2	0.0859692	0.09181023	0.37647961	0.469509469
Esd	0.0760882	0.080854411	0.37422148	0.469509469
Fasn	-0.0145512	0.015510655	0.37562649	0.469509469
Lactb	-0.0284897	0.030387271	0.37591045	0.469509469
Mrps26	0.0361482	0.038285546	0.37271849	0.469509469
Pdha1	-0.0250734	0.026582786	0.37317741	0.469509469
Rab23	0.0507462	0.053950776	0.37443855	0.469509469
Rps28	0.0561304	0.05955488	0.37352431	0.469509469
Tomm70a	-0.0226376	0.024152374	0.3760423	0.469509469
Tuba4a	0.024519	0.026141913	0.37573435	0.469509469
Stub1	0.02363198	0.025329523	0.3781279	0.471119487
Trappc5	-0.053749	0.057653192	0.37846825	0.471119487
Fam213a	0.03306418	0.035502938	0.37894164	0.471274415
Ank1	-0.0477872	0.05157752	0.38128107	0.471731388
Ap2a2	-0.0294094	0.03188516	0.38331799	0.471731388
Arl6ip5	-0.0198676	0.021548829	0.38350034	0.471731388
Bcl2l13	-0.0181421	0.019610967	0.38197255	0.471731388
Cct2	0.0262886	0.028340431	0.38074924	0.471731388
Cct5	0.0278607	0.030027803	0.38063715	0.471731388
Cend1	-0.0280644	0.03031871	0.38170458	0.471731388
Dnajc11	-0.025955	0.028009955	0.38122112	0.471731388
Ensa	0.04947388	0.053606069	0.38304232	0.471731388
Map1a	0.0595946	0.064478236	0.3823837	0.471731388
Ncald	-0.01756	0.018970854	0.3817117	0.471731388
Ywhae	0.0397354	0.043035277	0.38284324	0.471731388
Stmn2	0.0479346	0.052039794	0.38392668	0.471826102
Hagh	0.04038586	0.043958522	0.38510133	0.472839435
Map1lc3b;Map1lc3a	0.03280593	0.035749824	0.38563022	0.473058764
Shisa4	-0.0544376	0.059493609	0.38693179	0.473794767
Ube2v1	0.02749242	0.03004464	0.38691377	0.473794767
Gng12	-0.0254036	0.027846178	0.38828396	0.475019826
Dync1li2	-0.0294574	0.032342589	0.38902282	0.475174918
Ganab	0.0137096	0.01505543	0.38911438	0.475174918

Mt3	0.05037055	0.055416573	0.38994098	0.475754177
Strn3	-0.0311986	0.034378338	0.39065554	0.476195821
Dusp3	-0.0432866	0.047756094	0.39120169	0.476431566
Nckipsd	-0.0393697	0.043507358	0.39195586	0.47692
Ndufb5	-0.0460816	0.051159579	0.39403234	0.478584289
Osbpl1a	0.0271666	0.030144051	0.39379025	0.478584289
Actb	0.0202548	0.022539671	0.39509167	0.479439783
Acot7	-0.1306405	0.148115856	0.40349065	0.480080005
Agap2	0.0094944	0.010631408	0.39789691	0.480080005
Atp1b2	-0.019432	0.02185045	0.39978311	0.480080005
Cdipt	0.0299869	0.034036455	0.40399635	0.480080005
Ckap5	0.03304886	0.037262938	0.40100312	0.480080005
Cpne4	-0.0389124	0.043647744	0.39867507	0.480080005
Dclk1	-0.0184934	0.020998013	0.40415013	0.480080005
Enah	0.03032086	0.034106245	0.39993802	0.480080005
Gpi	-0.0193346	0.021572505	0.39628088	0.480080005
Iba57	-0.0536662	0.060717289	0.40254665	0.480080005
L1cam	-0.0291286	0.032620217	0.39794251	0.480080005
Lxn	-0.0553264	0.062532272	0.40209151	0.480080005
Mapk15	0.0369702	0.041700652	0.40117963	0.480080005
Mff	-0.0183619	0.020626709	0.39933644	0.480080005
Mrps16	0.07396274	0.083478046	0.40145738	0.480080005
Ncam2	-0.0216644	0.02423212	0.39740064	0.480080005
Oat	-0.0220018	0.024888658	0.402476	0.480080005
Pdcd6	0.0305406	0.034345112	0.39982921	0.480080005
Rab18	0.0202724	0.022983061	0.40346878	0.480080005
Rab5b	0.03776614	0.042492749	0.40006218	0.480080005
Sipa1l1	-0.0204867	0.02324739	0.40388289	0.480080005
Tecr	0.03371374	0.037770018	0.39812192	0.480080005
Tubb2b	0.0438156	0.049378549	0.40078454	0.480080005
Wnk2	0.0231992	0.026268886	0.40291621	0.480080005
Syncrip	-0.0410111	0.046670361	0.40516187	0.480858903
Lpcat4	-0.0870746	0.099434006	0.40671549	0.482278992
Pip4k2c	0.0198502	0.022826386	0.40984178	0.485559806
Cisd2	0.030014	0.034653707	0.41164959	0.48642156
Plscr3	-0.035377	0.040840731	0.41159462	0.48642156
Ppp1r1a	0.03375156	0.038956496	0.41150551	0.48642156
D10Jhu81e	-0.0208864	0.024171603	0.41269625	0.48680653
Eprs	0.0238822	0.027631701	0.41258444	0.48680653
Calml3	-0.0744318	0.086441225	0.41426161	0.486951844
Gsk3a	-0.0254446	0.029527544	0.41392128	0.486951844
Lppr3	0.0405082	0.047008734	0.4139253	0.486951844
Mcat	0.0342454	0.039761395	0.41415559	0.486951844
Psma3	-0.042337	0.049233952	0.41486066	0.487231958
Lrpprc	-0.0161952	0.018875836	0.41586366	0.487561994
Scamp1	-0.03984	0.046404317	0.41557491	0.487561994
Mtmr1	0.010701	0.012499916	0.41685336	0.488298464
Evl	0.0365534	0.042825502	0.41818052	0.488971598
Sh3glb1	0.0485378	0.056874681	0.41824645	0.488971598
Shank3	-0.0210928	0.024730552	0.41851412	0.488971598
Aldoa	0.0198354	0.023275737	0.41888666	0.488983858
Ap2m1	-0.024318	0.028559824	0.41926148	0.488998762
Dbt	0.02956488	0.034804132	0.42031513	0.489804691
Map6d1	-0.0401264	0.047298678	0.4208936	0.489978173
Pygb	-0.030812	0.036343553	0.42118956	0.489978173
Lyrm4	-0.0302655	0.035771562	0.42209375	0.490185614
Vps11	0.0706362	0.083466617	0.4219869	0.490185614
Lypla1	-0.0310809	0.036794208	0.42280681	0.490591875
Hsp90aa1	-0.0299428	0.035586612	0.42455549	0.491728405
Mboat7	-0.0246258	0.029312736	0.42524262	0.491728405
Ppp1r21	-0.0356826	0.042456638	0.4250617	0.491728405
Rpl8	0.0211624	0.025185462	0.42515978	0.491728405
Cpt1c	-0.0342407	0.040911484	0.42691563	0.492819116
Mrpl47	-0.0481598	0.057532527	0.42683999	0.492819116
Myo5a	-0.0131948	0.015828361	0.42868234	0.493789025
Pacs1	0.0174016	0.020882824	0.42885264	0.493789025

Ppid	0.039594	0.047493629	0.42865462	0.493789025
Gnl1	0.01201352	0.014435353	0.42942096	0.494022234
Emc10	0.0484634	0.058467104	0.4311953	0.495219865
Lrpap1	0.03653612	0.044068199	0.43109869	0.495219865
40057	-0.0201546	0.02434975	0.4318295	0.495251775
Ap1m1	-0.0428544	0.052005511	0.43379741	0.495251775
Atp6v1g2	-0.0121266	0.014713012	0.43370445	0.495251775
Jam3	-0.0577157	0.070123411	0.43432123	0.495251775
Krt76;Krt4	-0.2902229	0.352032726	0.43359138	0.495251775
Nfu1	0.01644366	0.019926651	0.43316861	0.495251775
Ngef	-0.0266092	0.032344438	0.43452327	0.495251775
Pdia6	0.0262794	0.031860123	0.43336821	0.495251775
Ube2o	-0.0623866	0.075396811	0.43197343	0.495251775
Aldh4a1	0.0315794	0.038467161	0.43545665	0.495331839
Atl1	0.0206076	0.025136695	0.43606049	0.495331839
Myl6	0.0243384	0.029664074	0.43571316	0.495331839
Slc32a1	-0.0167812	0.020419457	0.43498408	0.495331839
Fxyd6	0.0497995	0.061086533	0.43853591	0.496634408
Hpca	0.0140056	0.017174724	0.43840167	0.496634408
Pccb	0.0432845	0.053087507	0.43847454	0.496634408
Rab3c	-0.0494507	0.060678235	0.43867804	0.496634408
Nutf2	0.0206136	0.025446803	0.44133001	0.49921829
Idh2	0.024594	0.030398337	0.44187754	0.499419364
Rps3	0.04356432	0.0540759	0.44374942	0.501115658
Diras2	-0.0288696	0.036105342	0.44703507	0.504404328
Spcs2	0.0617212	0.077276054	0.44751905	0.504528925
Acsbg1	0.02902947	0.036444093	0.44870436	0.505203475
Ppp2ca	0.0103974	0.013057889	0.44886549	0.505203475
Acadm	-0.0170264	0.021453249	0.45029631	0.505921336
Aldh1a1;Aldh1a7	0.0464256	0.058540705	0.45062706	0.505921336
Wasf3	-0.0190246	0.023955296	0.45001056	0.505921336
Gps1	0.0364988	0.046120287	0.45154397	0.506529706
Arhgef7	-0.0394882	0.050067888	0.45302742	0.507351025
Csnk1g1;Csnk1g2	0.03954166	0.050120783	0.45289808	0.507351025
Plcg1	0.0353048	0.044851219	0.45387801	0.507882481
Kiaa1671	-0.034599	0.044003811	0.45436516	0.508006704
Rheb	-0.0588575	0.075160247	0.45612644	0.509554105
Cspg5	-0.0693503	0.088717726	0.45690215	0.509577695
Csrp1	0.0246648	0.031532199	0.45661671	0.509577695
Pafah1b2	0.04135684	0.053024268	0.45786649	0.510231887
Ap1s1	-0.0613888	0.078815878	0.45846283	0.510475249
Psma1	0.0170752	0.022004949	0.46008887	0.511863776
Apool	0.04456992	0.057660387	0.46176354	0.513304078
Prkca	0.0274102	0.035555789	0.46292008	0.514166528
Slc6a1	-0.0284734	0.036975546	0.46339448	0.514270521
Lgi2	-0.0547072	0.071123693	0.46388596	0.514393296
Chl1	-0.0576818	0.075207486	0.46513001	0.514505542
Igsf21	-0.0277126	0.036080472	0.46450649	0.514505542
Snph	0.0224672	0.029289707	0.46507395	0.514505542
Gphn	0.015881	0.020745408	0.46594513	0.514985413
Anxa6	0.02534936	0.033312643	0.46851875	0.516322311
Dynlrb1	0.01916502	0.025192424	0.46863609	0.516322311
M6pr	-0.0406838	0.053459998	0.46848445	0.516322311
Nap1l4	-0.0507188	0.066677292	0.46868387	0.516322311
Nsf	-0.016595	0.021855502	0.46944987	0.516744688
Anxa5	-0.0205578	0.027179034	0.47110344	0.517720962
Nras	-0.0133651	0.017661096	0.47089484	0.517720962
Cat	-0.0237977	0.031641887	0.47353849	0.517966603
Dld	-0.0153258	0.020381731	0.47362799	0.517966603
Dnajb12	-0.0294034	0.038968385	0.47214679	0.517966603
Mccc2	0.02031546	0.026983609	0.47309162	0.517966603
Praf2	-0.0191216	0.025393292	0.47301462	0.517966603
Rufy3	-0.0184112	0.024384341	0.47186478	0.517966603
Clip3	0.0972078	0.129636229	0.47481631	0.518438739
Gpc5	0.059427	0.07929718	0.47506088	0.518438739
Stxbp5	-0.0142939	0.01907994	0.47521127	0.518438739

Mapk3	0.05304942	0.071046693	0.47662267	0.519558849
Abhd16a	-0.0240294	0.032393107	0.47941394	0.520162231
Cops2	-0.0273555	0.036841264	0.47900244	0.520162231
Lancl2	-0.0255248	0.034325269	0.4783775	0.520162231
Nln	0.01342115	0.018095636	0.47948697	0.520162231
Nptx1	0.0286186	0.03857158	0.47932519	0.520162231
Rps4x	0.00939314	0.012627624	0.47823927	0.520162231
Ndufb8	-0.0228506	0.03084475	0.47997586	0.520226023
Vat1	0.03183984	0.043013246	0.48031613	0.520226023
Dynll2	-0.0299276	0.040526861	0.48133286	0.520909509
Eif5	0.031943	0.043346963	0.48222375	0.521455815
Arl15	0.021701	0.029529958	0.48339499	0.521469818
GOLGA7B	-0.0234293	0.031849581	0.48296703	0.521469818
Ptpra	0.01862674	0.02533962	0.48327811	0.521469818
Ruvbl1	-0.0558568	0.076145199	0.48415879	0.521876942
Ccny	0.02968018	0.040646784	0.48609943	0.523550928
Ccdc177	-0.014811	0.020351764	0.48751655	0.52424112
Pdlim5	-0.0296678	0.040750483	0.48735118	0.52424112
Ndufaf3	-0.0520586	0.071640293	0.48814492	0.524408439
Srr	0.0180836	0.024903638	0.4884487	0.524408439
Rpl13	-0.0124818	0.017257476	0.4901182	0.525782898
Gmfb	0.0379228	0.052499343	0.49065407	0.525865922
Vps35	0.0288352	0.039949129	0.4909743	0.525865922
Fkbp8	-0.0154528	0.021485599	0.49248005	0.526226749
Gpx1	0.03180086	0.044182434	0.49216034	0.526226749
Rala	-0.0128018	0.017777285	0.49195119	0.526226749
Hgs	-0.0219852	0.030739983	0.49482624	0.528311004
Rgs10	-0.0427204	0.059787434	0.49521296	0.528311004
Coro1a	0.0229516	0.032186004	0.49605974	0.528416201
Tomm20	-0.0252016	0.035344205	0.49609405	0.528416201
Suox	-0.0274571	0.03861441	0.49725318	0.529233471
Cops8	0.03673962	0.051878113	0.49893775	0.530034729
Cpne6	0.0460956	0.065167134	0.49943608	0.530034729
Rpl12	0.03487614	0.049322296	0.49957578	0.530034729
Vamp1	0.0355082	0.050210372	0.49952776	0.530034729
Tmed9	-0.028551	0.040469661	0.50052748	0.530627619
Ddx1	-0.0341872	0.048795345	0.50340169	0.533256112
Hepacam	-0.0174832	0.025005429	0.5042588	0.533675934
Pls3;Lcp1	0.04074116	0.058316629	0.50458828	0.533675934
Rab27b	0.0488828	0.070209738	0.50599981	0.533914529
Rpl7a	0.02085254	0.029938041	0.50583151	0.533914529
Sfn	-0.0214798	0.030801202	0.50532983	0.533914529
Grm1	-0.0307385	0.044236366	0.50681314	0.53435527
Cacna1e	0.0253217	0.036489608	0.50736417	0.534518969
Pik3r2	-0.0458018	0.066174207	0.50843681	0.535231525
Rab35	-0.0139832	0.02023329	0.50905681	0.535466845
Sdr39u1	0.02454307	0.035580765	0.50983993	0.535470753
Wdr37	0.0223985	0.032472787	0.50985346	0.535470753
Ppl	0.0402602	0.058450761	0.51043443	0.535664379
Ca4	0.0282022	0.041043949	0.51142944	0.535875823
Psmb4	-0.0647814	0.094227511	0.5112037	0.535875823
Rasgrf1	0.0697104	0.10187209	0.51312127	0.537231741
Exoc1	-0.0215892	0.03167006	0.51468005	0.537481281
Gbas	-0.0178714	0.026209827	0.51458028	0.537481281
Map6	0.007964	0.011679808	0.5145792	0.537481281
Tmod2	0.0161986	0.023778107	0.51495142	0.537481281
Atp9a	0.02383412	0.035248909	0.51800169	0.537986144
Cst3	0.02140028	0.031565171	0.51691523	0.537986144
Ddah1	-0.018582	0.027550264	0.51902007	0.537986144
Oxct1	0.0144246	0.02138148	0.51892705	0.537986144
Ppp2r4	-0.0255154	0.037684688	0.51745388	0.537986144
Rock2	-0.0131126	0.019428329	0.51875157	0.537986144
Vapb	0.0173938	0.025669965	0.51714253	0.537986144
Vcan	0.037984	0.056158414	0.51787751	0.537986144
Zc2hc1a	-0.0158536	0.023372863	0.51672405	0.537986144
Mkl2	0.02805368	0.041758813	0.52063383	0.538831815

Vsnl1	-0.0402978	0.059975006	0.52056903	0.538831815
Mmab	0.0455136	0.067818558	0.52105339	0.538853124
Tanc2	0.0181522	0.027112601	0.52201912	0.539438794
Cmc1	-0.0634758	0.095361877	0.5243708	0.541454683
Cntln	0.02910056	0.043846359	0.52554695	0.542254577
Capn2	0.03926432	0.059402933	0.52719588	0.54292574
Cpt2	0.0787588	0.119432789	0.52813549	0.54292574
Eef1g	0.029852	0.045173269	0.52728695	0.54292574
Kif2a	-0.0162924	0.024684678	0.52778182	0.54292574
Nefh	-0.051651	0.078339513	0.52820735	0.54292574
Map2k4	0.02229	0.033858359	0.52881201	0.543133902
Atp1a2	-0.0172996	0.026341222	0.52977669	0.543298399
Cntn1	0.0097804	0.014886828	0.52963418	0.543298399
Anxa7	0.0294514	0.044942358	0.53065347	0.543784659
Cand1	0.0222862	0.03422962	0.53324627	0.543965305
Nadk2	0.0314916	0.048332146	0.53294779	0.543965305
Pef1	0.0202914	0.03102696	0.53146217	0.543965305
Pgm1	0.02541092	0.039002464	0.53297564	0.543965305
Rab15	0.02912786	0.044696927	0.53288133	0.543965305
Vdac3	-0.0233916	0.035794134	0.53176127	0.543965305
Rras2	-0.0114337	0.017580121	0.53367649	0.543993302
Arhgap44;Arhgap17	0.01889174	0.029347442	0.53776519	0.545280314
Asna1	0.01623467	0.02519551	0.53738266	0.545280314
Dgkb	0.0403324	0.062347706	0.53581543	0.545280314
Eif4a1	-0.029845	0.046323173	0.53742574	0.545280314
Sec31a	-0.021422	0.033180587	0.53660045	0.545280314
Tbc1d10b	-0.0159148	0.024589698	0.53561942	0.545280314
Ubxn6	-0.0185584	0.028794323	0.53727918	0.545280314
Atp6v1a	-0.0098302	0.015353165	0.53989668	0.546326918
Eps15l1	0.01897808	0.029762945	0.54152297	0.546326918
Hsd17b10	-0.0169365	0.026433639	0.53962231	0.546326918
Rapgef5	-0.0550442	0.086231554	0.54109678	0.546326918
Rpl18	-0.0281373	0.044139048	0.54162889	0.546326918
Shmt2	-0.0176134	0.027627759	0.54159431	0.546326918
Tars	0.0575807	0.090075306	0.54052764	0.546326918
Rpl9	-0.0226702	0.035609145	0.54214278	0.546437178
Prdx5	0.0164112	0.025811	0.54264934	0.546539884
Akap5	-0.0320028	0.050909995	0.54712498	0.546968799
Dbn1	0.021015	0.033140236	0.54370222	0.546968799
Dmtn	-0.0223546	0.035478227	0.54620516	0.546968799
Exoc4	-0.0204499	0.03243197	0.54592213	0.546968799
Gcdh	0.02493266	0.039475839	0.54527309	0.546968799
Mast1;Mast3	-0.0306835	0.048786065	0.5469232	0.546968799
Mt1	0.0404918	0.064361391	0.54680345	0.546968799
Nfs1	-0.0125149	0.019898293	0.54692135	0.546968799
Rap1gap	-0.044722	0.070629537	0.54428062	0.546968799
Syp	0.0290404	0.046154604	0.54676206	0.546968799
Necap1	-0.0135604	0.021596257	0.5475672	0.547006002
Aco2	0.0118918	0.018960377	0.54801138	0.547045101
Fam131b	0.028067	0.044802576	0.54846803	0.547096595
Acsl1	0.01048304	0.016868288	0.55158752	0.547232963
Asah1	0.0201218	0.032399593	0.55184613	0.547232963
Efhd2	-0.021218	0.034084004	0.55092693	0.547232963
Mark3	0.01843932	0.029670699	0.55158672	0.547232963
Rab8a	0.012934	0.020817907	0.55169532	0.547232963
Sh3glb2	0.0185556	0.029824583	0.55115379	0.547232963
Tceb2	0.01761136	0.028280518	0.55079099	0.547232963
Tubb4a	0.0297604	0.047756099	0.55051814	0.547232963
Add2	0.0175458	0.028411735	0.55403641	0.547314362
Apmap	0.0346252	0.056143448	0.55455543	0.547314362
Dnaja1	-0.0143784	0.023303886	0.55438752	0.547314362
Higd1a	0.02684464	0.043551129	0.55476485	0.547314362
Mthfd1l	0.0099234	0.016002729	0.55243718	0.547314362
Prkar2a	0.0203644	0.032982577	0.55411522	0.547314362
Reps1	0.0264776	0.042780491	0.55318199	0.547314362
Cdip1	0.03247	0.052898099	0.55638055	0.547944505

Crat	-0.0178485	0.02912642	0.55702637	0.547944505
Ehd3	0.02305734	0.03760565	0.55681238	0.547944505
Rpl27a	0.0298848	0.048658566	0.5561594	0.547944505
Dhrs7b	-0.0322898	0.052749813	0.55744406	0.547956292
Palm2	0.01006392	0.016494984	0.55871071	0.548403118
Pdcd6ip	-0.0296318	0.04851778	0.55831939	0.548403118
Pik3r4	-0.019561	0.032122981	0.55945409	0.548734002
Ddost	-0.0242066	0.039987779	0.5617218	0.55011086
Hpcal4	-0.0501817	0.082955182	0.56199052	0.55011086
Slc12a2	0.0390628	0.064589687	0.56207976	0.55011086
Cmtm4	0.05725539	0.09523049	0.56433029	0.551789913
Gnb1	-0.0079944	0.0133066	0.56461244	0.551789913
Akr1b1	0.01364552	0.022826262	0.56650732	0.551949621
Eva1a	0.0287534	0.048118925	0.56666647	0.551949621
Fahd2	0.01426382	0.023896292	0.56707545	0.551949621
Mark2	-0.0214382	0.035930018	0.56722785	0.551949621
Pfn1	0.022588	0.037733279	0.5659842	0.551949621
Rpl23a	-0.0170005	0.028365724	0.56553363	0.551949621
Sv2b	0.0174034	0.029221781	0.56792928	0.552234292
Slc22a23	-0.015654	0.026436791	0.57011604	0.553165879
Sod2	-0.0169946	0.028679518	0.5698356	0.553165879
Stx1b	-0.0072846	0.01229072	0.56975793	0.553165879
Ap3d1	0.01714998	0.029086053	0.57171156	0.553918096
Mtco2	-0.0226882	0.038449389	0.57142391	0.553918096
Myh9	0.0210938	0.036062485	0.57472123	0.556434934
Acss1	0.03733602	0.063953677	0.5754426	0.556677103
Rabggta	-0.031836	0.054584064	0.57579569	0.556677103
Psmd3	-0.0271458	0.046601974	0.57627284	0.556739888
Flot2	-0.0114024	0.019649148	0.57768728	0.557584675
Pabpc1	0.02063485	0.035586208	0.57797294	0.557584675
Pdia4	0.0136816	0.023681461	0.57933665	0.558501344
Csnk2a1	-0.03141	0.054950995	0.58329143	0.561112372
Mlec	0.0202722	0.035459167	0.58322343	0.561112372
Rltpr	-0.0179946	0.031443761	0.58285316	0.561112372
Pcp4	0.0147481	0.025870118	0.58427223	0.561655837
Isoc2a	0.02318894	0.040745776	0.58489935	0.561858784
Pfkp	-0.0109382	0.019251062	0.58549849	0.562034577
Rab11b;Rab11a	-0.0212026	0.037475996	0.58706735	0.563104049
Rpl11	0.02170032	0.038395407	0.58744645	0.563104049
Dnajc6	0.0126694	0.02249758	0.58876697	0.563170757
Fah	-0.0130602	0.023166876	0.5883789	0.563170757
Xkr4	-0.0200952	0.035622792	0.58814013	0.563170757
Pak2	-0.0157496	0.028121178	0.59077063	0.56428804
Rps12	0.0231194	0.041235906	0.59038042	0.56428804
Ppt1	0.0362174	0.064756717	0.59127671	0.564372303
Cltb	0.0209044	0.037539471	0.59285249	0.564412528
Cyb5b	0.02454026	0.043940455	0.59179428	0.564412528
Psmb5	-0.0146675	0.026334886	0.59278924	0.564412528
Synpo	-0.0138114	0.024811512	0.59299043	0.564412528
Cacna2d1	0.0107054	0.019346962	0.59515366	0.564948958
Nedd4l	-0.037657	0.067860193	0.59412037	0.564948958
Stx6	0.01283638	0.023202822	0.59522718	0.564948958
Tmem106b	-0.0397168	0.07169511	0.59474192	0.564948958
Eci2;Eci3	-0.0151754	0.02754405	0.59671401	0.565095162
Gnb4	0.04055658	0.073567108	0.59649382	0.565095162
Kiaa1045	-0.0072364	0.013146831	0.59705482	0.565095162
Rab3a	-0.0094604	0.017134899	0.59595376	0.565095162
Lrrc4c	-0.0512356	0.093402972	0.59828924	0.565470973
Pcca	0.0157818	0.028757192	0.59812464	0.565470973
Tdrkh	0.0262452	0.047935405	0.59896526	0.565714034
Ablim1	-0.0178984	0.032769831	0.5998342	0.566138838
Ptcd3	0.025302	0.046485898	0.60107618	0.566914875
Hba	0.1610544	0.29692832	0.60231958	0.567433405
Synj2bp	0.01255347	0.023153754	0.60246621	0.567433405
Usp9x	0.0160556	0.029714965	0.60368862	0.568188508
Cyb5a	-0.013871	0.025736932	0.60458888	0.568639561

Cacnb3	-0.0302284	0.056559612	0.60755758	0.571034082
Ywhag	0.0153328	0.028752286	0.60833712	0.571369149
Glrx	0.0107946	0.020339455	0.61002378	0.572154391
Hadha	0.0166728	0.031420519	0.61008206	0.572154391
Hspa12a	-0.0092474	0.017445049	0.61044404	0.572154391
Adcyap1r1	-0.0158118	0.029866584	0.61088971	0.572175032
Cadm3	0.0136164	0.025865904	0.61287303	0.573196243
Hadh	0.0257782	0.049021978	0.6132532	0.573196243
Rtn3	0.014372	0.027266992	0.61243423	0.573196243
Txnrd2	-0.015508	0.02953859	0.61381201	0.573321789
Nudc	0.0189895	0.036477441	0.61675489	0.575672427
Me1	0.016983	0.032794653	0.61856921	0.576568971
Sfxn1	-0.010692	0.020642064	0.6184941	0.576568971
Creld1	0.01738576	0.033701241	0.61989003	0.576599509
Dag1	0.0151432	0.029294329	0.61918677	0.576599509
Denr	0.01982622	0.038445002	0.62000717	0.576599509
Epb41l1	-0.0109532	0.021258013	0.62030963	0.576599509
Capn5	-0.0112844	0.022034719	0.62240235	0.576956443
Gls	-0.0130412	0.025445356	0.62213563	0.576956443
Plxnb2	-0.0219734	0.042824355	0.6217429	0.576956443
Ppp2r1a	0.0135826	0.026468583	0.62170674	0.576956443
Bcat1	0.01057974	0.020783325	0.62445739	0.577151506
Cds2	-0.015259	0.03000108	0.6247494	0.577151506
Fam171a2	-0.0098796	0.019365975	0.62371854	0.577151506
Iscu	-0.0110424	0.02169724	0.62453669	0.577151506
Slc25a23	0.00941935	0.018437695	0.62323493	0.577151506
Atp6v1h	0.0077146	0.01522617	0.6260547	0.577819803
Pi4ka	-0.0070776	0.013980192	0.62632845	0.577819803
Chchd2	0.02061094	0.040842688	0.62741411	0.578426274
Coq7	-0.0190465	0.037855896	0.62842855	0.578966306
Cadm1	0.02566312	0.051688171	0.63289592	0.580292014
Cnbp	0.0279614	0.056242392	0.63245079	0.580292014
Dbnl	0.02419564	0.048771317	0.63316239	0.580292014
Dctn1	0.008496	0.017103471	0.63273242	0.580292014
Ehd1	-0.0289888	0.057981436	0.63055835	0.580292014
Fam171b	0.0150832	0.030416234	0.63330471	0.580292014
Mecr	-0.0154296	0.031078958	0.63291824	0.580292014
Suclg2	-0.0219537	0.044267015	0.63327371	0.580292014
Cep170b	-0.0109005	0.022020599	0.63389952	0.580443238
Yme1l1	0.0354266	0.071753842	0.63476941	0.580845985
Gnai3	-0.0211347	0.042943097	0.63583006	0.581422615
Hsph1	-0.01075	0.02190832	0.63682715	0.581940376
Nrgn	0.02111454	0.043127698	0.63757134	0.582226503
Arpc2	-0.0210412	0.0432675	0.63979238	0.582533099
Csnk1e	-0.0148017	0.030502014	0.64049494	0.582533099
Dnajb11	-0.0378565	0.077953628	0.64025123	0.582533099
Fkbp2	0.01385542	0.028510961	0.64002086	0.582533099
Grpel1	0.02001244	0.041030625	0.63881657	0.582533099
Mut	0.01045876	0.021470472	0.63923759	0.582533099
Cct8	0.0137716	0.028432027	0.64110617	0.582696627
Add3	-0.0139675	0.028953489	0.64243576	0.582803878
Mp68	0.03237592	0.067368843	0.64368393	0.582803878
Myadm	-0.0167589	0.034911213	0.64404743	0.582803878
Nmral1	-0.0234654	0.048911417	0.64424474	0.582803878
Ociad1	-0.0211796	0.043815903	0.64177982	0.582803878
Slc6a9	0.02455112	0.050961096	0.6428779	0.582803878
Snap91	-0.0162598	0.033763512	0.64300232	0.582803878
Ipo5	-0.0144602	0.030187472	0.64474796	0.582868703
Eif4b	-0.011083	0.023213742	0.64582519	0.583164139
Slmap	-0.0216636	0.045391012	0.64593832	0.583164139
Fxn	-0.0200784	0.042222666	0.64711849	0.583839357
Strn4	0.02006966	0.042294831	0.6478132	0.584075962
Arhgap39	-0.0088808	0.01897734	0.65229129	0.585047619
Cd81	-0.0141637	0.030220867	0.65180816	0.585047619
Cnrip1	0.0135938	0.02879407	0.64946191	0.585047619
Gfm1	-0.015042	0.032104469	0.65190489	0.585047619

Maob	0.0124928	0.026623249	0.65141808	0.585047619
Mtx2	-0.015873	0.033924322	0.65234174	0.585047619
Prkcb	-0.0149398	0.03187886	0.65182961	0.585047619
Slc7a5	-0.01282	0.027400595	0.65235626	0.585047619
Acsf2	-0.0254713	0.054891392	0.65499122	0.58593602
Adh5	0.00954418	0.020517652	0.65420998	0.58593602
Cct3	-0.0119324	0.025722036	0.65508218	0.58593602
Echs1	0.0108996	0.023455913	0.65454245	0.58593602
Rpl31	0.0226446	0.049068969	0.65673916	0.586641089
Rpsa	0.015433	0.033426102	0.65658806	0.586641089
Abhd11	0.016394	0.035595005	0.65736799	0.586814693
Mrps34	-0.0135924	0.029556917	0.65784806	0.586855364
Gna11	-0.0079812	0.017396729	0.65860212	0.587140239
Acly	-0.0128208	0.028083985	0.66015775	0.587320812
Dpp3	0.0165002	0.036288587	0.66141379	0.587320812
Rab31	0.0162207	0.035609094	0.66084344	0.587320812
Rasgrf2	0.0204074	0.044873539	0.66135733	0.587320812
Rnasek	0.0140844	0.0308322	0.65995679	0.587320812
Rpl37	-0.0214196	0.046845457	0.65965962	0.587320812
Cdh2	-0.0158486	0.035000853	0.66271607	0.587966354
Cdv3	-0.0124611	0.02762275	0.6638821	0.587966354
Coq6	0.0187401	0.041425977	0.66301324	0.587966354
Napg	0.0100322	0.022228223	0.66373716	0.587966354
Rcn2	0.0245766	0.054707675	0.6651829	0.588346796
Trim9	-0.0291742	0.064879157	0.66488205	0.588346796
Ap3m2	-0.0222314	0.04961725	0.66599693	0.588681285
Sept3	0.0135038	0.03025006	0.66714031	0.589306259
Abhd10	0.01932164	0.043370726	0.66776782	0.589475028
Alcam	0.010542	0.023723226	0.66854721	0.58977756
Reep5	-0.0164526	0.037198251	0.66998844	0.590663184
Adrbk1	-0.0095612	0.021786096	0.67236921	0.591365281
Atpaf1	-0.022757	0.052059945	0.67357628	0.591365281
Bri3bp	0.0183334	0.042032745	0.67424493	0.591365281
Cttn	-0.0176296	0.040529085	0.67506833	0.591365281
Efnb2	0.036216	0.082815425	0.6734517	0.591365281
Elfn2	0.01802964	0.04130007	0.6739786	0.591365281
Me3	-0.0121904	0.02797888	0.67457156	0.591365281
Psmd5	0.0234788	0.054071066	0.67560117	0.591365281
Syn2	0.0170626	0.039288926	0.67555654	0.591365281
Trnt1	0.026439	0.060383993	0.67307763	0.591365281
Tsg101	0.036579	0.083307069	0.67221682	0.591365281
Agpat3	0.0125915	0.029044428	0.67608577	0.591406176
Fxyd1	0.0186241	0.043142801	0.67736848	0.591688058
Hsd17b4	0.01595724	0.036958213	0.67731313	0.591688058
Syt7	0.01470016	0.034093046	0.67772228	0.591688058
Nudt17	-0.0221644	0.051580991	0.67875256	0.591822432
Rtn4rl2	-0.0185438	0.043108699	0.67842958	0.591822432
Abi1	-0.0081534	0.019058571	0.68007461	0.591828942
Gnai2	-0.009136	0.021354139	0.68005699	0.591828942
Paics	-0.010975	0.025631874	0.67981541	0.591828942
Chp1	-0.0171688	0.04031442	0.68142699	0.592454923
Madd	0.01722302	0.040535996	0.68211956	0.592454923
Ndufv3	-0.018943	0.044621737	0.68237006	0.592454923
Slc9a1	0.0278486	0.065639028	0.68254855	0.592454923
Dnajb2	-0.0087441	0.020652057	0.6831567	0.59260195
Cyb5r3	-0.0101972	0.024436877	0.68744038	0.593224348
Dync1h1	0.0117434	0.027867905	0.68456071	0.593224348
Gnas	-0.0126	0.030183888	0.68733352	0.593224348
Gpr158	0.0075934	0.018040804	0.68490668	0.593224348
Inpp4a	-0.0121724	0.029018341	0.68590878	0.593224348
Jagn1	-0.0098338	0.023597081	0.68782724	0.593224348
Lppr4	-0.0123046	0.029405705	0.6866308	0.593224348
Pdcd5	0.01246736	0.029787643	0.68656158	0.593224348
Rnf14	0.022668	0.054134844	0.68642767	0.593224348
Exoc7	-0.0166153	0.040098603	0.68949745	0.593803055
Hcn1	-0.0146538	0.035332173	0.68922809	0.593803055

Plxna4	-0.0071714	0.017326167	0.68981719	0.593803055
Yipf5	0.0269464	0.065213441	0.69031122	0.593849833
Ephb2	-0.0428774	0.103947539	0.69081232	0.59390263
Cadps	0.0159704	0.039079242	0.69350467	0.595838009
Ccdc127	-0.0137271	0.033694364	0.69439838	0.596226581
Actn1	-0.01091	0.027096764	0.69776419	0.596298168
Cfl1	0.0127046	0.03163076	0.69845527	0.596298168
Mtatp8	-0.0109996	0.027375276	0.69834502	0.596298168
Ppfia2	-0.0122597	0.030185956	0.69528824	0.596298168
Ppp1ca	-0.0128776	0.031932206	0.69730616	0.596298168
Ppp2r5c	0.037261	0.092381399	0.6972642	0.596298168
Rdh14	-0.0139502	0.034424207	0.69591855	0.596298168
Sparcl1	0.0397222	0.098146939	0.69628755	0.596298168
Tmem261	-0.0155918	0.038609911	0.69691797	0.596298168
Stt3b	-0.0161636	0.040368677	0.69934187	0.596677923
Cct4	0.0119242	0.029926101	0.70071801	0.596861263
Gpd1	0.0149376	0.037505406	0.7008428	0.596861263
Ndufb7	-0.0054702	0.013745766	0.70107173	0.596861263
Psmc1	0.0138034	0.034716938	0.70132442	0.596861263
Hsd17b8	0.011823	0.029852681	0.70242507	0.597421516
Mthfd1	0.00720824	0.018237656	0.70299598	0.597530805
Grcc10	-0.0152488	0.038644499	0.70345518	0.597545069
Shisa7	-0.0076437	0.019476739	0.70497251	0.598457559
Arhgap21	-0.0041735	0.010675335	0.70604388	0.598614553
Traf3	0.01502968	0.038396817	0.70569862	0.598614553
Me2	-0.020266	0.052056275	0.70720536	0.598847458
Rpl13a	-0.019322	0.049601068	0.70703577	0.598847458
Pfkl	0.01432588	0.036957031	0.70839513	0.598915545
St13	-0.0091545	0.023635057	0.70861608	0.598915545
Svop	-0.0250008	0.064439403	0.70815496	0.598915545
Clasp2	-0.009299	0.024114239	0.70982777	0.599472971
Cse1l	0.027013	0.070136039	0.71016331	0.599472971
Dos	0.0179609	0.046888566	0.71165673	0.599937507
Epb41l3	-0.0088808	0.023217259	0.71204621	0.599937507
Hbb-b1;Hbb-b2	0.10300286	0.268446948	0.71119775	0.599937507
Cops3	0.020959	0.055184215	0.71397758	0.600067432
Pdk3	-0.0118122	0.031032	0.71337456	0.600067432
Rps6	-0.0113537	0.029886374	0.71390986	0.600067432
Tmsb4x	0.01964971	0.051597624	0.71324615	0.600067432
Agpat1	0.0256702	0.06834733	0.71698901	0.600438939
Cacna2d3	0.030836	0.082226722	0.71739843	0.600438939
Flot1	-0.0069124	0.018559973	0.71924143	0.600438939
Gna13	0.0088348	0.023472814	0.71641732	0.600438939
Gss	-0.0156826	0.041876546	0.71776736	0.600438939
Micu3	0.01689696	0.045380421	0.71930985	0.600438939
Pex14	0.0169902	0.045030425	0.71575957	0.600438939
Rps11	-0.0124984	0.033167171	0.71609897	0.600438939
Slc44a2	0.00874934	0.023295114	0.71698731	0.600438939
Tceb1	0.0142984	0.038274061	0.71842308	0.600438939
Tln1	0.0159174	0.042548549	0.71805083	0.600438939
Pygm	0.02283376	0.062004178	0.72223204	0.602505837
Bai3	-0.0161639	0.044061491	0.72324582	0.60297912
Tomm34	-0.0100618	0.027724255	0.72606349	0.604954818
Irgq	0.0186784	0.051732206	0.72740395	0.605698029
Lrrc47	0.02008744	0.056040792	0.72928636	0.606891324
Glo1	0.00928814	0.026016083	0.73031456	0.607372732
Gpt2	0.0366002	0.103124306	0.73182918	0.607776882
Mtfp1	0.01180932	0.033315644	0.73215052	0.607776882
Rps2	-0.0158608	0.044694778	0.73186105	0.607776882
Cul5	0.01064578	0.030175124	0.73335345	0.607919405
Erlin2;Erlin1	0.01973863	0.0559939	0.73355981	0.607919405
Prkaca	0.0143518	0.040730772	0.73367252	0.607919405
Ggt7	0.00623647	0.017918078	0.73678035	0.609286206
Gstz1	-0.0121164	0.034977524	0.7379728	0.609286206
Nlrx1	-0.0151842	0.043467134	0.73586151	0.609286206
Rab1b	-0.0069941	0.020200773	0.73810081	0.609286206

Rab3gap1	0.0176064	0.050894399	0.7383095	0.609286206
Rps19	0.00861632	0.024923958	0.73847988	0.609286206
Txn	0.0117286	0.033742344	0.73711403	0.609286206
Tmeff1	-0.0458066	0.132968317	0.73935674	0.609637253
Cltc	0.0137964	0.040332476	0.74111339	0.610712855
Canx	-0.00784	0.023034933	0.74235565	0.61136353
Dnaja4	-0.0205296	0.060559735	0.74333829	0.611427132
Rgs7	-0.0066956	0.019741368	0.74321612	0.611427132
Gpx4	-0.00834	0.02481102	0.7454119	0.612014594
Rplp0	-0.0093583	0.027826258	0.7452862	0.612014594
Vps41	-0.0539848	0.160223717	0.74483566	0.612014594
P4htm	0.0426034	0.12708335	0.74606573	0.612179271
Actn2	-0.0120819	0.036333899	0.7480396	0.612723894
Cdh10	-0.0097116	0.029182405	0.74784527	0.612723894
Plgrkt	0.0243404	0.073214408	0.74809046	0.612723894
Bcr	-0.0116728	0.035364297	0.74982218	0.613480609
Lsamp	-0.0065632	0.019892418	0.7499228	0.613480609
Ak5	-0.0110845	0.034685295	0.75747711	0.614706102
Aldh9a1	-0.0097204	0.030995581	0.76184128	0.614706102
Cct6a	0.00933674	0.029839701	0.76236066	0.614706102
Cdc42ep4	0.023538	0.075316927	0.76263685	0.614706102
Coq9	0.0059322	0.018545984	0.75726503	0.614706102
Ephx1	0.0127266	0.039143059	0.75342226	0.614706102
Gnb5	-0.0059024	0.018383034	0.75637829	0.614706102
Ina	0.0249072	0.07867125	0.75965185	0.614706102
Islr2	-0.0201222	0.063732658	0.76028833	0.614706102
Kcna2	0.0174282	0.05444301	0.75707963	0.614706102
Lonp1	-0.0079808	0.025025297	0.75796275	0.614706102
Mfn2	-0.0112641	0.036068328	0.76279912	0.614706102
Mrpl9	0.0118204	0.036356753	0.75342802	0.614706102
Myh14	-0.0100049	0.031191085	0.75661032	0.614706102
Ndufa9	0.0058048	0.017829519	0.75310006	0.614706102
Pmm1	0.0317856	0.098702186	0.75567989	0.614706102
Rgs17	-0.0122694	0.03874078	0.75957471	0.614706102
Rpl35	-0.010332	0.032316207	0.7573727	0.614706102
Scn1b	0.00665298	0.02098534	0.75933642	0.614706102
Slc17a7	-0.007129	0.022659776	0.7611062	0.614706102
Snap47	0.00782486	0.024083045	0.75358183	0.614706102
Soga3	-0.0046796	0.014876874	0.76114646	0.614706102
Timm8b	0.01847566	0.057328256	0.75550255	0.614706102
Tjp1	-0.0238252	0.07504292	0.75900176	0.614706102
Ube2n	-0.0100622	0.031497335	0.757559	0.614706102
Dnpep	-0.0068366	0.022059849	0.76454617	0.615013115
Map2k1;Map2k2	0.013281	0.042739797	0.76393969	0.615013115
Mrs2	-0.0124856	0.040231086	0.76422823	0.615013115
Agpat5	0.01127658	0.036650976	0.76618754	0.615966594
Acad9	0.0059336	0.019379249	0.76728193	0.615995653
Bcap29	0.0159628	0.052514931	0.76890907	0.615995653
Crocc	0.01457026	0.047860065	0.76856558	0.615995653
Gas7	-0.0116509	0.038338443	0.7689602	0.615995653
Mtor	0.0056525	0.018566658	0.76855898	0.615995653
Rpl29	-0.0096636	0.031761483	0.76869724	0.615995653
Adam22	-0.0046726	0.015515856	0.7709789	0.616515785
Eif5a2;Eif5a	-0.0157306	0.052038271	0.77014169	0.616515785
Mrpl39	-0.0230538	0.076456405	0.77070053	0.616515785
Armc10	0.00723226	0.024167619	0.77237308	0.61726518
Gng2	-0.0172599	0.0578878	0.77317689	0.617542165
Ndufa11	0.0103354	0.034935199	0.77488237	0.617808291
Omg	0.0074476	0.025090155	0.77415439	0.617808291
Otol1	-0.0149222	0.050389655	0.77466786	0.617808291
Hspa5	-0.0073542	0.024954679	0.77572402	0.618114448
Klhl22	0.0353576	0.120900923	0.77738314	0.618450558
Pafah1b1	0.005507	0.018851307	0.7776211	0.618450558
Prpsap2	0.01158052	0.039671361	0.77778107	0.618450558
Ttc9b	0.0195034	0.06687376	0.77797745	0.618450558
Guk1	-0.008253	0.02842731	0.77895628	0.618500598

Rps18	0.007758	0.0267189	0.77892806	0.618500598
Fis1	0.01301734	0.045289553	0.7810918	0.619268981
Mtnd3	-0.0115219	0.04012586	0.78129953	0.619268981
Sirt5	0.0234364	0.081481384	0.78094109	0.619268981
Prkar2b	0.0132782	0.046350742	0.78179534	0.619298531
Ctsd	-0.0114519	0.040306211	0.78353244	0.620269249
Pkm	0.0105834	0.037321844	0.78393926	0.620269249
Rpn1	0.009544	0.033938795	0.78568413	0.621285863
Kif5c	0.0073342	0.026205612	0.78667638	0.621706491
Chmp6	0.0081232	0.029207985	0.78797628	0.621710515
Gde1	0.00954602	0.034338272	0.78806242	0.621710515
Lamtor1	-0.0182478	0.06563276	0.78804063	0.621710515
Arhgap1	0.01098209	0.039800794	0.78959821	0.622558475
Arf1;Arf3	-0.0101783	0.037042877	0.79045492	0.62271878
Hexa	0.014229	0.051971143	0.79118472	0.62271878
Pycr2	-0.0061329	0.022359122	0.79081197	0.62271878
Trim32	0.0344786	0.126357285	0.79186755	0.622893222
Apba1	-0.0093192	0.034421223	0.79344516	0.623770906
Psmb6	-0.0089082	0.033161091	0.79500882	0.624636599
Trap1	-0.0099752	0.037328294	0.79605276	0.625093181
Camsap3	-0.0070534	0.026562654	0.79731052	0.625717037
Ap3b2	-0.0064602	0.02464898	0.79987584	0.626637926
Ppib	0.0072207	0.027459446	0.79922789	0.626637926
Slc38a3	-0.014246	0.054307472	0.79970171	0.626637926
Fam136a	0.0079252	0.030347308	0.80057393	0.626821238
Ablim2	-0.0082136	0.03243939	0.80650005	0.627381952
Apoa1bp	0.0065614	0.026478644	0.8105311	0.627381952
Arfip2	-0.0137978	0.054429027	0.80627442	0.627381952
Atp5g1;Atp5g3;Atp5g2	-0.0090158	0.035508337	0.80597168	0.627381952
Cd200	0.01544633	0.061456529	0.80788881	0.627381952
Coq5	0.01475	0.059554826	0.81062731	0.627381952
Coro1c	0.0051448	0.020620202	0.80925871	0.627381952
Csnk2b	-0.0090094	0.03586422	0.80798463	0.627381952
Ctbp1;Ctbp2	0.0099696	0.040344436	0.81104491	0.627381952
Fabp5	0.01190664	0.047119168	0.80687781	0.627381952
Ildr2	-0.0074992	0.029701272	0.80702946	0.627381952
L2hgdh	0.00701269	0.028166998	0.809658	0.627381952
Nefl	0.0225592	0.087106823	0.80218748	0.627381952
Pgp	0.00603932	0.024394482	0.81070342	0.627381952
Pgrmc2	0.0072107	0.028658163	0.80768494	0.627381952
Ppp1r12a	0.0122013	0.048930611	0.809366	0.627381952
Ptn	-0.0151167	0.059746257	0.80663734	0.627381952
Rpl28	-0.0080346	0.032483376	0.81087021	0.627381952
Src	0.00910422	0.035569036	0.80444099	0.627381952
Synj1	-0.002936	0.011382377	0.80296269	0.627381952
Wdr7	0.005978	0.023354224	0.80443228	0.627381952
Coro1b	-0.0090432	0.036702127	0.81158136	0.627437566
Etfdh	-0.0044044	0.017931493	0.81215756	0.627523823
Snx27	-0.0095485	0.039162515	0.81350778	0.627848729
Thsd7a	-0.0200738	0.082255736	0.81334094	0.627848729
Gak	0.01084146	0.044583915	0.81399297	0.627864408
Rhoa;Rhoc	-0.0064097	0.026644176	0.81594072	0.629007554
Clu	-0.008173	0.034574051	0.81906927	0.629762822
Mif	0.0076474	0.032383984	0.81925184	0.629762822
Nf1	0.00808567	0.034100919	0.81853054	0.629762822
Ptges2	-0.0102628	0.043394967	0.81898958	0.629762822
Rab8b	0.00891656	0.037742839	0.81917876	0.629762822
Dmxl2	0.0044852	0.019300516	0.82207173	0.631510085
Etfa	0.0056608	0.024479437	0.82292755	0.631510085
Gnaq	-0.003622	0.015658976	0.8228841	0.631510085
Aldh18a1	0.0137482	0.062417735	0.83118472	0.631727591
Atl2	-0.0080819	0.035430333	0.82528466	0.631727591
Brsk2	-0.0139914	0.062700884	0.82901483	0.631727591
Cacna2d2	-0.009521	0.044473865	0.83583979	0.631727591
Cecr6	-0.015766	0.069785439	0.82692781	0.631727591
Cyld	0.00853112	0.039463366	0.8342592	0.631727591

Enpp6	0.024087	0.111624373	0.83455456	0.631727591
Fkbp4	-0.0109234	0.048827248	0.8285858	0.631727591
Gng3	0.00526204	0.023574539	0.82896713	0.631727591
Grid1	-0.0281964	0.130733603	0.83463587	0.631727591
Hspd1	0.0040136	0.018014425	0.82927503	0.631727591
Hspe1	0.0044996	0.020822598	0.83432405	0.631727591
Marcksl1	0.0130702	0.060116335	0.8333275	0.631727591
Mgll	0.01290658	0.05723136	0.82723236	0.631727591
Mink1	-0.00668	0.029384874	0.82587069	0.631727591
Pam16	-0.0042436	0.019742718	0.83518991	0.631727591
Ppif	0.0050482	0.022389474	0.82726532	0.631727591
Prdx4	0.0114946	0.053642542	0.83568856	0.631727591
Rab11fip2	-0.0132666	0.06192898	0.83573274	0.631727591
Rhog	0.0097036	0.044707462	0.83360509	0.631727591
Rplp2	0.01017624	0.046885315	0.83360598	0.631727591
Scamp3	-0.0042276	0.018633953	0.82621086	0.631727591
Sh2d5	-0.0083818	0.036912768	0.82606406	0.631727591
Shroom2	0.0097214	0.042604624	0.8252327	0.631727591
Snx1	-0.0074884	0.034352591	0.83289718	0.631727591
Snx4	-0.0177718	0.079989905	0.82974419	0.631727591
Vamp2	0.0087574	0.039836722	0.83150744	0.631727591
Trappc3	0.00727772	0.03410821	0.83637447	0.63177816
Sorcs2	0.00502724	0.023687369	0.83723294	0.632039856
Them4	-0.0088587	0.041851486	0.83765684	0.632039856
Prdx6	-0.0055466	0.026347	0.8385241	0.632340965
Atp6v1c1	-0.0026498	0.012825889	0.84148526	0.633388689
Mccc1	-0.0085847	0.041574761	0.84156795	0.633388689
Plcb1	0.004296	0.020834632	0.8417893	0.633388689
Wasl	0.00680238	0.032959861	0.84164686	0.633388689
Yars	0.0048512	0.024155968	0.84584472	0.636085753
Spred1	-0.0092172	0.046320891	0.84723804	0.636778992
Abr	0.01257976	0.065453801	0.85238003	0.637523638
Acaa2	-0.0057462	0.029328	0.84955116	0.637523638
Dhcr7	-0.0059396	0.030546083	0.85067329	0.637523638
Etf1	-0.0181184	0.093506839	0.85118898	0.637523638
Gng7	0.02026483	0.103854005	0.85015728	0.637523638
Icam5	0.004746	0.024790814	0.85294905	0.637523638
Ncstn	-0.0069982	0.035809282	0.8499289	0.637523638
Nudt3	-0.0036336	0.018614544	0.85010199	0.637523638
Osbpl6	0.0104354	0.05442933	0.85273542	0.637523638
Thnsl1	-0.0051671	0.026781996	0.85181978	0.637523638
Wdr1	0.0071476	0.037946228	0.85528384	0.638915163
Psmd2	-0.0055331	0.029517832	0.85597576	0.639078569
Hibadh	0.008606	0.047521049	0.86079413	0.641995767
Ryr2	0.0052054	0.028786057	0.86099769	0.641995767
Vapa	0.005582	0.030938817	0.86130903	0.641995767
Clip1	0.0074262	0.04213972	0.86449418	0.642306631
Cntn2	0.0045776	0.025678402	0.86294516	0.642306631
Dtd1	0.00580544	0.032833934	0.86405022	0.642306631
Ist1	0.0046526	0.026425682	0.86461918	0.642306631
Prkce	0.00600362	0.034210586	0.86505505	0.642306631
Rad23b	-0.0081554	0.046016722	0.8637351	0.642306631
Rapgef2	0.0042234	0.023773482	0.86341229	0.642306631
Dnaja2	0.0044264	0.025520541	0.86661026	0.643107824
Ddx3x	-0.0089195	0.051855073	0.86770238	0.64317003
Fmn2	0.00582164	0.033933109	0.86804195	0.64317003
Pc	-0.0038958	0.022721853	0.8681227	0.64317003
Lrrc59	0.0071306	0.042338566	0.87043414	0.643375492
Phyhipl	-0.0067861	0.040307107	0.87047924	0.643375492
Psmd13	0.0059524	0.035439007	0.87078181	0.643375492
Rps5	0.00499932	0.029628476	0.87019432	0.643375492
Snap29	-0.0036959	0.021848482	0.86986943	0.643375492
Aldh3a2	-0.0082016	0.050030608	0.8738509	0.643573519
Dnm2	-0.0074669	0.045570131	0.87390886	0.643573519
Mrpl1	0.021875	0.131545014	0.87205247	0.643573519
Mrps27	-0.0047726	0.029037411	0.87352482	0.643573519

Prkar1a	-0.0098697	0.059858775	0.8731264	0.643573519
Prps1	0.00813364	0.049345879	0.87316722	0.643573519
Calb1	-0.0080766	0.049940371	0.8755324	0.644417771
Atp6v1g1	-0.0050444	0.03179742	0.87788176	0.645443495
Kbtbd11	-0.0031257	0.019642077	0.87750906	0.645443495
Actr3b	0.002258	0.014348443	0.87885341	0.645538271
Dars	0.0065484	0.041651031	0.87896659	0.645538271
Clybl	0.00585475	0.037556858	0.87998144	0.645721658
Cox5b	0.0032916	0.021148831	0.88017248	0.645721658
Nwd2	-0.015375	0.09967115	0.88122756	0.646144722
Bmpr2	-0.0097172	0.06425979	0.88354781	0.646792601
Capzb	-0.0027908	0.018448049	0.8835009	0.646792601
Tnik	0.0186634	0.122641298	0.88281387	0.646792601
Clta	0.0075864	0.050987251	0.8854015	0.647798461
Ptprz1	0.0089056	0.060625279	0.88684868	0.648505979
Adcy5	0.0144915	0.100172991	0.88855371	0.649221797
Ufc1	-0.0173814	0.120405669	0.88878895	0.649221797
Decr1	0.00306474	0.021566991	0.890512	0.649777565
Rps20	0.00278224	0.019530635	0.89024292	0.649777565
Exoc8	-0.004644	0.033060985	0.89176235	0.649793339
Them6	0.0093578	0.066751892	0.89197694	0.649793339
Wdr13	-0.0050343	0.035891568	0.89191769	0.649793339
Pip4k2a	-0.002779	0.020211911	0.8940385	0.65094405
Dpp6	-0.0034166	0.025234896	0.89564673	0.651160066
Hsd17b12	0.004782	0.035043593	0.89483024	0.651160066
Rps14	0.00455952	0.03372033	0.89578155	0.651160066
Lrp1	0.0019816	0.014752757	0.89646679	0.651307638
Gsr	-0.0038674	0.029082476	0.89749292	0.651360847
Hadhb	-0.0063394	0.048299196	0.89881583	0.651360847
Hrsp12	0.0081332	0.062049425	0.89895138	0.651360847
Itpka	-0.0059767	0.045425084	0.89857039	0.651360847
Rell2	0.005921	0.045161848	0.89892833	0.651360847
Fam126b	-0.0027048	0.021112572	0.90122136	0.651956346
Pcbp2	-0.0040555	0.031626623	0.90113179	0.651956346
Scn2b	-0.0046446	0.03603763	0.90063234	0.651956346
Hspa1l	0.01180534	0.09455771	0.9037241	0.653416878
Acap2	-0.0102976	0.083336926	0.90470713	0.653777645
Hspa4l	0.00185	0.015138852	0.90575293	0.653833724
Tln2	0.0020104	0.016389974	0.90540151	0.653833724
Cask	-0.0055716	0.046737115	0.90804704	0.6549808
Gng10	0.0058162	0.048930604	0.90831187	0.6549808
Rpl6	0.002667	0.022683922	0.90930485	0.655346943
Map2k7	0.0059678	0.051908282	0.91130361	0.656437184
Ndufaf7	-0.0129918	0.11524823	0.91302292	0.656625055
Pdk2	-0.0062008	0.054702395	0.91254198	0.656625055
Ptges3	0.0050692	0.044923143	0.91293633	0.656625055
Acsf3	-0.005854	0.055289208	0.91828464	0.657049282
Dnajb1	-0.0059778	0.057594466	0.91988985	0.657049282
Dnm1	0.0023198	0.021695872	0.91748237	0.657049282
Farp1	-0.0048166	0.046963737	0.92083641	0.657049282
Fech	-0.0021662	0.020344896	0.91782794	0.657049282
Grhpr	-0.0050213	0.045959844	0.9156921	0.657049282
Gsn	-0.0024445	0.023857302	0.92091003	0.657049282
Idh1	0.0046408	0.044806106	0.92005594	0.657049282
Impa1	0.00403072	0.03891851	0.9200613	0.657049282
Map2	0.0079816	0.077190224	0.9201893	0.657049282
Myh10	-0.003684	0.033745639	0.91575661	0.657049282
Ndufaf5	0.0057092	0.052907548	0.91672517	0.657049282
Pak1	-0.0030416	0.02782504	0.91564756	0.657049282
Shisa6	-0.0057802	0.055001723	0.91889115	0.657049282
Spg7	0.005701	0.054937648	0.91990448	0.657049282
Ndufa12	0.0021276	0.0225066	0.9270112	0.661053129
C2cd4cC2CD4 family	-0.005219	0.055905224	0.92791764	0.661350328
Prkg2;Prkg1	-0.0063086	0.068918307	0.92931622	0.661997789
Dlgap3	0.0022774	0.025931856	0.93217633	0.663106648
Ptpn11	-0.0015517	0.017494784	0.93150535	0.663106648

Ssr4	0.0043164	0.049272571	0.93234574	0.663106648
Cct7	0.0037762	0.043890641	0.9335517	0.663483252
Nefm	0.006782	0.079192697	0.93385775	0.663483252
Pgls	0.0064538	0.077340832	0.935547	0.664333959
Trp53i11	0.00261512	0.031877811	0.93663366	0.664756099
Adrm1	0.0035382	0.043915465	0.93776427	0.665063401
Gga3	0.004407	0.054953055	0.93805148	0.665063401
Gng13	0.003801	0.047812723	0.93858942	0.665095662
Eif2s1	0.0025448	0.033002275	0.94042981	0.665757131
Prrt3	0.0026404	0.034287604	0.94050875	0.665757131
Smpd3	-0.0022384	0.030004426	0.94236285	0.666371086
Ube2m	0.00273418	0.036431981	0.94201855	0.666371086
Cd82	-0.0066027	0.092508198	0.94485216	0.667605906
Kcnab2	0.00282216	0.040447271	0.94608628	0.667605906
Rab21	-0.0040324	0.057595819	0.94590255	0.667605906
Ralb	0.003291	0.046521318	0.94533974	0.667605906
Kif1a	0.00194092	0.029639302	0.94939497	0.669241366
Syt3	0.00221792	0.033707835	0.94915296	0.669241366
Eif4a2	-0.0023564	0.039251359	0.9536007	0.669970527
Map1b	0.0018286	0.030298544	0.95335517	0.669970527
Por	-0.001366	0.022702863	0.95349727	0.669970527
Ppfia3	-0.0009874	0.016554717	0.95390171	0.669970527
Rab33b	-0.002385	0.037668583	0.9510688	0.669970527
Slc2a13	0.00130743	0.021769611	0.95358298	0.669970527
Tnr	0.0018412	0.030405647	0.95319941	0.669970527
Ak4	-0.0015236	0.027199007	0.95670223	0.67019489
Ap3s2	-0.001553	0.027230746	0.95591912	0.67019489
Camk4	-0.0078802	0.13985549	0.95644858	0.67019489
Fkbp1b	0.0022134	0.038214912	0.9552331	0.67019489
Slc8a2	-0.0009766	0.016782817	0.95502416	0.67019489
Aldh5a1	0.0015942	0.032293536	0.96183793	0.670382289
Camk2b	0.0010532	0.020646441	0.96056727	0.670382289
Erc1	0.0026138	0.051527546	0.96078732	0.670382289
Fabp3	0.001953	0.042068409	0.96410997	0.670382289
Hyou1	-0.000743	0.015507739	0.96296263	0.670382289
Iqsec3	-0.0021028	0.038454612	0.95773224	0.670382289
Kif20b	-0.0062748	0.125712798	0.96141481	0.670382289
Ndrg3	0.00152294	0.030129565	0.96092626	0.670382289
Rab5c	0.0011624	0.025018497	0.96408121	0.670382289
Rpl37a	-0.0027076	0.0588232	0.96441504	0.670382289
Srm	0.0018698	0.036686215	0.96060112	0.670382289
Strap	0.0020788	0.044787617	0.96411752	0.670382289
Strn	-0.0021511	0.043496924	0.96177056	0.670382289
Stx8	0.0028832	0.061190229	0.9635737	0.670382289
Tbc1d24	0.0014604	0.027430819	0.95884663	0.670382289
Tbcb	0.00108404	0.024190935	0.96535566	0.670690948
Ahsa1	0.00184428	0.048212638	0.97042329	0.670781645
Arpc5	-0.001412	0.03799847	0.97126844	0.670781645
Atp8a1	-0.0011108	0.02571121	0.96659862	0.670781645
Eif4h	-0.0009416	0.023252978	0.96869185	0.670781645
Gdap1l1	-0.0021176	0.058045863	0.97179285	0.670781645
Micu1	0.00144544	0.038061167	0.97063678	0.670781645
Mpp1	-0.003694	0.096401242	0.97037231	0.670781645
Ndufa3	-0.0008698	0.023970583	0.97194265	0.670781645
Rab39a	0.0028018	0.065425814	0.96689133	0.670781645
Rftn2	0.001256	0.032212835	0.96985329	0.670781645
Stim2	0.0041342	0.098914962	0.96768603	0.670781645
Stx7	0.0007522	0.019095188	0.96954307	0.670781645
Wdr47	-0.0015276	0.03734462	0.9683737	0.670781645
Arl3	0.0012564	0.037311824	0.97396292	0.670804841
Erp44	0.0005717	0.016902332	0.9738464	0.670804841
Lysmd1	0.0020656	0.058436208	0.97266837	0.670804841
Syt17	-0.0017078	0.050343973	0.97376996	0.670804841
Eef1d	0.00132642	0.04190349	0.97552333	0.671195006
Reep2	-0.0012801	0.039958763	0.97522933	0.671195006
Actr1a	0.000601	0.019588244	0.97627505	0.671285749

Psma7;Psma8	0.0015628	0.05175253	0.97664926	0.671285749
Prdx1	0.0005282	0.018929514	0.97842263	0.672162578
Crk	-0.0021616	0.120961725	0.98618012	0.67335189
Etfb	-0.000573	0.033156193	0.98663501	0.67335189
Gcsh	-0.0005025	0.028666195	0.98644404	0.67335189
Ppa1	0.00053312	0.02358362	0.98251861	0.67335189
Prrt1	0.0006026	0.029558001	0.98423394	0.67335189
Rps8	0.00039394	0.017016453	0.98209722	0.67335189
Sacm1l	0.000471	0.024436359	0.98509413	0.67335189
Scamp4	0.00038556	0.020837443	0.98569056	0.67335189
Sec62	0.00092664	0.044008573	0.98371676	0.67335189
Snd1	-0.000859	0.045376045	0.98535934	0.67335189
Tsfm	0.00133816	0.071616195	0.98554988	0.67335189
Twf1	0.001023	0.050812652	0.98443053	0.67335189
Xpr1	-0.0020704	0.117048913	0.98632068	0.67335189
Kctd12	-0.0007554	0.05040466	0.9884098	0.674222447
Lzts3	-0.0005886	0.049779803	0.99085553	0.675549561
Hdhd2	-0.0005153	0.049223721	0.99190428	0.675923386
Gdpd1	-0.0003164	0.042041263	0.99417952	0.676449929
Prosc	0.00013672	0.01805019	0.99414201	0.676449929
Rab5a	-0.0002442	0.032440006	0.9941786	0.676449929
Htra2	0.0007426	0.123811035	0.99536131	0.676572347
Nceh1	-0.0001217	0.018902845	0.99501994	0.676572347
Rpl18a	-0.000123	0.029371226	0.99676067	0.677182723
Pgm2l1	0.00011723	0.037690395	0.99759455	0.677408502
Cnr1	6.12E-05	0.026364925	0.99820474	0.677482229
Sgip1	1.38E-05	0.012739656	0.99916223	0.67779148
Cap2	1.86E-05	0.044159122	0.99967424	0.677798377

Table S1. Statistical results of the synaptoneurosome proteomics data.

	BETA ESTIMATE	STANDARD ERROR	*P* VALUE
**CONTROL VS. RRMS AND PPMS**			
Intercept	0.08	0.04	0.08
RRMS	0.07	0.03	0.03
PPMS	< 0.01	0.05	0.98
Sex	0.01	0.02	0.7
Age	< 0.01	< 0.01	0.37
EDSS	0.01	0.01	0.47
Disease duration	< 0.01	0.01	0.96
Immunotherapy	0.05	0.06	0.4
High-efficient DMT	NA	NA	NA
**RRMS VS PPMS**			
Intercept	0.14	0.08	0.08
PPMS	-0.07	0.04	0.09
Sex	< 0.01	0.03	0.98
Age	< 0.01	< 0.01	0.7
EDSS	0.01	0.02	0.54
Disease duration	< 0.01	0.01	0.97
Immunotherapy	0.05	0.08	0.52
High-efficient DMT	NA	NA	NA

Abbreviations: DMT = Disease modifying treatment, EDSS = expanded disability status scale.

Table S2. Statistical results of linear model with CSF BSN.

	BETA ESTIMATE	STANDARD ERROR	*P* VALUE
**CONTROL VS RRMS AND SPMS AND PPMS**			
Intercept	-0.005	0.039	0.888
RRMS	0.028	0.024	0.243
SPMS	0.07	0.035	0.046
PPMS	0.099	0.033	0.003
Sex	0.017	0.016	0.283
Age	0.001	0.001	0.426
Disease duration	< 0.001	0.002	0.801
EDSS	-0.005	0.006	0.426
Immunotherapy	-0.068	0.076	0.376
High-efficient DMT	0.202	0.092	0.031
**RRMS VS SPMS AND PPMS**			
SPMS	0.007	0.058	0.899
PPMS	0.022	0.067	0.748
Sex	0.034	0.025	0.176
Age	0.003	0.003	0.274
Disease duration	-0.001	0.002	0.777
EDSS	-0.006	0.008	0.425
Immunotherapy	-0.058	0.093	0.531
High-efficient DMT	0.191	0.113	0.095
**SPMS VS PPMS**			
Intercept	0.002	0.159	0.989
PPMS	0.019	0.039	0.626
Sex	0.032	0.035	0.367
Age	0.002	0.003	0.535
Disease duration	< 0.001	0.002	0.96
EDSS	-0.007	0.01	0.471
Immunotherapy	-0.058	0.106	0.59
High-efficient DMT	0.207	0.148	0.173

Abbreviations: DMT = Disease modifying treatment, EDSS = expanded disability status scale.

Table S3. Statistical results of linear model with serum BSN where values below background were replaced with zero.

	BETA ESTIMATE	STANDARD ERROR	*P* VALUE
**CONTROL VS RRMS AND SPMS AND PPMS**			
Intercept	0.043	0.034	0.212
RRMS	0.017	0.021	0.41
SPMS	0.073	0.03	0.018
PPMS	0.099	0.029	0.001
Sex	-0.001	0.014	0.928
Age	< 0.001	0.001	0.741
Disease duration	-0.001	0.001	0.51
EDSS	-0.003	0.006	0.552
Immunotherapy	-0.03	0.066	0.649
High-efficient DMT	0.147	0.08	0.07
**RRMS VS SPMS AND PPMS**			
SPMS	-0.016	0.068	0.815
PPMS	0.013	0.051	0.804
Sex	0.023	0.058	0.69
Age	0.01	0.022	0.634
Disease duration	0.003	0.002	0.217
EDSS	-0.001	0.002	0.553
Immunotherapy	-0.005	0.007	0.497
High-efficient DMT	-0.022	0.081	0.789
**SPMS VS PPMS**			
Intercept	0.038	0.136	0.782
PPMS	0.016	0.034	0.632
Sex	0.007	0.029	0.811
Age	0.002	0.003	0.484
Disease duration	-0.001	0.002	0.734
EDSS	-0.006	0.008	0.498
Immunotherapy	-0.021	0.09	0.814
High-efficient DMT	0.14	0.127	0.278

Abbreviations: DMT = Disease modifying treatment, EDSS = expanded disability status scale.

Table S4. Statistical results of linear model with serum BSN where values below background were replaced with half of the limit of detection.

	BETA ESTIMATE	STANDARD ERROR	*P* VALUE
**CONTROL VS RRMS AND SPMS AND PPMS**			
Intercept	-0.008	0.04	0.838
RRMS	0.041	0.025	0.095
SPMS	0.097	0.036	0.008
PPMS	0.114	0.034	0.001
Sex	-0.015	0.016	0.364
Age	0.001	0.001	0.115
Disease duration	-0.003	0.002	0.097
EDSS	-0.001	0.007	0.911
Immunotherapy	-0.112	0.078	0.153
High-efficient DMT	0.213	0.095	0.027
**RRMS VS SPMS AND PPMS**			
SPMS	-0.081	0.077	0.3
PPMS	-0.013	0.058	0.828
Sex	-0.015	0.066	0.817
Age	-0.01	0.025	0.687
Disease duration	0.005	0.002	0.038
EDSS	-0.003	0.002	0.158
Immunotherapy	-0.003	0.008	0.727
High-efficient DMT	-0.103	0.092	0.268
**SPMS VS PPMS**			
Intercept	-0.013	0.151	0.932
PPMS	0.008	0.037	0.831
Sex	-0.024	0.033	0.472
Age	0.004	0.003	0.184
Disease duration	-0.002	0.002	0.319
EDSS	-0.008	0.009	0.398
Immunotherapy	-0.101	0.1	0.32
High-efficient DMT	0.172	0.141	0.231

Abbreviations: DMT = Disease modifying treatment, EDSS = expanded disability status scale.

Table S5. Statistical results of linear model with serum BSN where values below background were imputed.

BIOMARKER	PARAMETER	ST. BETA	CI 95%	CI 5%	*P* VALUE
sBSN	Intercept	-0.04	-0.37	0.29	0.05
sBSN	Time	0.09	-0.07	0.26	0.27
sBSN	Age	-0.18	-0.36	0.01	0.07
sBSN	Sex at birth	0.05	-0.35	0.46	0.79
sBSN	Baseline	0.6	0.41	0.79	< 0.01
sNfL	Intercept	0.23	-0.23	0.7	0.19
sNfL	Time	-0.08	-0.23	0.07	0.29
sNfL	Age	0.23	-0.01	0.47	0.07
sNfL	Sex at birth	-0.34	-0.88	0.21	0.23
sNfL	Baseline	0.82	0.58	1.06	< 0.01
EDSS	Intercept	0.14	-0.27	0.56	0.08
EDSS	Time	0.17	0.05	0.28	< 0.01
EDSS	Age	-0.17	-0.4	0.07	0.18
EDSS	Sex at birth	-0.18	-0.67	0.3	0.46
EDSS	Baseline	0.81	0.59	1.03	< 0.01
Rel. cortex volume	Intercept	0.13	-0.05	0.31	0.4
Rel. cortex volume	Time	-0.07	-0.15	0.02	0.14
Rel. cortex volume	Age	0.01	-0.1	0.12	0.85
Rel. cortex volume	Sex at birth	-0.18	-0.4	0.03	0.11
Rel. cortex volume	Baseline	0.97	0.86	1.08	< 0.01

Abbreviations: CI = confidence interval, EDSS = expanded disability status scale, Rel. = relative, sBSN = serum bassoon, sNfL = serum neurofilament light chain, St. = standardised

Table S6. Statistical results of longitudinal models.
